# Screening of a Novel Fragment Library with Functional Complexity against *Mycobacterium tuberculosis* InhA

**DOI:** 10.1002/cmdc.201700774

**Published:** 2018-02-19

**Authors:** Federica Prati, Fabio Zuccotto, Daniel Fletcher, Maire A. Convery, Raquel Fernandez‐Menendez, Robert Bates, Lourdes Encinas, Jingkun Zeng, Chun‐wa Chung, Paco De Dios Anton, Alfonso Mendoza‐Losana, Claire Mackenzie, Simon R. Green, Margaret Huggett, David Barros, Paul G. Wyatt, Peter C. Ray

**Affiliations:** ^1^ Drug Discovery Unit, College of Life Sciences University of Dundee Dow Street Dundee DD1 5EH Scotland UK; ^2^ DPU TB Diseases of the Developing World Tres Cantos Medicines Development Campus GlaxoSmithKline Severo Ochoa 2 Tres Cantos 28760 Madrid Spain; ^3^ Platform Technology and Sciences Medicines Research Centre, GlaxoSmithKline Gunnels Wood Road Stevenage Herts SG1 2NY Hertfordshire UK

**Keywords:** fragment based drug discovery, functional group complexity, InhA, tuberculosis

## Abstract

Our findings reported herein provide support for the benefits of including functional group complexity (FGC) within fragments when screening against protein targets such as *Mycobacterium tuberculosis* InhA. We show that InhA fragment actives with FGC maintained their binding pose during elaboration. Furthermore, weak fragment hits with functional group handles also allowed for facile fragment elaboration to afford novel and potent InhA inhibitors with good ligand efficiency metrics for optimization.

We recently reported on the design and synthesis of a novel fragment library of diverse fragments which include functional group complexity (FGC).^1^ Functional groups,^2^ or “chemical handles”, were incorporated onto diverse scaffolds to allow for additional interactions with target proteins with the aim of maintaining binding poses during optimization, as well as aid fragment elaboration.^1^ However care has to be taken because increasing complexity in a fragment decreases the probability of it achieving optimal ligand‐protein interactions.^3^ Conversely, too little complexity can lead to interesting interactions being missed.^4^ In this respect, fragment deconstruction studies on β‐lactamase inhibitors,^5^ suggest that small fragments with “minimal complexity” derived from the deconstruction of potent inhibitors do not always retain the binding poses of the parent molecules. Whereas, fragments with built in FGC recapitulate the larger potent inhibitor binding mode. Therefore, a careful balance is required to identify a sweet spot of complexity where detectable, single‐mode binding of a ligand to a target is most probable. There is further support for this concept based on other fragment deconstruction case studies.^6^

Despite the molecular complexity model and its putative applications in fragment screening were introduced and refined by Hann^3^ and others over nearly two decades ago, to date the authors are not aware of any reported fragment screening against a novel diverse fragment library designed to include FGC. Our recently reported FGC library is based on synthetic chemistry toward selected functional groups.^1^ However, recent reports of an algorithm to identify all functional groups in organic molecules, allows for the analysis of FGC in large chemical databases of commercial fragments.^7^

Herein we describe the screening of such a library, in comparison with other fragment sets, against the highly validated *Mycobacterium tuberculosis* (Mtb) target InhA.^8^ Mtb is the causative agent of tuberculosis (TB), which is currently the leading infectious disease killer worldwide.^9^ Isoniazid (INH, Figure 1), a successful frontline TB drug for more than 50 years, targets the NADH‐dependent 2‐*trans* enoyl–acyl carrier protein (ACP) reductase InhA. This is a key enzyme in the Mtb cell wall synthesis pathway^10^ and does not have a human orthologue. The development of Mtb resistant strains to standard anti‐TB drugs,^11^ including INH, necessitates the need for novel Mtb targeted therapies. Resistance to INH develops mainly via mutations in the Mtb KatG enzyme, which converts INH into an acyl radical, which covalently binds to NADH and the resulting adduct inhibits InhA.^12^ Direct InhA inhibitors are envisaged to bypass this resistance mechanism and maintain clinical efficacy. Accordingly, there has been widespread research in this field^13^ and whilst limited set of potent direct InhA inhibitors with activity against INH‐resistant strains have been identified (**1**–**3** 14 in Figure 1), none have been progressed into clinical development. Hence, there remains a need to identify novel direct InhA inhibitor scaffolds.

**Figure 1:**
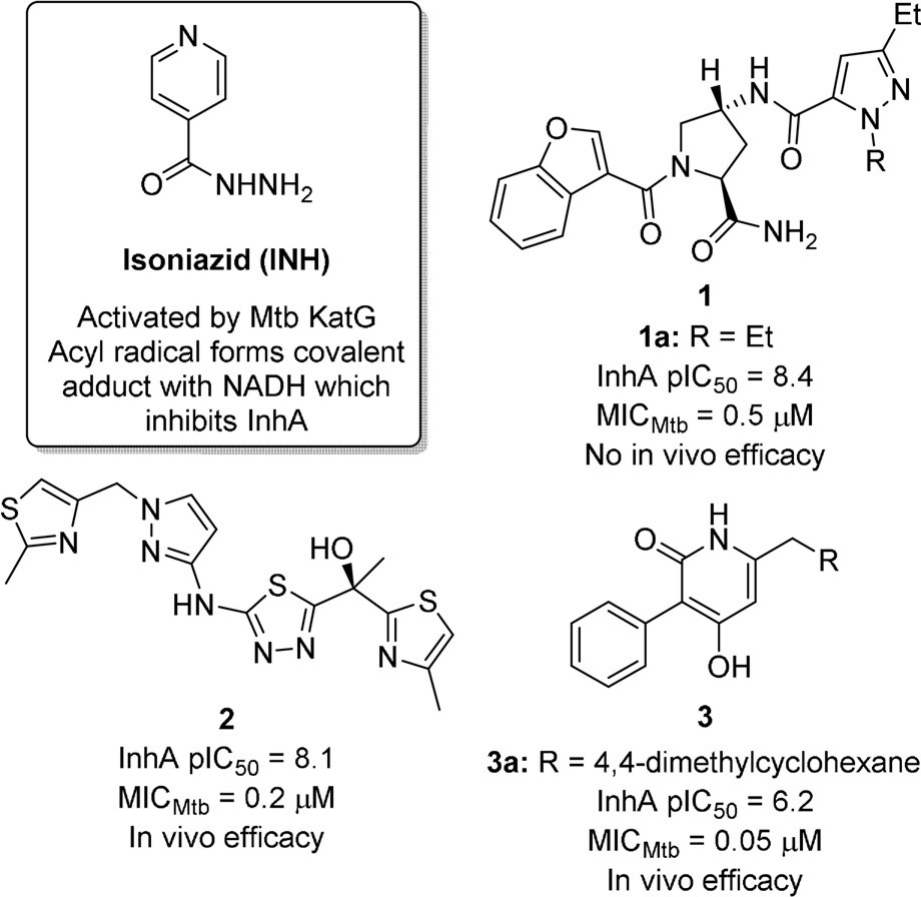
INH and selected advanced direct InhA inhibitors **1**–**3**.

InhA inhibitors are known to modulate the tertiary structure of the InhA protein binding pocket, in particular the substrate binding loop (SBL).^15^ In this respect, a fragment based (FB) approach^16^ was considered appealing in order to assess the InhA protein conformations for fragment actives and the structural requirements for their optimization into potent InhA inhibitors.

For the above reasons, we screened the recently reported FGC fragment library (FGC‐FRAG),^1^ as well as an informed InhA commercial fragment set (InhA‐INF‐FRAG), which was compiled based on the known direct InhA inhibitors in the public domain (see Supporting Information). The above libraries were screened alongside an historical commercial fragment library (HIST‐FRAG), a reported 3D fragment library (3D‐FRAG)^17^ and fragments derived from inventory (INV‐FRAG) and project (PROJ‐FRAG) sources. The overall library constituted 1360 fragments (Figure 2 A), which were screened against the NADH bound form of the InhA, using saturation transfer difference (STD) ^1^H NMR (complete results in Supporting Information).

**Figure 2:**
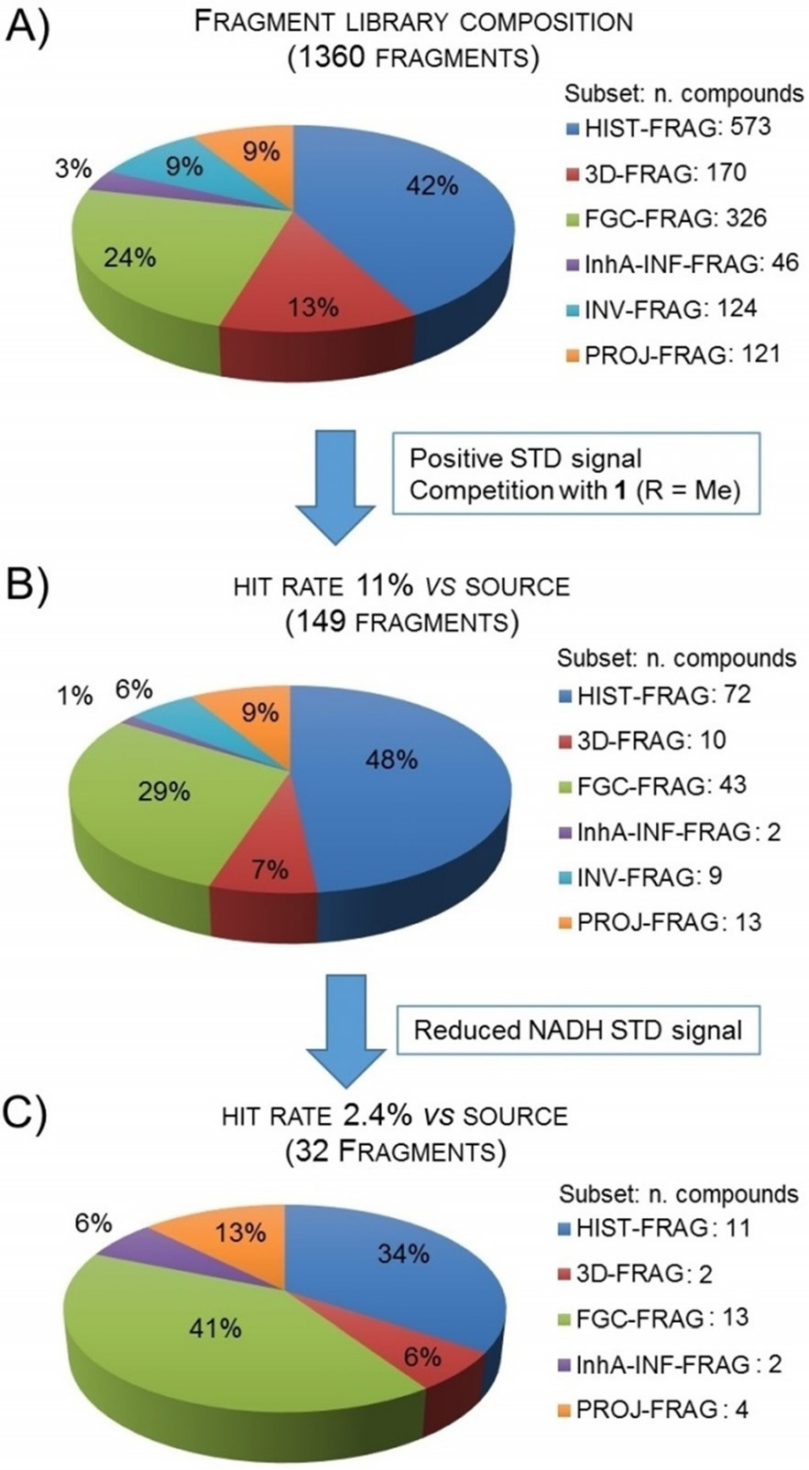
A) Fragment screening library composition. B) 149 STD‐NMR hits vs. their source. C) 32 STD‐NMR hits with reduction of NADH peak intensity vs. their source.

STD‐NMR typically identifies ligands that bind weakly to moderately to protein targets.^18^ The criteria for a binding event used here was a positive STD signal intensity which was decreased by at least 50 % on the addition of the known inhibitor **1** (R=Me).14a This resulted in 149 hits (11 % hit rate). A breakdown of these hits based on their source is given in Figure 2 B. Due to its binding affinity being in the suitable range (*K*_d_≈5 μm),^19^ NADH binding was also observed in the STD‐NMR spectra. It was noted that the stronger binders **1** (R=Me) and **3** (R=CH_2_ *i*Pr)14b decreased the STD‐NMR intensities for the NADH co‐factor peaks. Therefore, greater importance was given to those fragments which also caused a decrease in the NADH STD peak intensities, as this was considered as evidence of stronger binding. This further selection step decreased the number of hits to 32 (**4**–**35** in Figure 3; 2.4 % hit rate). The pie chart for the source of these 32 hits is given in Figure 2 C. This process increased the fraction of hits from the FGC‐FRAG set (29 % to 41 %). These data are interesting considering the FGC‐FRAG set only constituted 24 % of the whole screening library. The initial hit rate for the InhA‐INF‐FRAG set was low, although the size of the library was small. This may be the result of a lack of InhA fragment inhibitors that can be purchased from vendors, as observed for deconstruction of kinase inhibitors from the public domain.^20^ The two hits derived from this library did, however, survive the second selection step and could also be classified as FGC fragments. A high proportion of project, historical derived and 3D fragment actives were also noticeably enriched with FGC.

**Figure 3:**
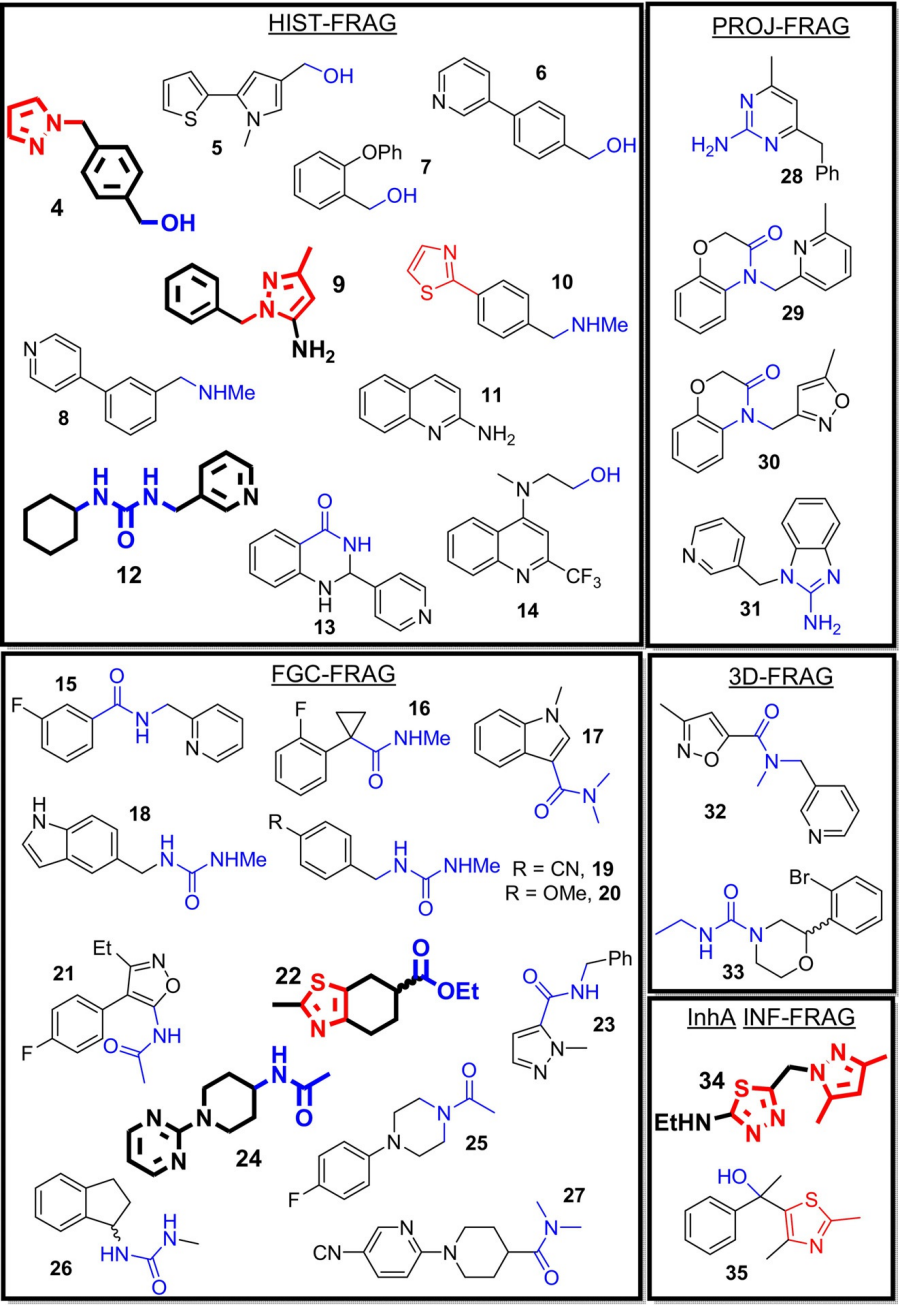
32 STD NMR hits **4**–**35**. FGC are in blue; known InhA cores are in red. Crystal structures were obtained for fragments in bold.

The 149 NMR hits were also screened in a high concentration (500 μm) biochemical assay. Only fragments **4** (13 %), **9** (37 %), **22** (11 %), and **34** (10 %) showed InhA inhibitory potencies <10 %, and were further tested in dose–response studies up to 1 mm (Table 1). Notably, these four fragment hits were all from the 32 compounds expected to be more potent based on NADH STD signal suppression. Based on these results as well as chemical diversity, 15 compounds were prioritized for crystallography studies. Crystals suitable for structure determination were obtained for fragments **4**, **9**, **12**, **22**, **24** and **34** bound to InhA.

**Table 1:** Biochemical, SPR and LE metrics for fragment hits and optimized InhA inhibitors.

Compound	InhA pIC_50_^[a][e]^ (% 500 μm)	PDB ID	SPR p*K*_d_^[f]^	InhA biochemical LE/LLE/LELP^[c]^
**1** (R=Me)	7.9±NA	–	ND	0.30/5.0/5.4
**3** (R=CH_2_ *i*Pr)	5.0±0.7^[d]^	–	4.4±0.07^[a]^	0.35/2.3/7.8
**4**	<3 (13 %)	http://www.rcsb.org/pdb/explore/explore.do?structureId=5OIC	NA^[a][g]^	–
**9**	3.4±0.23 (37 %)	http://www.rcsb.org/pdb/explore/explore.do?structureId=5OIF	3.3±0.08^[a]^	0.34/3.1/5.1
**12**	<3 (2 %)	http://www.rcsb.org/pdb/explore/explore.do?structureId=5OIL	NA^[a][g]^	–
**22**	3.1±0.05 (11 %)	http://www.rcsb.org/pdb/explore/explore.do?structureId=5OIM	NA^[a][g]^	0.28/0.5/9.3
**24**	<3 (0 %)	http://www.rcsb.org/pdb/explore/explore.do?structureId=5OIN	NA^[a]^	–
**34**	<3 (10 %)	http://www.rcsb.org/pdb/explore/explore.do?structureId=5OIO	ND	–
**36**	<3	–	ND	–
**37**	4.1±0.06	http://www.rcsb.org/pdb/explore/explore.do?structureId=5OIP	NA^[a][g]^	0.24/3.4/6.2
**38**	4.0±0.01	–	3.2±0.10^[a]^	0.30/1.7/7.5
**39**	5.0±0.14	–	4.7±0.03^[b]^	0.30/4.2/6.5
**40**	4.8±0.20	http://www.rcsb.org/pdb/explore/explore.do?structureId=5OIQ	4.6±0.02^[a]^	0.45/2.6/5.2
**41**	6.0±0.06	http://www.rcsb.org/pdb/explore/explore.do?structureId=5OIR	5.2±0.02^[b]^	0.39/4.4/6.9
**42**	6.5±0.03	–	NA^[a]^	0.33/4.6/13
**43**	5.2±0.06	–	3.7±0.10^[a]^	0.30/3.4/10
**44**	6.9±0.07	http://www.rcsb.org/pdb/explore/explore.do?structureId=4QXM	NA^[b]^	0.36/3.1/10
**45**	8.1±0.27	http://www.rcsb.org/pdb/explore/explore.do?structureId=5JFO	8.0±0.10^[b]^	0.40/4.7/9.4
**46**	6.3±0.25	http://www.rcsb.org/pdb/explore/explore.do?structureId=5OIS	6.3±0.10^[b]^	0.32/3.9/8.8
**47**	7.3±0.20	http://www.rcsb.org/pdb/explore/explore.do?structureId=5OIT	7.1±0.06^[a]^	0.36/5.1/8.2

[a] Compounds were tested up to 1 mm. [b] Compounds were tested up to 100 μm. [c] LE metrics were calculated using Stradrop in silico prediction software. [d] Data previously reported.14b [e] Data are the mean±SD of one independent experiment performed in duplicate. [f] Data are the mean±SD of two independent experiments, each performed in duplicate. [g] No *K*_d_ values are quoted, but some interaction was observed. ND=not determined, NA=not available.

Surface plasmon resonance (SPR) equilibrium dissociation binding constants (p*K*_d_) were also determined, and found to correlate with InhA biochemical pIC_50_ values (Table 1).

The InhA–NADH co‐crystal structures for FGC fragments **22** (pIC_50_=3.1) and **24** (pIC_50_<3) were found to be in accordance with the FGC‐FRAG set design principle, in that additional interactions were observed for their FG with the InhA protein (Figures S1 and 4A). The co‐crystal structure of **24** contains a unique tetramer with four slightly different binding sites. The following description is for one of these, details of the others are shown in Figure S2). The amide carbonyl FG of **24** forms an H‐bond interaction with the InhA Tyr158 residue and the NADH ribosyl‐20‐OH group, while the NH H‐bonds with Met199. Furthermore, the pyrimidine ring of **24** picks up an additional H‐bond interaction with the backbone NH of Met98, a feature also observed for the thiadiazole core of a close analogue (PDB ID: http://www.rcsb.org/pdb/explore/explore.do?structureId=4BQP) of advanced lead **2**, which, however, does not interact with Tyr158.^19^ Whereas lead **3 a** (PDB ID: http://www.rcsb.org/pdb/explore/explore.do?structureId=4R9S) has a similar H‐bond interaction with Tyr158 and the NADH ribosyl‐20‐OH group. FGC fragment **24** is able to identify distinct interactions associated with both leads **2** and **3 a** (Figure 4 A).

**Figure 4:**
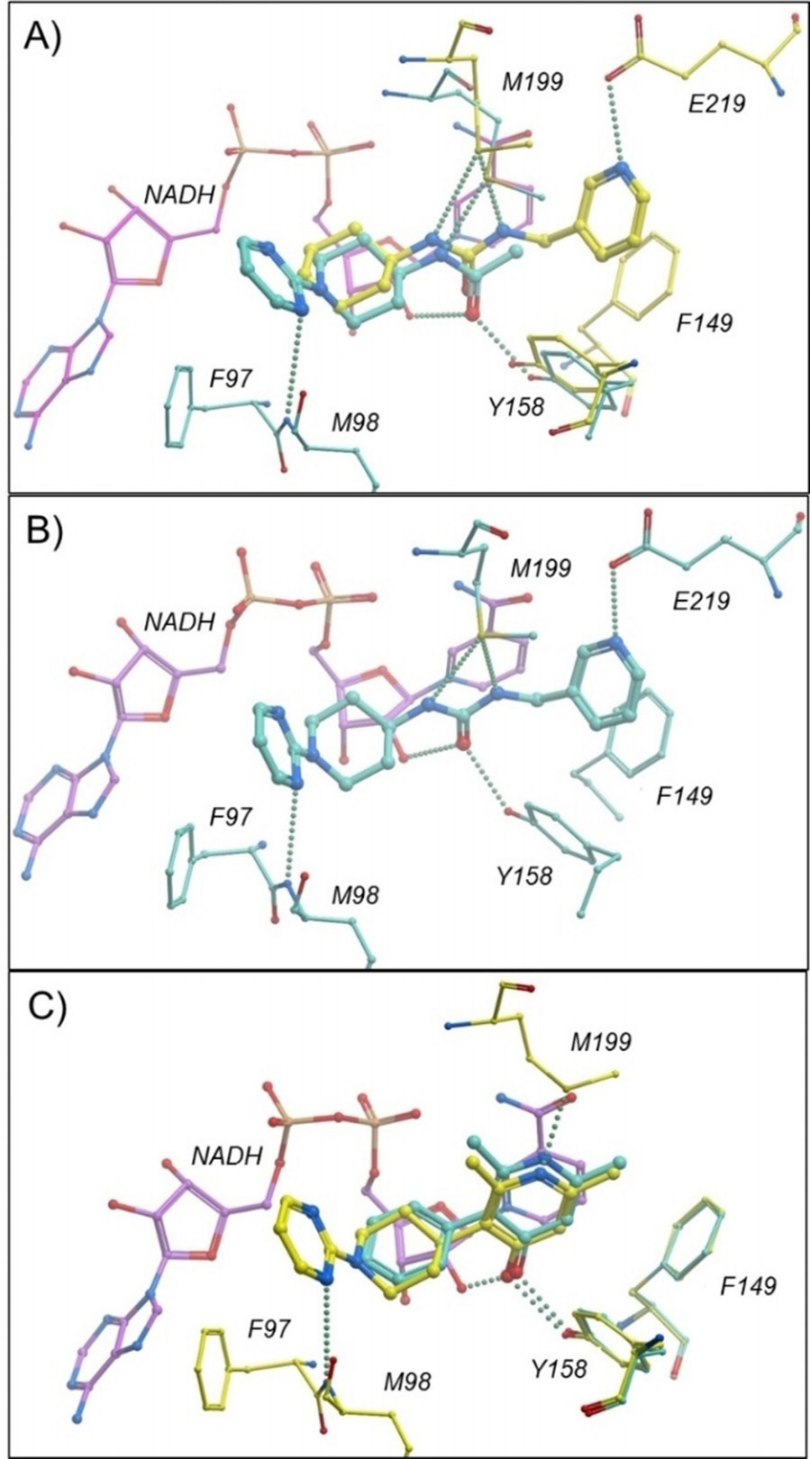
Crystal structures showing novel InhA–NADH–ligand complexes: A) overlay for fragments **12** (yellow) and **24** (blue), B) merged urea lead **37**, and C) overlay for fragment **40** (blue) and fused lead **41** (yellow).

Fragment **24** was found to overlay well with HIST‐FRAG urea **12** (pIC_50_<3.0), with the embedded urea FG having similar interactions with Tyr158, Met199 and the NADH ribosyl hydroxy group (Figure 4 A). Furthermore, the pyridyl group of **12** occupies the same space as the lipophilic di‐methyl cyclohexane group in lead **3 a** (Figure S3), however, its nitrogen is predicted to be protonated and binds to the carboxylic moiety of Glu219. Overlay of the urea **12** with the amide **24** led to synthesis of the merged amide **36** and urea **37** (Figure 5 and Scheme S1). Amide **36** was inactive in the biochemical screen (pIC_50_<3), but urea **37** had improved InhA biochemical potency (pIC_50_=4.1) over parent fragments. The crystal structure of InhA–NADH–ligand **37** complex indicated that the pose of the individual fragment components was conserved (Figure 4 B). Preparation of **38**, a simple methylated derivative of **12** resulted in improved enzymatic activity (pIC_50_=4.0), which transferred onto the pyrimidine series through synthesis of urea **39** (pIC_50_=5.0) (Figure 5 and Scheme S1). Advanced lead **3 a** was overlaid with urea **37** (Figure S4), and the GSK inventory searched to identify core fragment replacements of **3 a**. This led to the identification of pyridinone **40** 21 (Figure 5), with good biochemical potency (pIC_50_=4.8). The co‐crystal structure of **40** showed the conserved functional groups overlaid with both the advanced leads **3 a** and urea **37** (Figure S5). Replacement of the phenyl group of **40** with the novel pyrimidine fragment afforded compound **41** (Figure 5 and Scheme S2), with improved enzymatic activity (pIC_50_=6.0). Once again, the overall pose of the individual FGC fragment components of **40** and **41** was conserved (Figure 4 C). The novel pyrimidines **42** and **43** (Figure 5 and Scheme S3) were also prepared, based on **3 a** (pIC_50_=6.2), and showed good InhA biochemical potency (pIC_50_=6.5 and 5.2, respectively).

**Figure 5:**
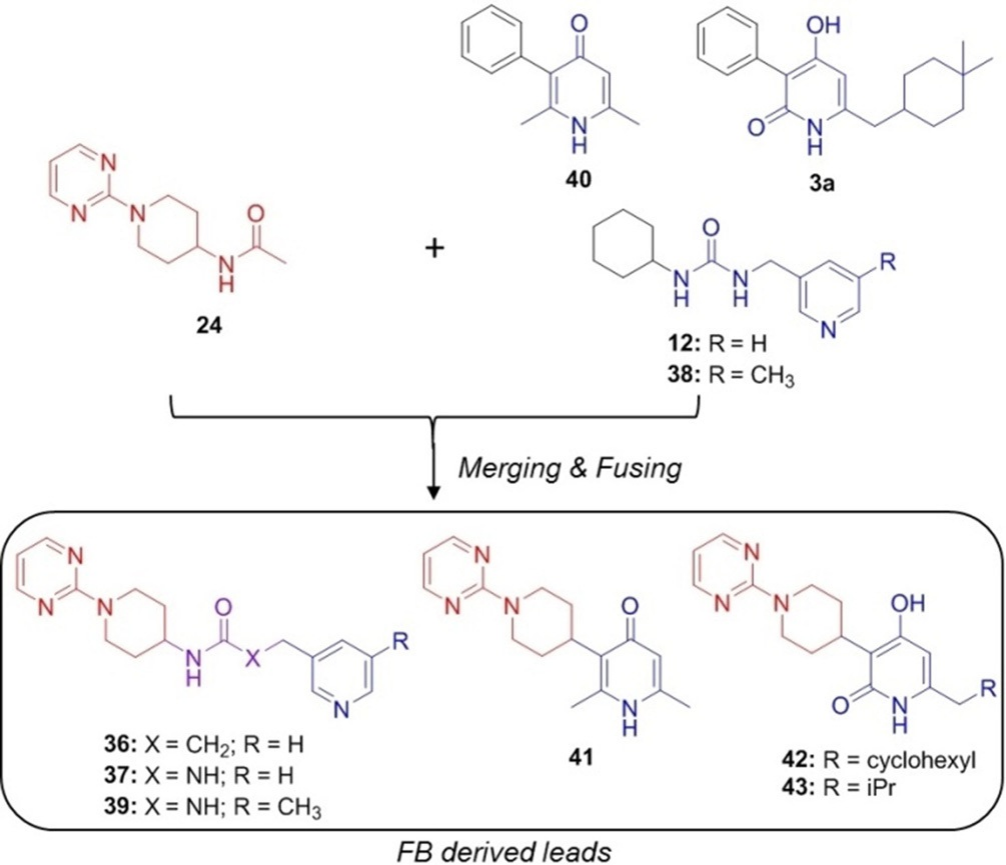
FBLG strategies: merging and growing.

The interaction with Tyr158, previously described for the urea and pyridone derivatives, is not present in the crystal structures obtained for pyrazole fragment hits **4**, **9** (Figure 6 A) and **34** (Figure 6 B), as the Tyr158 side chain forms a water‐mediated bridge with NADH. However, the pyrazole rings occupy the same sub‐pocket, stack against the nicotinamide ring of NADH, and the 2‐*N*‐pyrazole provides an H‐bond interaction with the 20‐OH group of NADH.

**Figure 6:**
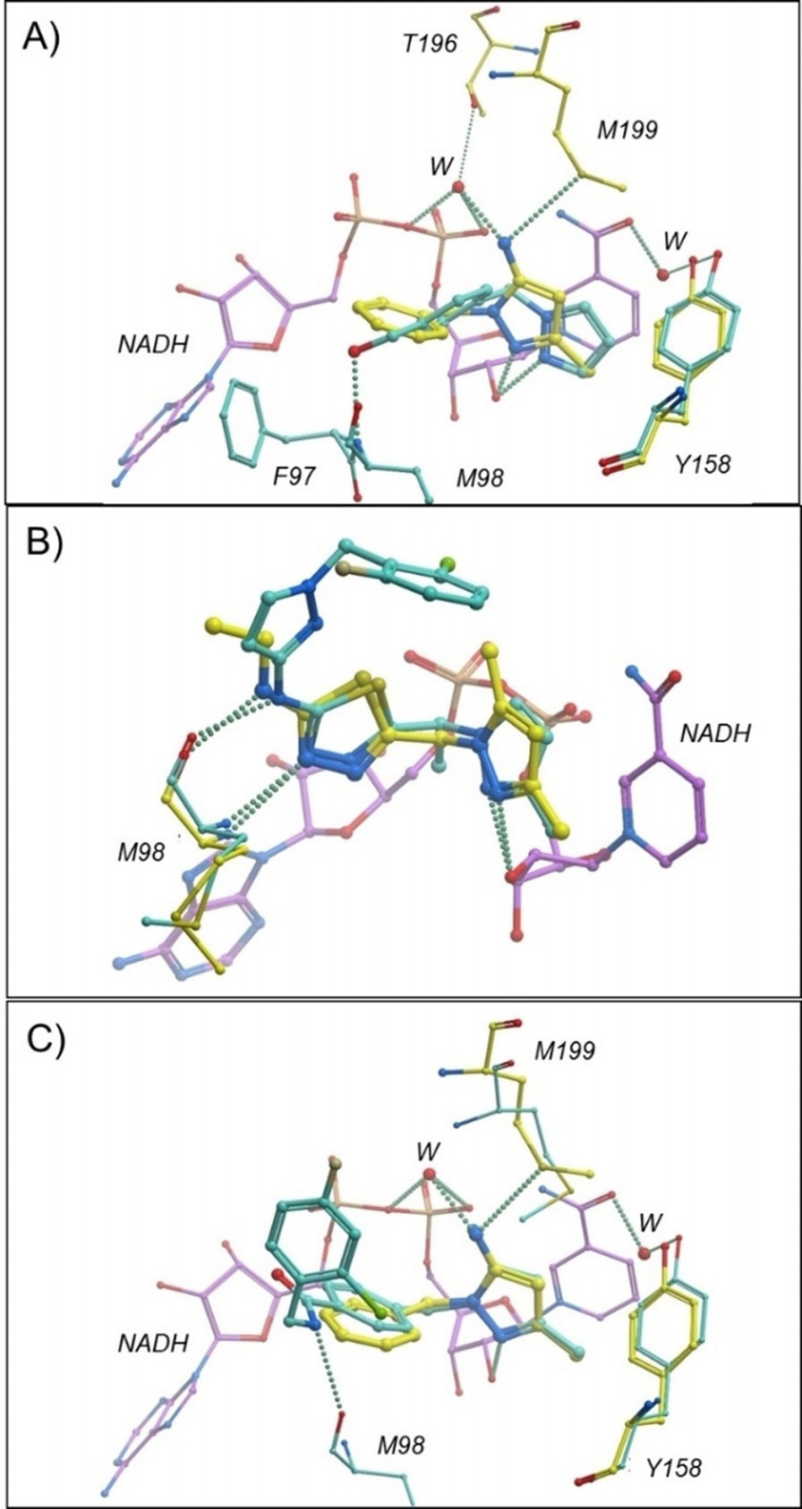
Crystal structures showing novel InhA–NADH–ligand complexes: A) overlay for fragments **4** (blue) and **9** (yellow), B) overlay of **34** (yellow) with the published advanced lead **45** (blue; PDB ID: http://www.rcsb.org/pdb/explore/explore.do?structureId=5JFO), and C) overlay for fragment **9** (yellow) and lead **46** (blue).

Fragment **9** is more potent in the biochemical screen (pIC_50_=3.4) and its 5‐NH_2_ group forms water‐bridged interactions with the NADH phosphate group as well as Met199 and Thr196 (Figure 6 A). In contrast, pyrazole **4** (pIC_50_<3) has a slightly altered binding mode, where the hydroxy group has an additional interaction with Met98 backbone NH (Figure 6 A). In accordance with previous fragment deconstruction studies,^2^, ^3^ the functionally complex InhA informed fragment **34** (pIC_50_<3) retains the binding pose observed for published advanced InhA lead **45** 10 (PDB ID: http://www.rcsb.org/pdb/explore/explore.do?structureId=5JFO, Figure 6 B). The 3N of the thiadiazole ring and NH side group of **34** and **45** H‐bonds with Met98 backbone.

Because good structural overlay (Figure S6) was observed for **9** and the published InhA lead **44** (pIC_50_=6.9, PDB ID: http://www.rcsb.org/pdb/explore/explore.do?structureId=4QXM),^22^ compound **46** (Figure 7 and Scheme S4) was synthesized and showed good InhA activity (pIC_50_=6.3). Introducing the amide FG results in movement of the phenyl group of **9** to allow an additional H‐bond interaction with Met98 backbone CO (Figure 6 C). Similarly, as a result of the deconstructed fragment **34** retaining the binding pose of **45** (pIC_50_=8.1)14c as well as occupying the same pyrazole binding pocket as fragment **9**, compound **47** (Figure 7 and Scheme S5) was synthesized. **47** showed good InhA activity (pIC_50_=7.3) and maintained the pose relative to **45** (Figure S7).

**Figure 7:**
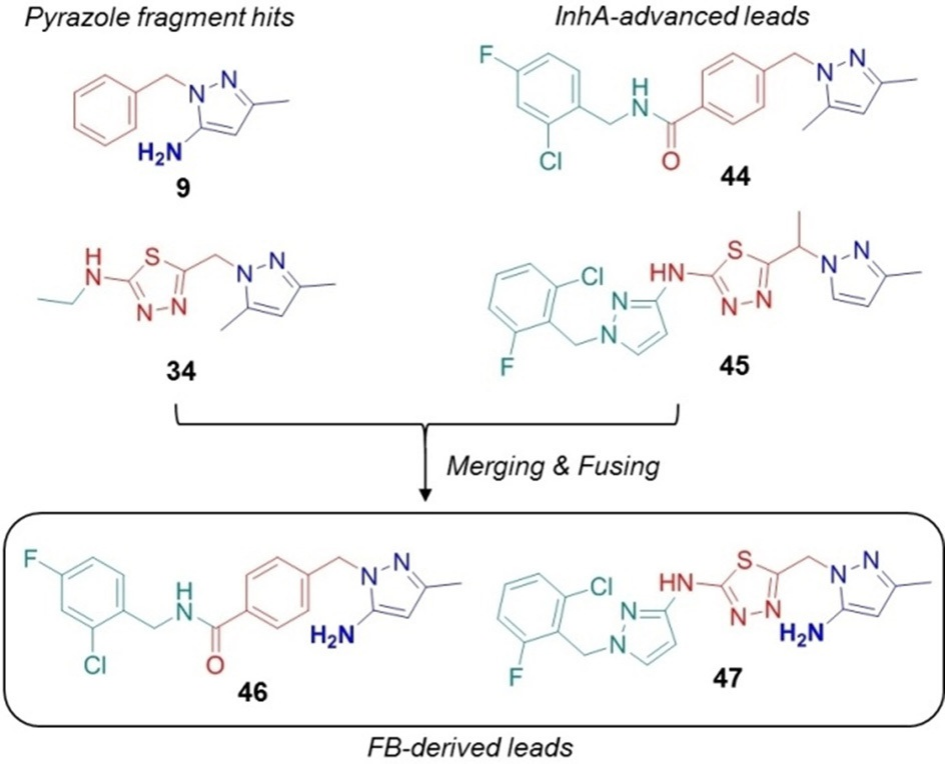
Design strategy for pyrazoles **46** and **47**.

In conclusion, herein we report on the identification of novel InhA fragment hits using STD‐NMR screening, as well as orthogonal InhA biochemical and SPR assays. High hit rates were obtained from screening a recently reported novel fragment set with built FGC versus other fragment sets. Notably, starting from weakly active FGC fragments facilitated rapid fragment based lead generation (FBLG) due to 1) easy chemical tractability and derivatization, 2) retention of the functional group binding pose during fragment evolution through additional interactions with the target, which was confirmed by X‐ray studies. Conversely, elaboration of weakly bound InhA fragments with minimal FGC, relies on FGC implementation, which is likely to alter binding conformation.^23^ This is a common issue associated with fragment optimization, resulting in structure–activity relationship disconnections which are often difficult to interpret. Our findings are also in agreement with the molecular complexity theory by Hann et al.,^3^, ^24^ for which moderately complex ligands, like the identified FGC fragment hits, have a higher probability of a “useful event”, that is the detection of a unique binding pose. These results reported here provide support for the rational design, synthesis and screening of novel diverse fragments with built in functional groups. The described InhA FB‐leads showed good InhA enzymatic activity as well as ligand efficiency (LE) metrics.^25^ Additional optimization efforts have resulted in further improved InhA biochemical as well as Mtb whole cell potency, which will be reported elsewhere.

## Conflict of interest

*The authors declare no conflict of interest*.

## Supporting information

As a service to our authors and readers, this journal provides supporting information supplied by the authors. Such materials are peer reviewed and may be re‐organized for online delivery, but are not copy‐edited or typeset. Technical support issues arising from supporting information (other than missing files) should be addressed to the authors.

Supplementary
