# Supplementary material for: Screening of a Novel Fragment Library with Functional Complexity against Mycobacterium tuberculosis InhA
Source: ChemMedChem. 2018 Feb 19;13(7):672–7. doi: 10.1002/cmdc.201700774 (PMC5915743; doi:10.1002/cmdc.201700774)
Supplement: Supplementary file 1 — Supplementary [file CMDC-13-672-s001.pdf]

CHEMMEDCHEM
Chemistry & Drug Discovery

Supporting Information

Screening of a Novel Fragment Library with Functional Complexity against *Mycobacterium tuberculosis* InhA

Federica Prati,[a, b] Fabio Zuccotto,[a] Daniel Fletcher,[a] Maire A. Convery,[c] Raquel Fernandez-Menendez,[b] Robert Bates,[b] Lourdes Encinas,[b] Jingkun Zeng,[c] Chun-wa Chung,[c] Paco De Dios Anton,[b] Alfonso Mendoza-Losana,[b] Claire Mackenzie,[a] Simon R. Green,[a] Margaret Huggett,[a] David Barros,[b] Paul G. Wyatt,*[a] and Peter C. Ray*[a]

cmdc_201700774_sm_miscellaneous_information.pdf

Author Contributions

F.P. Conceptualization: Equal; Data curation: Supporting; Funding acquisition: Equal; Investigation: Lead; Methodology: Lead; Writing – original draft: Lead; Writing – review & editing: Lead
F.Z. Investigation: Equal; Methodology: Equal; Software: Lead; Visualization: Lead; Writing – original draft: Supporting; Writing – review & editing: Supporting
D.F. Data curation: Equal; Formal analysis: Equal; Investigation: Lead; Methodology: Lead; Validation: Lead; Writing – original draft: Equal; Writing – review & editing: Supporting
M.C. Data curation: Lead; Formal analysis: Lead; Investigation: Supporting; Methodology: Lead; Resources: Equal; Writing – original draft: Equal; Writing – review & editing: Supporting
R.F. Data curation: Equal; Project administration: Supporting; Supervision: Equal; Writing – review & editing: Supporting
R.B. Funding acquisition: Lead; Project administration: Equal; Supervision: Equal; Writing – review & editing: Supporting
L.E. Supervision: Equal; Writing – review & editing: Supporting
J.Z. Data curation: Equal; Formal analysis: Equal; Methodology: Equal; Validation: Equal; Writing – original draft: Supporting; Writing – review & editing: Supporting
C.C. Methodology: Lead; Resources: Lead; Writing – original draft: Supporting; Writing – review & editing: Supporting
P.D. Data curation: Lead; Methodology: Lead; Writing – review & editing: Supporting
A.M. Methodology: Lead; Resources: Supporting; Writing – review & editing: Supporting
C.M. Supervision: Supporting; Writing – review & editing: Supporting
S.G. Data curation: Supporting; Methodology: Supporting; Resources: Supporting; Writing – review & editing: Supporting
M.H. Supervision: Supporting; Writing – review & editing: Supporting; compound handling: Supporting
D.B. Funding acquisition: Equal; Writing – review & editing: Supporting
P.W. Conceptualization: Equal; Funding acquisition: Equal; Writing – review & editing: Supporting
P.R. Conceptualization: Lead; Funding acquisition: Lead; Investigation: Lead; Project administration: Lead; Supervision: Lead; Writing – original draft: Lead; Writing – review & editing: Lead.

Supporting Information

Table of contents **Pg**

Table of contents	Pg
Figure S1	S2
Figure S2	S3
Figure S3	S4
Figure S4	S5
Figure S5	S6
Figure S6	S7
Figure S7	S8
Scheme S1	S9
Scheme S2	S10
Scheme S3	S11
Scheme S4	S12
Scheme S5	S13
Chemistry	S14-S19
STD-^{1}H-NMR screening	S20-S42
InhA enzymatic assay	S43
InhA informed fragment set	S44
Crystallography methods	S45-S51
SPR assay	S52-S53
References	S54

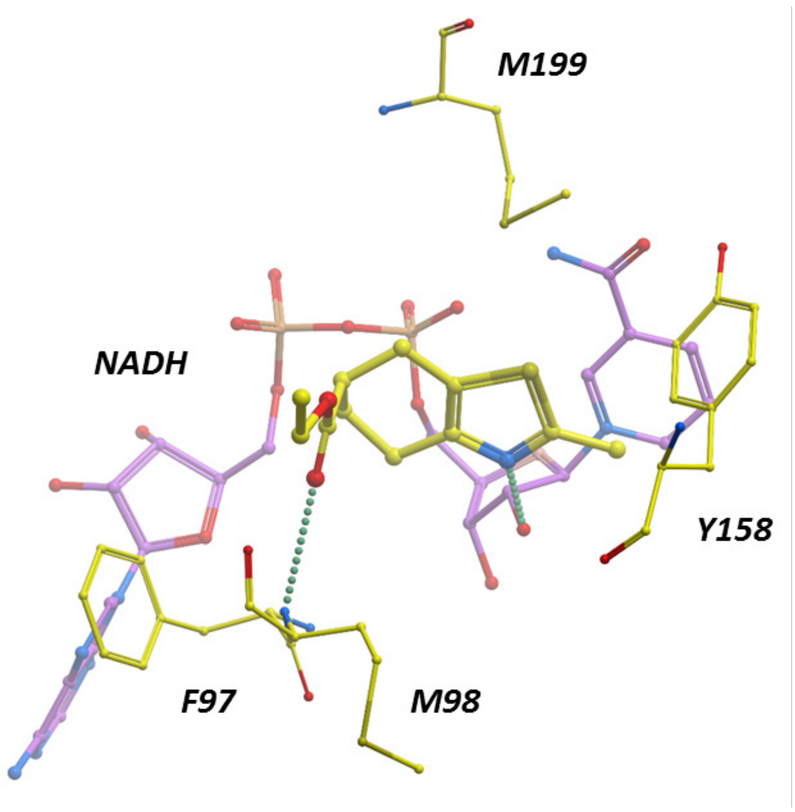

Figure S1. InhA-NADH-ligand crystal structure complex showing fragment **22**.

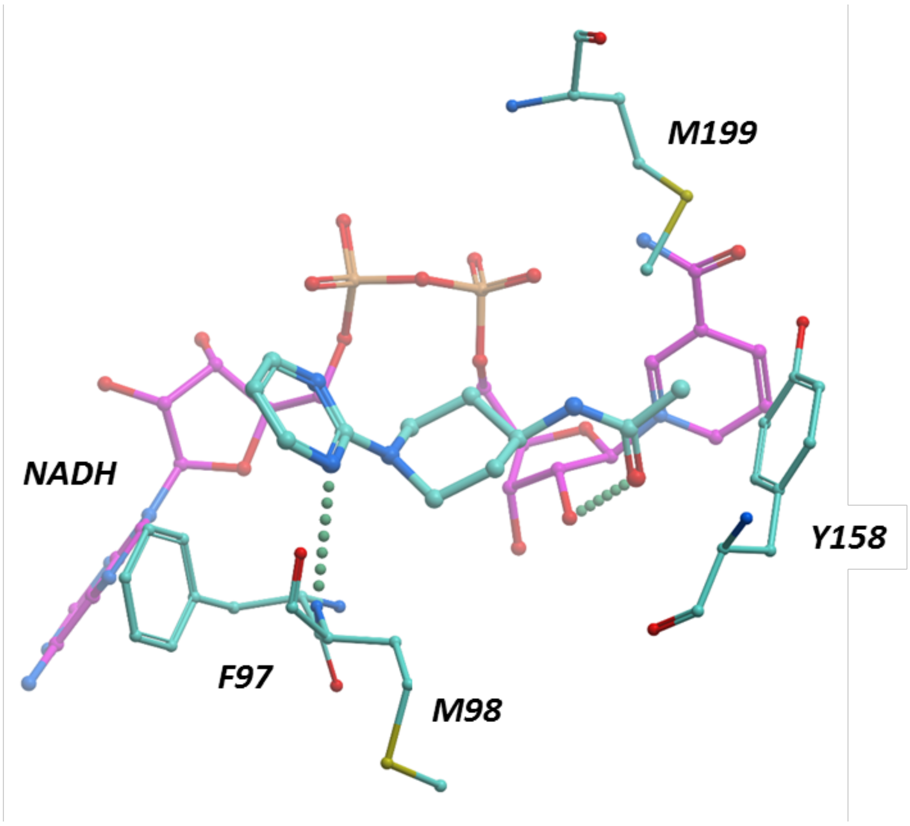

Figure S2. InhA-NADH-ligand crystal structure complex showing fragment **24** bound to binding pockets in chains c and d. No ligand was found in chain a.

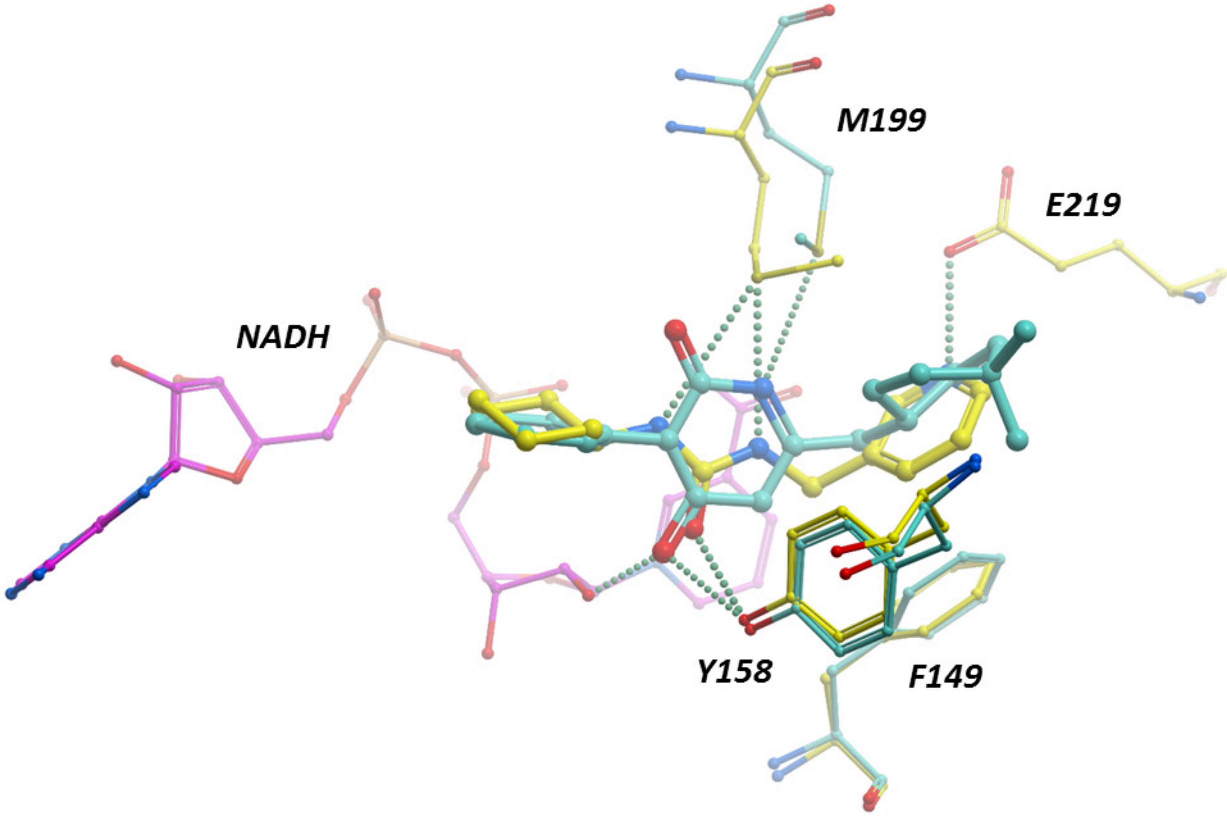

Figure S3. InhA-NADH-ligand crystal structure complex showing overlay for fragment **12**-yellow and advanced lead **3a**-blue.

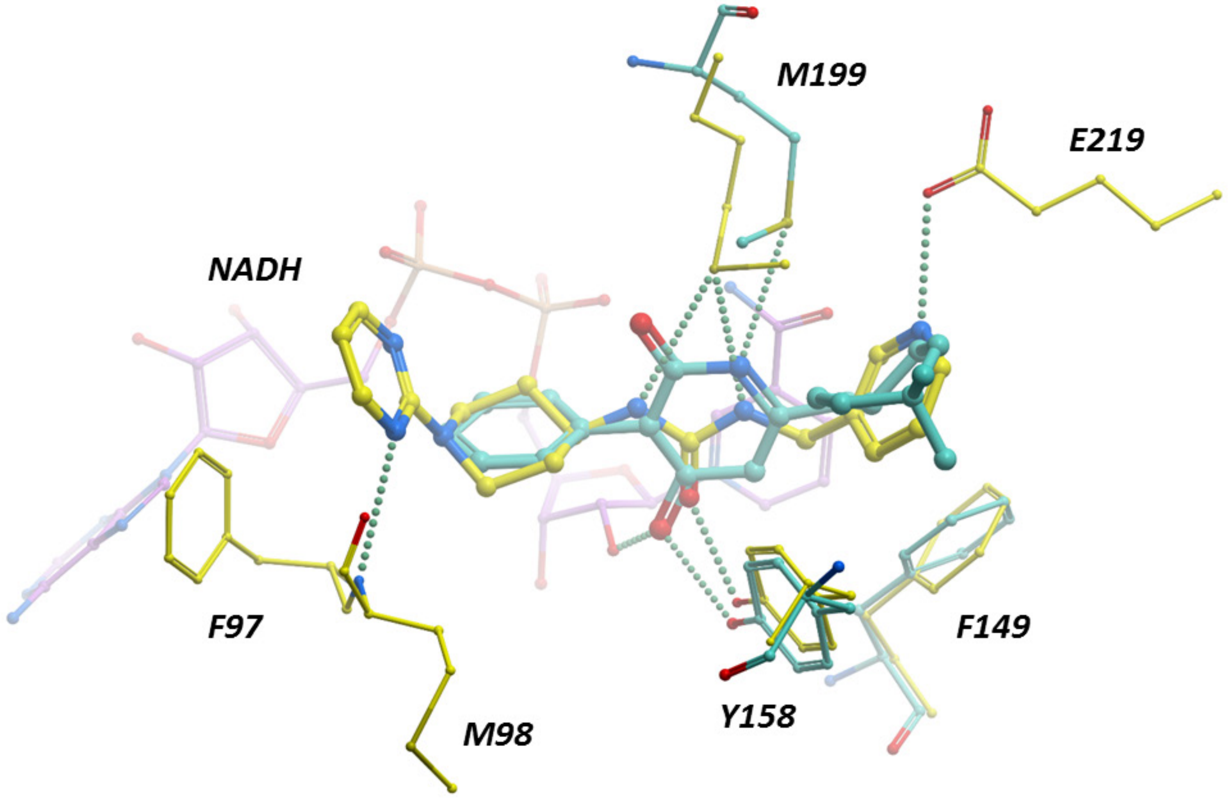

Figure S4. InhA-NADH-ligand crystal structure complex showing overlay for advanced lead **3a**-blue and merged urea lead **37**-yellow.

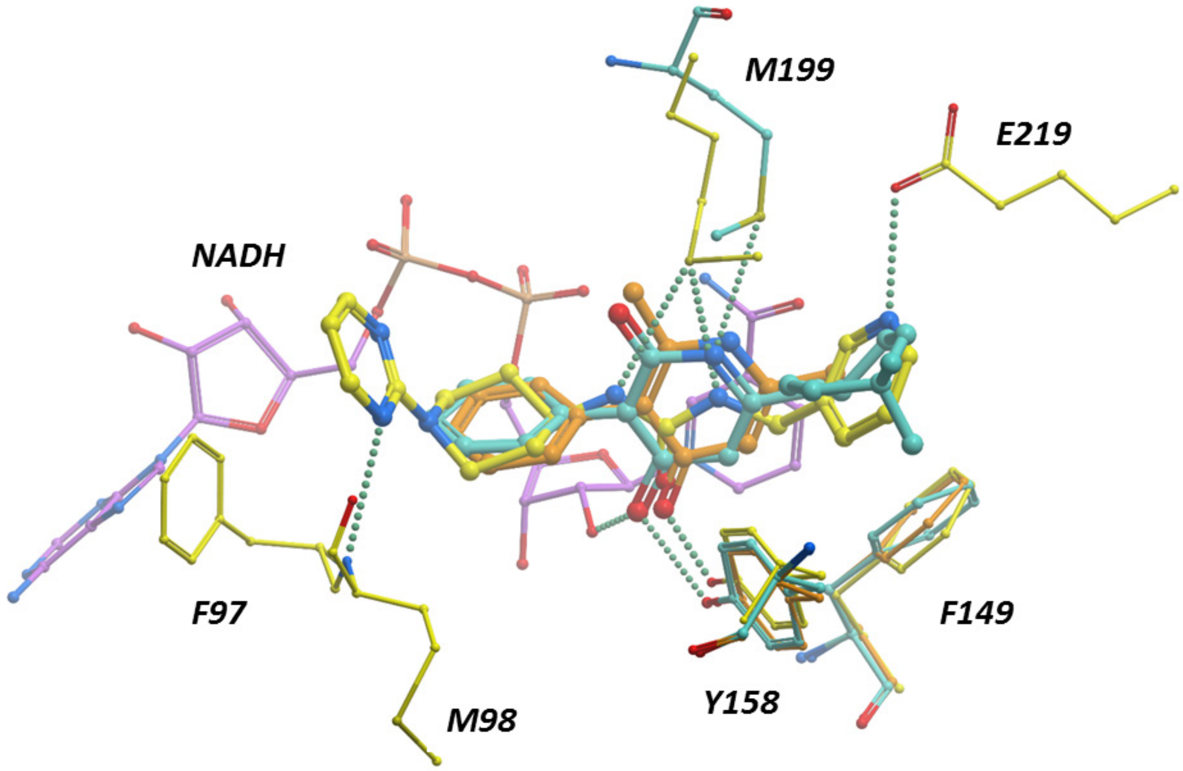

Figure S5. InhA-NADH-ligand crystal structure complex showing overlay for fragment **40**-orange, advanced lead **3a**-blue, and merged urea lead **37**-yellow.

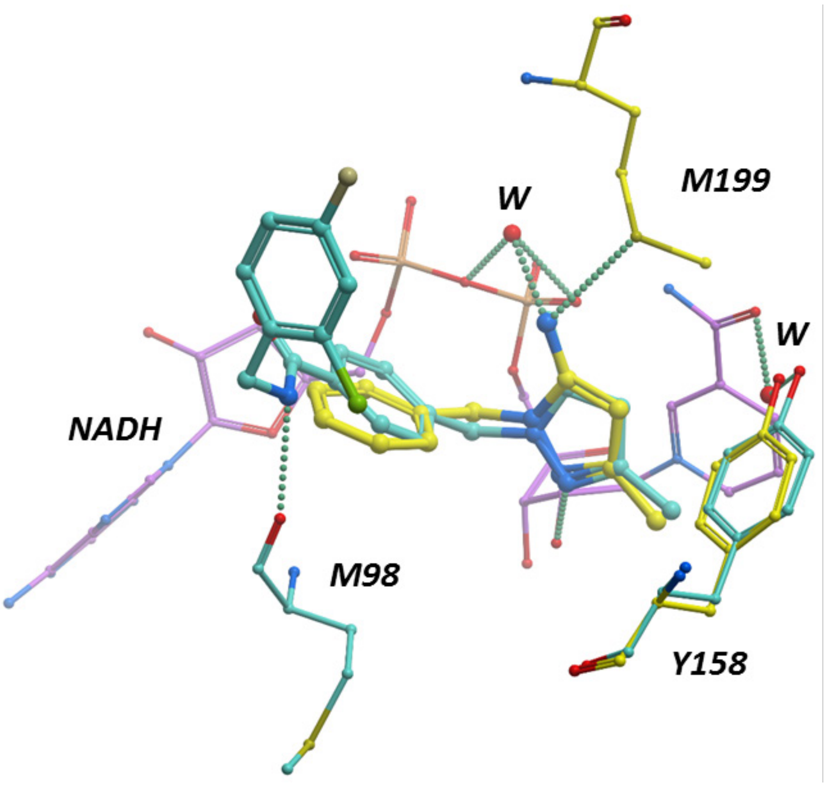

Figure S6. InhA-NADH-ligand crystal structure complex showing overlay for fragment **9**-yellow and advanced lead **44**-blue (PDB 4QXM).

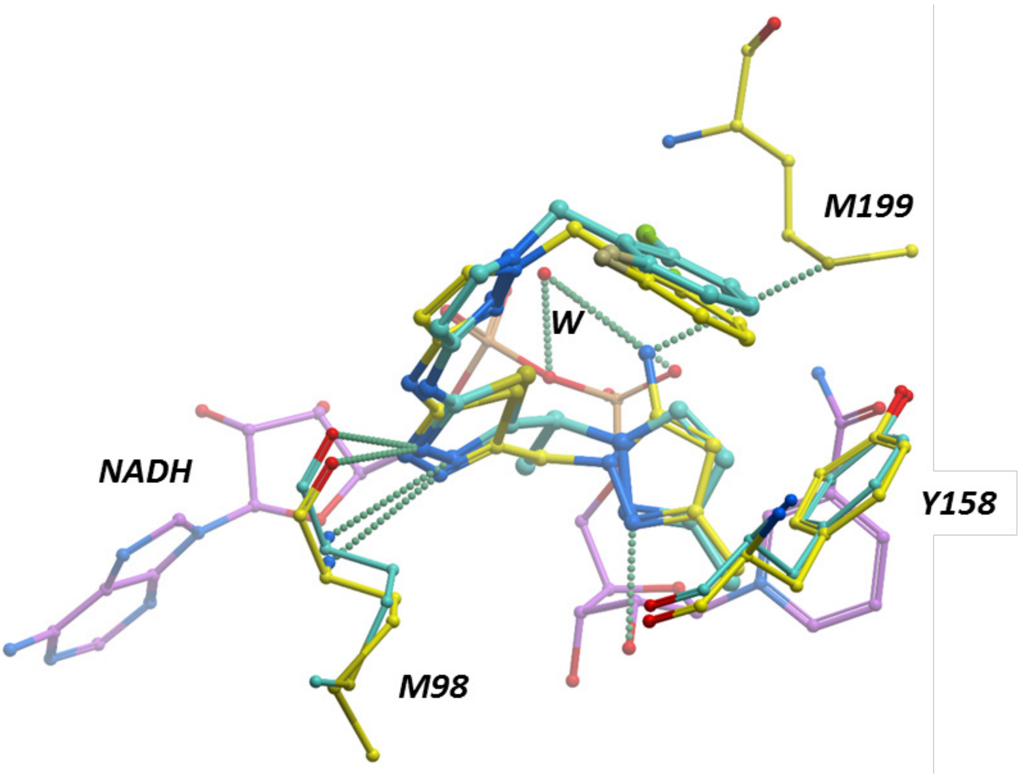

Figure S7. InhA-NADH-ligand crystal structure complex showing overlay for lead **47**-yellow and advanced lead **45**-blue.

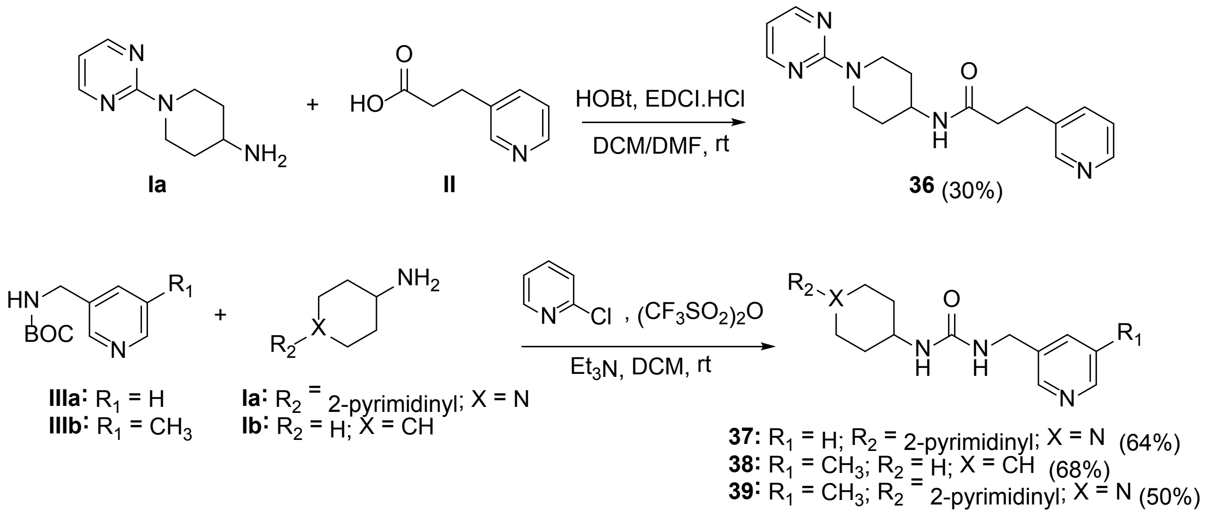

Scheme S1. Ureas **37**-**38** and amide **36** synthetic scheme.

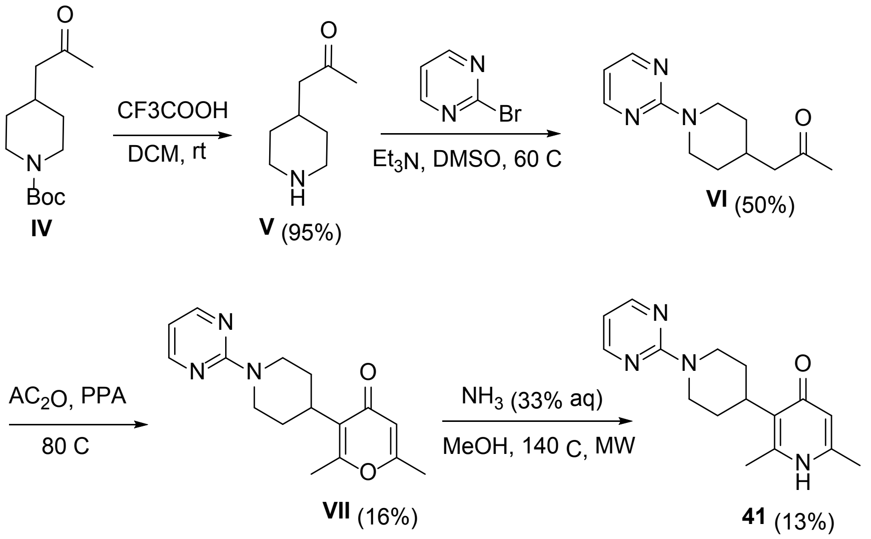

Scheme S2. Pyridone **41** synthetic scheme.

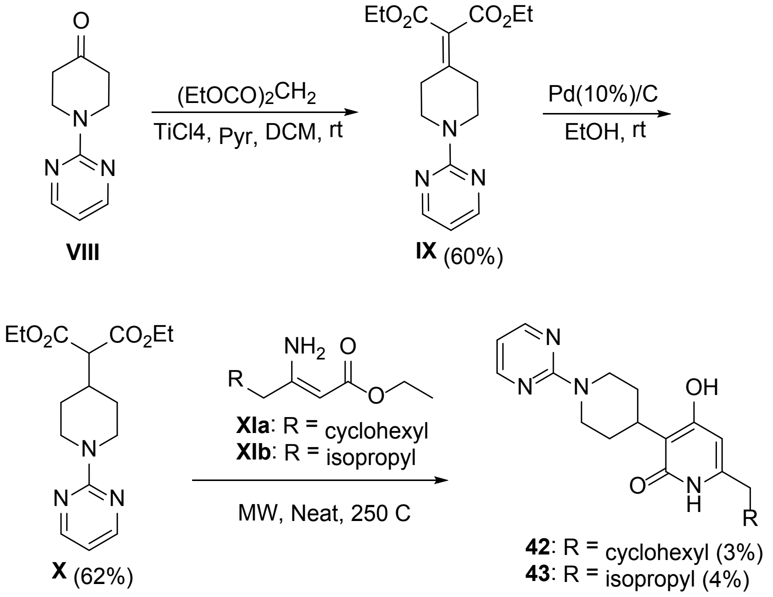

Scheme S3. Pyridones **42-43** synthetic scheme.

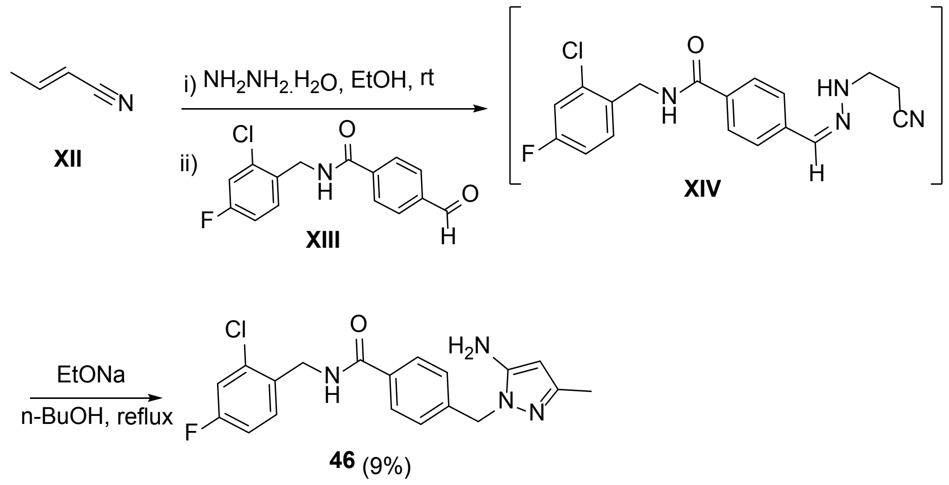

Scheme S4. Pyrazole **46** synthetic scheme.

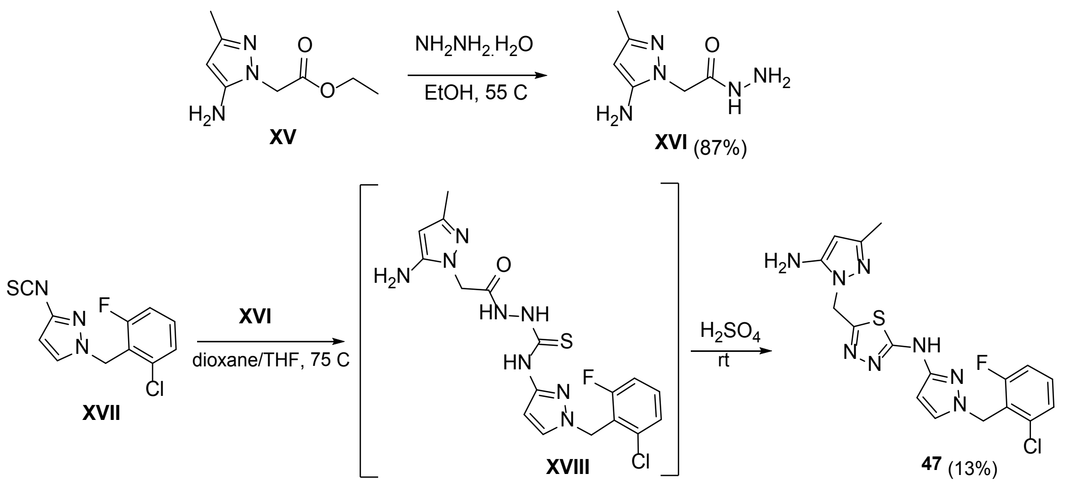

Scheme S5. Pyrazole **47** synthetic scheme.

Chemistry

General: all commercially available reagents and solvents were used without further purification unless otherwise stated.

Reactions were monitored by thin-layer chromatography (TLC) with precoated silica gel 60 F_{254} plates (layer 0.2 mm, Merck). Developed plates were air-dried and analysed under a UV lamp (UV 254/365 nm), or stained with $KMnO_4$ and iodine. Liquid chromatography-mass spectrometry (LC-MS) analysis were performed with an Agilent HPLC 1100 series connected to an Agilent Technologies 6120 quadrupole and to an Agilent diode array detector. LC-MS chromatographic separations were conducted with a Waters XBridge C18 column (50 mm × 4.6 mm, 3.5 µm particle size), using as mobile phase water-ACN mixture with 0.1% HCOH, or water-ACN mixture with 0.1% NH_3HCO_3.

Purification of products was carried out by normal-phase column chromatography using prepacked silica gel cartridge (15-40 µm particle size, Merck). Purifications were performed automatically on a Master-II Flash Biotage instrument.

Reverse-phase high-performance liquid chromatography (HPLC) separations were conducted using either an Agilent HPLC 1100 or 1200 series connected to an Agilent diode array detector, with the column used Waters XBridge C_{18} preparative OBD columns (19 mm × 150 mm, 5 µm particle size or 30 mm × 140 mm, 5 µm particle size). Microwave-assisted chemistry was conducted using a Biotage initiator microwave synthesizer. NMR spectra were recorded on a Bruker DPX-400 and Bruker ARX-500 spectrometers. Measurements were made at rt in the deuterated solvent indicated in each case. Chemical shifts (δ) are reported in parts per million (ppm) relative to the residual solvent peak as internal reference ($CDCl_3$: 7.26 ppm, DMSO-d_6: 2.49 ppm); coupling constants (J) are expressed in hertz (Hz). NMR signals are reported as follows: s (singlet), d (doublet), t (triplet), q (quartet), m (multiplet), and br (broad). LC-MS purity data were collected using a Waters Acquity UPLC instrument coupled with a Waters Acquity single quadrupole mass and photodiode array detectors. LC-MS chromatographic analysis were conducted with a Acquity UPLC BECH C18 column, 50 mm × 3 mm, 1.7 µm particle size; mobile phase, NH_3HCO_3 25 mM + 10% ACN at 6.6 / ACN.

All assay compounds had a measured purity of ≥ 95% as determined using both LC-MS and NMR spectroscopy.

*3-(Pyridin-3-yl)-N-(1-(pyrimidin-2-yl)piperidin-4-yl)propanamide (**36**).* A solution of acid **II** (80 mg, 0.54 mmol), HOBt (70 mg, 0.60 mmol), and EDCI.HCl (113 mg, 0.60 mmol) in DCM/DMF (4 mL, 1:1) was stirred at rt for 2h. Amine **Ia** (88 mg, 0.49 mmol) was added, and the resulting mixture was stirred overnight at rt. After diluting with EtOAc (30 mL), the reaction mixture was washed with 5% aqueous LiCl solution (20 mL). The organic phase was dried over anhydrous Na_2SO_4, filtered, and evaporated in vacuo. The crude material was purified by reverse phase HPLC, eluting with 20-100% ACN in 0.1% aqueous NH_4HCO_3 solution. This yielded **36** as a white solid: 45 mg (30%). ^{1}H NMR (DMSO-d_6, 400MHz): δ = 8.42 (d, *J*=1.8 Hz, 1H), 8.39 (dd, *J*=4.8, 1.8 Hz, 1H), 8.34 (d, *J*=4.8 Hz, 2H), 7.78 (d, *J*=7.6 Hz, 1H), 7.59-7.62 (m, 1H), 7.25-7.34 (m, 1H), 6.59 (t, *J*=4.7 Hz, 1H), 4.39-4.51 (m, 2H), 3.76-3.89 (m, 1H), 2.97-3.11 (m, 2H), 2.83 (t, *J*=7.5 Hz, 2H), 2.38 (t, *J*=7.6 Hz, 2H), 1.69-1.73 (m, 2H), 1.13-1.28 ppm

(m, 2H); ^{13}C NMR (DMSO-d$_6$, 100MHz): δ = 170.7, 161.6, 158.4, 150.0, 147.7, 137.1, 136.3, 123.8, 110.3, 46.4, 42.6, 37.0, 31.5, 28.7 ppm; MS (ESI) m/z 312 $[M + H]^+$.

General procedure[1] for the synthesis of ureas ***37***-***39***:

To a solution of the proper *N*-BOC protected amine **III** (1.0 eq.) in dry DCM (0.025 M), 2-Cl-pyrdine (3.0 eq.) was added, followed by $(CF_3SO_2)_2O$ (1.5 eq.). After stirring at rt for 50 min, Et_3N (6.0 eq.) and the amine of interest **IV** (6.0 eq.) were added, and the resulting mixture was stirred overnight at rt. After washing with water, the organic phase was dried over anhydrous Na_2SO_4, filtered, and evaporated in vacuo. The crude material was purified by reverse phase HPLC to yield the compound of interest.

*1-(Pyridin-3-ylmethyl)-3-(1-(pyrimidin-2-yl)piperidin-4-yl)urea (**37**).* The title compound was obtained according to the general procedure using tert-butyl (pyridin-3-ylmethyl)carbamate **IIIa** (52 mg, 0.25 mmol) and 1-(pyrimidin-2-yl)piperidin-4-amine **Ia** (267 mg, 1.5 mmol). The crude material was purified by reverse phase HPLC, eluting with 5-95% ACN in 0.1% aqueous NH_4HCO_3 solution. This yielded **37** as a white solid: 50 mg (64%). ^{1}H NMR (DMSO-d$_6$, 500 MHz): δ = 8.47 (d, *J*=1.6 Hz, 1H), 8.43 (dd, *J*=4.8, 1.6 Hz, 1H), 8.33 (d, *J*=4.7 Hz, 1H), 7.64-7.66 (m, 1H), 7.34 (dd, *J*=7.8, 0.8 Hz, 1H), 6.59 (t, *J*=4.8 Hz, 1H), 6.32 (t, *J*=6.0 Hz, 1H) 6.03 (d, *J*=7.8 Hz, 1H), 4.44-4.83 (m, 2H), 4.23 (d, *J*=6.0 Hz, 1H), 3.65-3.72 (m, 1H), 3.05-3.10 (m, 2H), 1.79-1.83 (m, 2H), 1.22-1.30 ppm (m, 2H); ^{13}C NMR ($CDCl_3$, 100 MHz): δ = 161.3, 158.0, 157.8, 148.1, 147.8, 136.0, 135.9, 123.7, 109.6, 47.3, 42.7, 41.3, 32.4 ppm; MS (ESI) m/z 313 $[M + H]^+$.

*1-Cyclohexyl-3-((5-methylpyridin-3-yl)methyl)urea (**38**)*. The title compound was obtained according to the general procedure using tert-butyl ((5-methylpyridin-3-yl)methyl)carbamate **IIIb** (155 mg, 0.70 mmol) and cyclohexanamine **Ib** (416 mg, 4.2 mmol). The crude material was purified by reverse phase HPLC, eluting with 5-95% ACN in 0.1% aqueous NH_4HCO_3 solution. This yielded **38** as a white solid: 118 mg (68%). ^{1}H NMR ($CDCl_3$, 500 MHz): δ = 8.36 (s, 2H), 7.50 (s, 1H), 4.53 (br. s, 1H), 4.39 (d, *J*=5.8 Hz, 2H), 4.20 (d, *J*=6.8 Hz, 1H), 3.52-3.67 (m, 1H), 2.35 (s, 3H), 1.95-1.98 (m, 2H), 1.70-1.74 (m, 2H), 1.60-1.65 (m, 1H), 1.33-1.44 (m, 2H), 1.09-1.21 ppm (m, 3H); ^{13}C NMR (DMSO-d$_6$, 100 MHz): δ = 157.8, 148.6, 146.3, 136.3, 135.7, 132.8, 48.4, 40.9, 33.7, 25.8, 25.0, 18.3; MS (ESI) m/z 248 $[M + H]^+$; HRMS (ESI) m/z: calculated for $C_{14}H_{22}N_3O^+$ $[M + H]^+$ 248.1757, found 248.1758.

*1-((5-Methylpyridin-3-yl)methyl)-3-(1-(pyrimidin-2-yl)piperidin-4-yl)urea (**39**).* The title compound was obtained according to the general procedure using tert-butyl ((5-methylpyridin-3-yl)methyl)carbamate **IIIb** (70 mg, 0.31 mmol) and 1-(pyrimidin-2-yl)piperidin-4-amine **Ia** (337 mg, 1.86 mmol). The crude material was purified by reverse phase HPLC, eluting with 5-95% ACN in 0.1% aqueous NH_4HCO_3 solution. This yielded **37** as a white solid: 50 mg (50%). ^{1}H NMR (DMSO-d$_6$, 400 MHz): δ = 8.34 (d, *J*=4.8 Hz, 2H), 8.22-8.31 (m, 2H), 7.45 (s, 1H), 6.58 (t, *J*=4.8 Hz, 1H), 6.28 (t, *J*=5.9 Hz, 1H), 6.01 (d, *J*=7.8 Hz, 1H), 4.39-4.51 (m, 2H), 4.19 (d, *J*=6.1 Hz, 2H), 3.66-3.69 (m, 1H), 3.03-3.10 (m, 2H), 2.28 (s, 3H), 1.79-1.83 (m, 2H), 1.18-1.32 ppm (m, 2H); ^{13}C NMR ($CDCl_3$, 100 MHz): δ = 161.5, 157.8, 157.3, 149.2, 146.0, 136.2, 134.6, 133.3, 109.7, 48.0, 42.7, 41.8, 32.7, 18.4 ppm; MS (ESI) m/z 327 $[M + H]^+$.

*1-(Piperidin-4-yl)propan-2-one (**V**)*. To a solution of *N*-BOC protected amine **IV** (580 mg, 2.43 mmol) in DCM (12 mL), TFA (2.40 mL, 31.6 mmol) was added. After stirring at rt overnight, the solvent was removed under reduced pressure. The residue was taken up with MeOH, and purified on a 5g isolute SCX-2 cartridge, eluting with 2N methanolic NH_3 solution. This yielded **V** as a colourless oil (330 mg, 95%), which was used in the next step without further purification. ^{1}H NMR ($CDCl_3$, 400 MHz): δ = 3.12 (d, *J*=11.9 Hz, 2H), 2.70 (td, *J*=12.3, 2.4 Hz, 2H), 2.40 (d, *J*=6.8 Hz, 2H), 2.8 (s, 3H), 1.97-2.02 (m, 1H), 1.73 (d, *J*=13.9 Hz, 2H), 1.16-1.26 ppm (m, 2H); MS (ESI) m/z 142 $[M + H]^+$.

1-*(1-(Pyrimidin-2-yl)piperidin-4-yl)propan-2-one (**VI**)*. Amine **V** (330 mg, 2.34 mmol), 2-bromopyrimidine (370 mg, 2.34 mmol), and Et_3N (590 mg, 5.84 mmol) were mixed together in DMSO (1.0 ml), and stirred overnight at 60 °C. After cooling at rt, the solvent was removed under reduced pressure. The residue was taken up with EtOAc (30 mL) and washed with water (30 mL). The organic phase was dried over Na_2SO_4 anhydrous, filtered, and evaporated in vacuo. The crude material was purified by normal-phase flash chromatography, eluting with 0-30% EtOAc in cyclohexane. This yielded **VI** as a white solid: 246 mg (50%). ^{1}H NMR ($CDCl_3$, 400 MHz): δ = 8.32 (d, *J*=4.8 Hz, 2H), 6.47 (t, *J*=4.8 Hz, 1H), 4.76 (d, *J*=13.4 Hz, 2H), 2.91-2.98 (m, 2H), 2.43 (d, *J*=6.8 Hz, 2H), 2.18 (s, 3H), 2.14-2.16 (m, 1H), 1.81 (d, *J*=12.6 Hz, 2H), 1.17-1.27 ppm (m, 2H); MS (ESI) m/z 220 $[M + H]^+$.

*2,6-Dimethyl-3-(1-(pyrimidin-2-yl)piperidin-4-yl)-4H-pyran-4-one (**VII**).* A solution of ketone **VI** (246 mg, 1.12 mmol) in acetic anhydride (1.0 mL) was added dropwise to a vigorously stirred solution of polyphosphoric acid (1.95 g, 8.12 mmol) in acetic anhydride (1.0 mL), and heated at 80 °C for 30 min. After cooling to rt, the reaction mixture was poured into water (30 mL), neutralized with Na_2CO_3 and extracted with DCM (4 x 30 mL). The collected organic layers were dried over Na_2SO_4 anhydrous, filtered, and evaporated in vacuo. The crude material was purified by normal-phase flash chromatography, eluting with 0-40% EtOAC/EtOH (3:1) in cyclohexane. This yielded **VII** as an orange oil: 50 mg (16%). ^{1}H NMR ($CDCl_3$, 400 MHz): δ = 8.32 (d, *J*=4.5 Hz, 2H), 6.47 (t, *J*=4.8 Hz, 1H), 6.05 (s, 1H), 4.89-4.93 (m, 2H), 3.04-3.10 (m 1H), 2.89-2.96 (m, 2H), 2.34 (s, 3H), 2.23-2.30 (m, 2H), 2.21 (s, 3H), 1.61 ppm (d, *J*=10.4 Hz, 2H); MS (ESI) m/z 286 $[M + H]^+$.

*2,6-Dimethyl-3-(1-(pyrimidin-2-yl)piperidin-4-yl)pyridin-4(1H)-one (**41**).* To a solution of pyranone **VII** (45 mg, 0.16 mmol) in MeOH (0.5 mL), 30% aqueous NH_3 solution was added (2 mL), and the resulting mixture was heated in a MW reactor at 140°C for 9h. The solvent was removed in vacuo, and the crude material was purified by reverse phase HPLC, eluting with 10-100% ACN in 0.1% aqueous NH_4HCO_3 solution. This yielded **41** as a yellow solid: 6 mg (13%). ^{1}H NMR (DMSO-d_6, 400 MHz): δ = 10.73 (br. s., 1H), 8.32 (d, *J*=4.8 Hz, 2H), 6.55 (t, *J*=4.7 Hz, 1H), 5.71 (s, 1H), 4.76 (d, *J*=12.9 Hz, 2H), 2.75-2.84 (m, 3H), 2.28-2.45 (m, 2H), 2.24 (s, 3H), 2.10 (s, 3H), 1.33 ppm (d, *J*=12.1 Hz, 2H); ^{13}C NMR (DMSO-d_6, 100 MHz): δ = 187.2, 161.5, 158.3, 145.5, 143.6, 126.8, 115.1, 109.7, 44.6, 36.6, 27.9, 18.5, 17.6; MS (ESI) m/z 285 $[M + H]^+$.

*Diethyl 2-(1-(pyrimidin-2-yl)piperidin-4-ylidene)malonate (**IX**).* Diethylmalonate (620 mg, 3.88 mmol) and ketone **VIII** (688 mg, 3.88 mmol) were added to a solution of $TiCl_4$ in DCM (1.26 mL, 1.0 M) at 0°C. After stirring at the same temperature for 40 min, pyridine was added (1.23g, 15.53 mmol) and the resulting mixture was stirred at rt for 4 h. The reaction mixture was diluted with saturated aqueous NH_4Cl solution (40 mL), and extracted with DCM (3 x 30 mL). The collected organic layers were dried over Na_2SO_4 anhydrous, filtered, and evaporated in vacuo. The crude material was purified by normal-phase flash chromatography, eluting with 0-30% EtOAC in cyclohexane. This yielded **IX** as a colourless oil: 740 mg (60%).^{1}H NMR ($CDCl_3$, 400 MHz): δ = 8.33 (d, *J*=4.8 Hz, 2H), 6.52 (t, *J*=4.8 Hz, 1H), 4.27 (q, *J*=7.1 Hz, 4H), 3.91-3.99 (m, 4H), 2.72-2.79 (m, 4H), 1.32 ppm (t, *J*=7.2 Hz, 6H); MS (ESI) m/z 320 [M + H]$^+$.

*Diethyl 2-(1-(pyrimidin-2-yl)piperidin-4-yl)malonate (**X**).* To a solution of diethyl 2-methylenemalonate **IX** (740 mg, 2.32 mmol) in EtOH (26 mL), 10% Pd/C (246 mg, 0.23 mmol) was added under an inert N_2 atmosphere. The reaction mixture was stirred at rt overnight with a H_2 balloon. The reaction mixture was filtered on a syringe filter, and the filtrate was concentrated in vacuo. The crude material was purified by normal-phase flash chromatography, eluting with 0-5% MeOH in DCM. This yielded **X** as a colourless oil: 458 mg (62%). ^{1}H NMR ($CDCl_3$, 400 MHz): δ = 8.29 (d, *J*=4.8 Hz, 2H), 6.45 (t, *J*=4.7 Hz, 1H), 4.78 (d, *J*=13.6 Hz, 2H), 4.21 (q, *J*=7.2 Hz, 4H), 3.19 (d, *J*=9.1 Hz, 1H), 2.87-2.94 (m, 2H), 2.32-2.45 (m, 1H), 1.81 (d, *J*=12.9 Hz, 2H), 1.34-1.49 (m, 2H), 1.27 (t, *J*=7.2 Hz, 6H), ppm; MS (ESI) m/z 322 [M + H]$^+$.

*General procedure[2] for the synthesis of pyridones **42** and **43**:*
Malonate **X** (1 eq.) and the proper ethyl-3-amino-alkyl-2-enoates **IV**[2b] (1 eq.) were mixed in a MW vial and heated in MW reactor at 250 °C for 30 min. The crude material was purified by reverse phase HPLC chromatography and triturated with cyclohexane to yield the compound of interest.

*6-(Cyclohexylmethyl)-4-hydroxy-3-(1-(pyrimidin-2-yl)piperidin-4-yl)pyridin-2(1H)-one (**42**).* The title compound was obtained according to the general procedure using malonate **X** (445 mg, 1.39 mmol) and ethyl-3-amino-4-cyclohexylbut-2-enoate **XIa** (293 mg, 1.39 mmol). The crude material was purified by reverse phase HPLC, eluting with 30-80% ACN in 0.1% aqueous NH_4HCO_3 solution. This yielded **42** as a white solid: 15 mg (3%). ^{1}H NMR (DMSO-d_6, 400 MHz): δ = 10.74 (br. s., 1H), 9.95 (br. s., 1H), 8.32 (d, *J*=4.8 Hz, 2H), 6.54 (t, *J*=4.7 Hz, 1H), 5.60 (s, 1H), 4.76 (d, *J*=11.6 Hz, 2H), 3.12-3.18 (m, 1H), 2.70-2.85 (m, 2H), 2.10-2.27 (m, 4H), 1.43-1.73 (m, 6H), 1.28-1.43 (m, 2H), 1.01-1.25 (m, 3H), 0.80-0.96 ppm (m, 2H); ^{13}C NMR ($CDCl_3$, 100 MHz): δ = 169.3, 169.0, 165.2, 161.6, 149.1, 115.7, 112.8, 104.5, 48.9, 44.7, 41.3, 37.1, 36.8, 32.1, 30.1, 29.9 ppm; MS (ESI) m/z 367 [M + H]$^+$.

*4-Hydroxy-6-isobutyl-3-(1-(pyrimidin-2-yl)piperidin-4-yl)pyridin-2(1H)-one (**43**).* The title compound was obtained according to the general procedure using malonate **X** (330 mg, 1.03 mmol) and ethyl-3-amino-5-methylhex-2-enoate **XIb** (176 mg, 1.03 mmol). The crude material was purified by reverse phase HPLC, eluting with 20-80% ACN in 0.1% aqueous NH_4HCO_3 solution. This yielded **43** as a white solid:

12 mg (4%). ^{1}H NMR (DMSO-d$_6$, 400 MHz): δ = 10.79 (s, 1H), 9.93 (s, 1H), 8.32 (d, *J*=4.8 Hz, 2H), 6.54 (t, *J*=4.8 Hz, 1H), 5.62 (s, 1H), 4.76 (d, *J*=12.9 Hz, 2H), 3.10-3.21 (m, 1H), 2.73-2.85 (m, 2H), 2.13-2.26 (m, 4H), 1.82-1.88 (m, 1H), 1.36 (d, *J*=10.6 Hz, 2H), 0.84 ppm (d, *J*=6.4 Hz, 6H); ^{13}C NMR (CDCl$_3$, 100 MHz): δ = 169.3, 168.9, 165.2, 161.6, 149.4, 115.8, 112.8, 104.4, 48.9, 46.0, 37.1, 32.1, 32.0, 26.0 ppm; MS (ESI) m/z 338 [M + H]$^+$.

*4-((5-Amino-3-methyl-1H-pyrazol-1-yl)methyl)-N-(2-chloro-4-fluorobenzyl)benzamide (**46**)*. To an iced cold solution of crononitrile **XII** (169 mg, 2.52 mmol) in EtOH (2.5 mL), hydrazine hydrate (120 mg, 2.40 mmol) was added dropwise, and the resulting mixture was stirred overnight at rt. After cooling to 0 °C, benzaldehyde **XIII**[3] (727 mg, 2.49 mmol) was added portion wise and the resulting mixture was stirred 4h at rt to afford intermediate **XIV**.

The solvent was removed in vacuo, and the residue was taken up with dry n-BuOH (2 mL) and added to a solution of EtONa (342 mg, 5.03 mmol) in EtOH (2mL). After heating at reflux for 1h, the solvent was removed under reduced pressure, and the crude material was purified by normal-phase flash chromatography, eluting with 0-40% EtOAc/EtOH (3:1) in cyclohexane. The obtained solid was further purified by reverse phase HPLC, eluting with 30-100% ACN in 0.1% aqueous NH_4HCO_3 solution. This yielded **46** as a white solid: 80 mg (9%). ^{1}H NMR (DMSO-d$_6$, 400 MHz): δ = 9.00 (t, *J*=5.8 Hz, 1H), 7.84 (d, *J*=8.3 Hz, 2H), 7.46 (dd, *J*=8.8, 2.5 Hz, 1H), 7.38 (dd, *J*=8.8, 6.3 Hz, 1H), 7.19-7.24 (m, 3H), 5.20 (s, 2H), 5.12 (s, 1H), 5.07 (s, 2H), 4.50 (d, *J*=5.8 Hz, 2H), 1.97 ppm (s, 3H); ^{13}C NMR (DMSO-d$_6$, 100 MHz): δ = 166.7, 161.4 (d, *J*=246.8 Hz) 148.0, 146.6, 142.4, 133.3 (d, *J*=3.7 Hz), 133.2, 133.1 (d, *J*=11 Hz), 130.5 (d, *J*=10 Hz), 127.8, 127.5, 116.8 (d, *J*=25 Hz), 114.7 (d, *J*=20 Hz), 88.4, 49.6, 40.5, 14.3 ppm; MS (ESI) m/z 373 [M + H]$^+$.

*2-(5-Amino-3-methyl-1H-pyrazol-1-yl)acetohydrazide (**XVI**).* To a stirred solution of hydrazine hydrate (164 mg, 3.27 mmol) in EtOH (0.8 mL), a solution of ester **XV** (100 mg, 0.54 mmol) in EtOH (0.8 mL) was added dropwise, and the resulting mixture was heated at 55 °C overnight. The solvent was removed in vacuo and the residue was triturated with water. The resulting white solid was collected by filtration and dried under vacuum. This yielded **XVI** as a white solid (80 mg, 87%), which was used in the next step without further purification. ^{1}H NMR (DMSO-d$_6$, 400 MHz): δ = 9.03 (br. s., 1H), 5.10 (s, 1H), 5.08 (s, 2H), 4.37 (s, 2H), 4.27 (d, *J*=4.0 Hz, 2H), 1.95 ppm (s, 3H); MS (ESI) m/z 170 [M + H]$^+$.

*5-((5-Amino-3-methyl-1H-pyrazol-1-yl)methyl)-N-(1-(2-chloro-6-fluorobenzyl)-1H-pyrazol-3-yl)-1,3,4-thiadiazol-2-amine (**47**).* A suspension of hydrazine **XVI** (80 mg, 0.47 mmol) and isothiacyanate **XVII**[4] (126 mg, 0.47 mmol) in dioxane/THF (8 mL, 1:1) was heated at 75 °C for 8h. The organic solvent was evaporated in vacuo to give the intermediate hydrazinecarbothiamide **XVIII** as a yellow solid. The crude material was dissolved in H_2SO_4 (3 mL) and stirred at rt for 2h. After cooling to 0 °C, the reaction mixture was made basic by adding 33% aqueous NH_3 solution. The resulting yellow precipitate was collected by filtration and purified by reverse phase HPLC, eluting with 30-80% ACN in 0.1% aqueous NH_4HCO_3 solution. This yielded **47** as a white solid: 25 mg (13%). ^{1}H NMR (DMSO-d$_6$, 400 MHz): δ = 10.81 (br. s., 1H), 7.71 (d, *J*=2.3 Hz, 1H), 7.42-7.46 (m, 1H), 7.36 (d, *J*=8.1 Hz, 1H), 7.21-7.31 (m, 1H), 5.95 (d,

J=2.3 Hz, 1H), 5.33 (s, 2H), 5.29 (s, 2H), 5.25 (s, 2H), 5.13 (s, 1H), 2.00 ppm (s, 3H); ^{13}C NMR (DMSO-d_6, 100 MHz): δ = 164.5, 161.9 (d, J=248.0 Hz), 157.9, 154.2, 148.1, 147.4, 135.5 (d, J=5.1 Hz), 132.6, 131.6 (d, J=9.5 Hz), 126.1 (d, J=3.7 Hz), 122.5 (d, J=17.6 Hz), 115.2 (d, J=21.9 Hz), 93.9, 88.6, 46.3, 45.7, 14.4 ppm; MS (ESI) m/z 419 $[M + H]^+$.

STD-^{1}H-NMR screening

All data was acquired using a Bruker AVANCE NMR spectrometer operating at 500.13 MHz equipped with a CPQCI-F cryoprobe. The sample temperature was set to 20°C. The standard data was acquired using the excitation sculpting pulse program.[5] The STD data was acquired using a train of 180 Gaussian pulses for a total duration of 2.7s. On/off resonant frequencies were set to 0.5 PPM and 100 PPM.

The fragments were screened as pools of eight compounds per NMR tube and were added to pH 7.4 TRIS buffer, deuterium oxide containing NADH (50 µL per tube for NMR spectrometer stability) and the protein solution GRITS52989 (purity > 85%; protein concentration: 2.9 mg/mL) supplied by GSK[6] to yield solutions with a final volume of 500 µL. This gave final concentrations of 500 µM for each fragment, 7.6 µM InhA, 260 µM NADH, ~52 mM TRIS, 60 mM NaCl and 1.2 mM DTT. TWEEN-20 was added (0.01%) to limit potential aggregation of the ligands. The tubes were submitted to the NMR spectrometer using the automation software ICON-NMR. Due to the increased oxidation of NADH to NAD^+ in the presence of the InhA protein (see below) samples had to be prepared in small batches which limited throughput. Only 13 tubes (104 compounds) could be screened per day, this included the initial screen and a repeat in the presence of a known inhibitor of InhA (**1a**). To minimise acquisition time only the STD experiment[7] was performed to detect binding to InhA.

Loss of NADH STD signals in the presence of binders to InhA

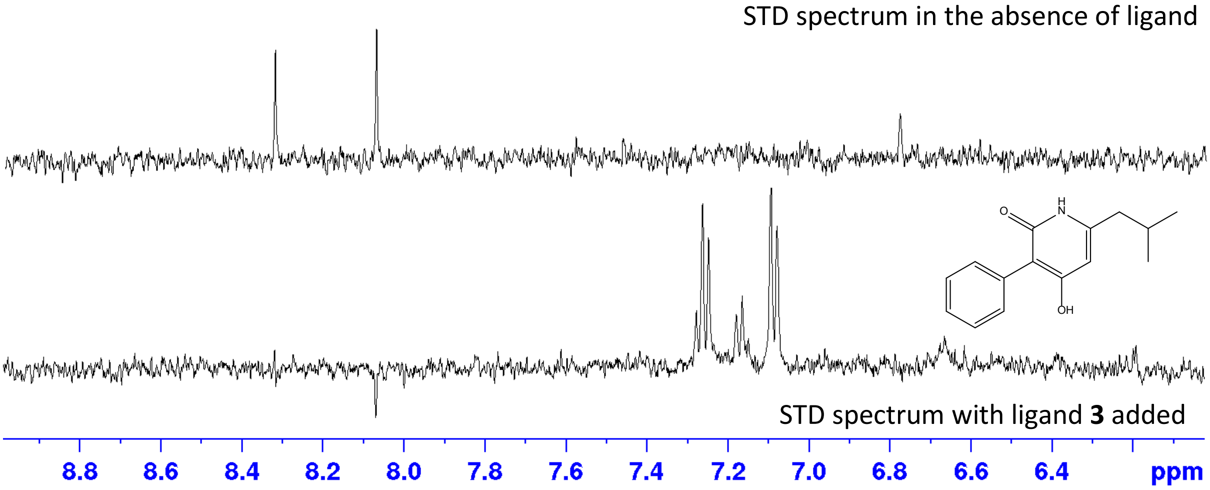

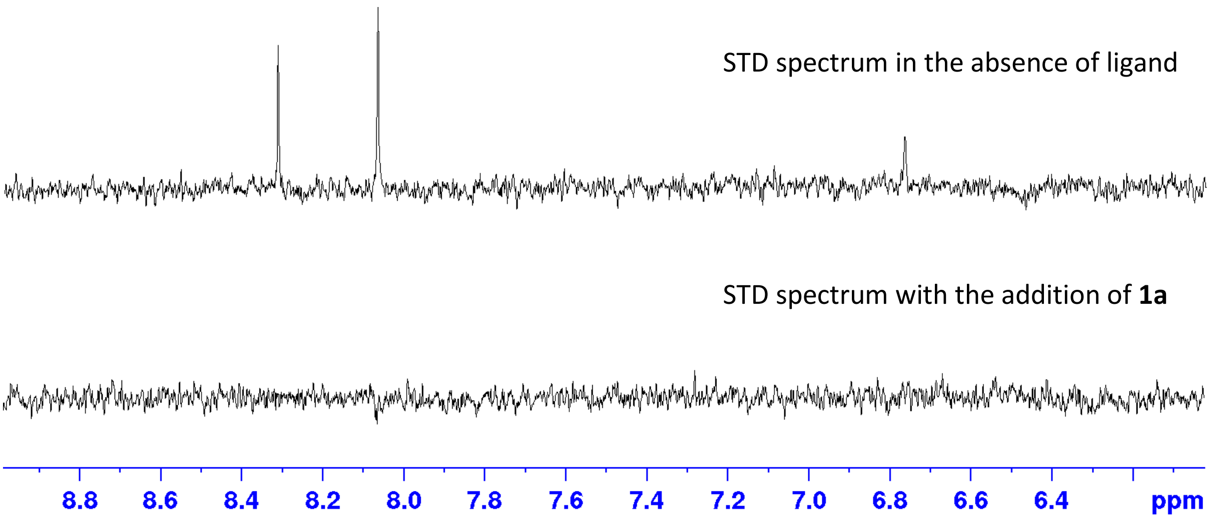

In both cases the addition of known inhibitors of InhA led to a loss of STD signals for HADH. This is not due to competition as both ligands and NADH bind in the active site simultaneously. A hypothesis is that the binding of the ligands increases the binding of NADH cooperatively. Therefore any pools of fragments which also reduce the NADH STD signals were of interest.

Stability of NADH in screening Buffer after ~ 20h at 25°C (no protein added)

The sample of NADH is stable in buffer under these conditions.

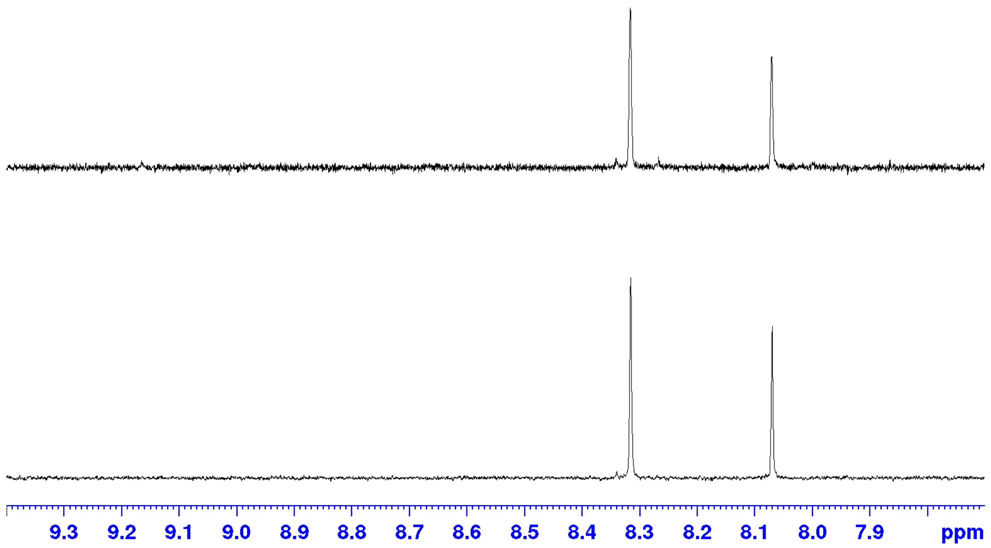

Stability of NADH in screening Buffer after ~ 6h at 25°C (10 μM InhA)

These spectra indicate the appearance of signals consistent with NAD^+ indicating that the InhA protein is catalysing the oxidation of NADH.

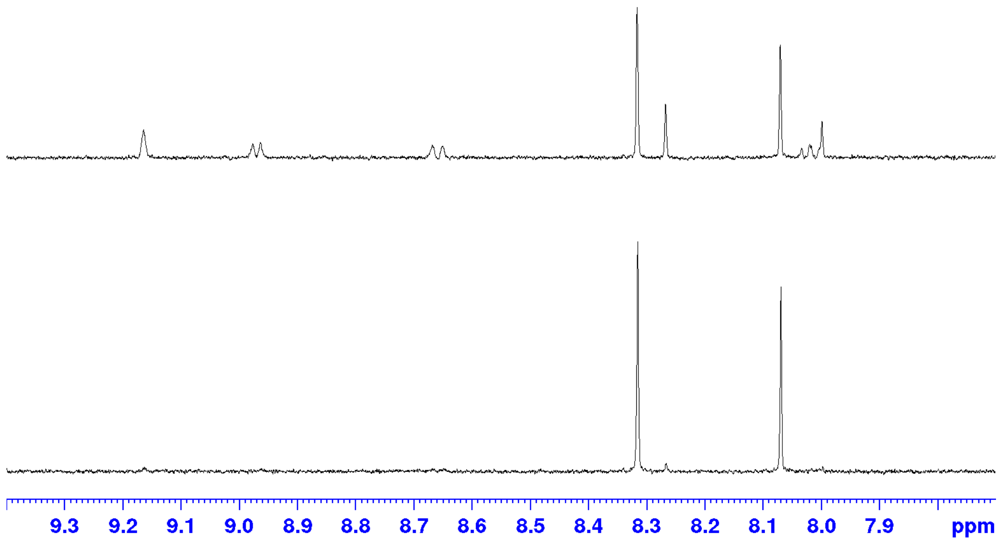

The spectra below show the pools containing positive hits from the NMR fragment screen and the reference spectra indicating the hits identified. The STD peaks for NADH are given for these pools also, with the STD peaks in the absence of fragments for comparison. The reduction STD intensity in some cases is somewhat subjective given some variation in NADH concentration between samples and some oxidation.

4

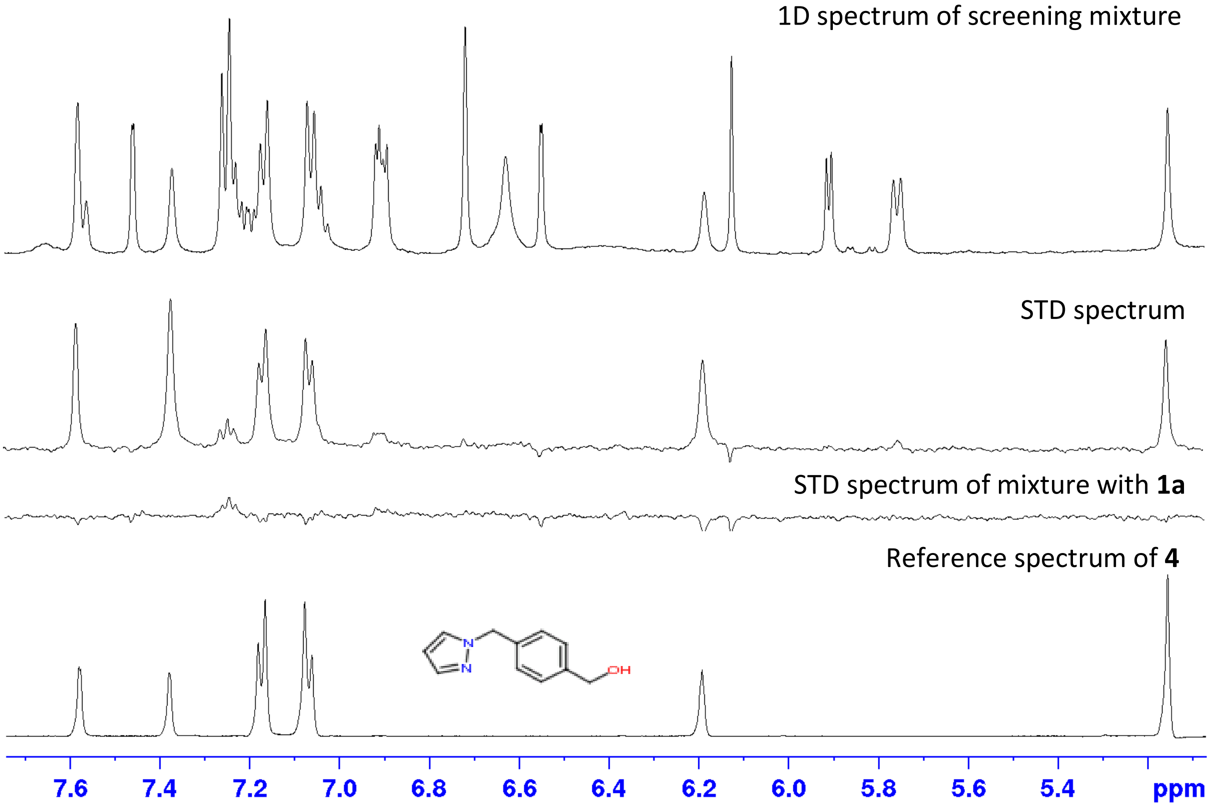
1D spectrum of screening mixture
STD spectrum
STD spectrum of mixture with 1a
Reference spectrum of 4
N
N
OH
7.6
7.4
7.2
7.0
6.8
6.6
6.4
6.2
6.0
5.8
5.6
5.4
ppm

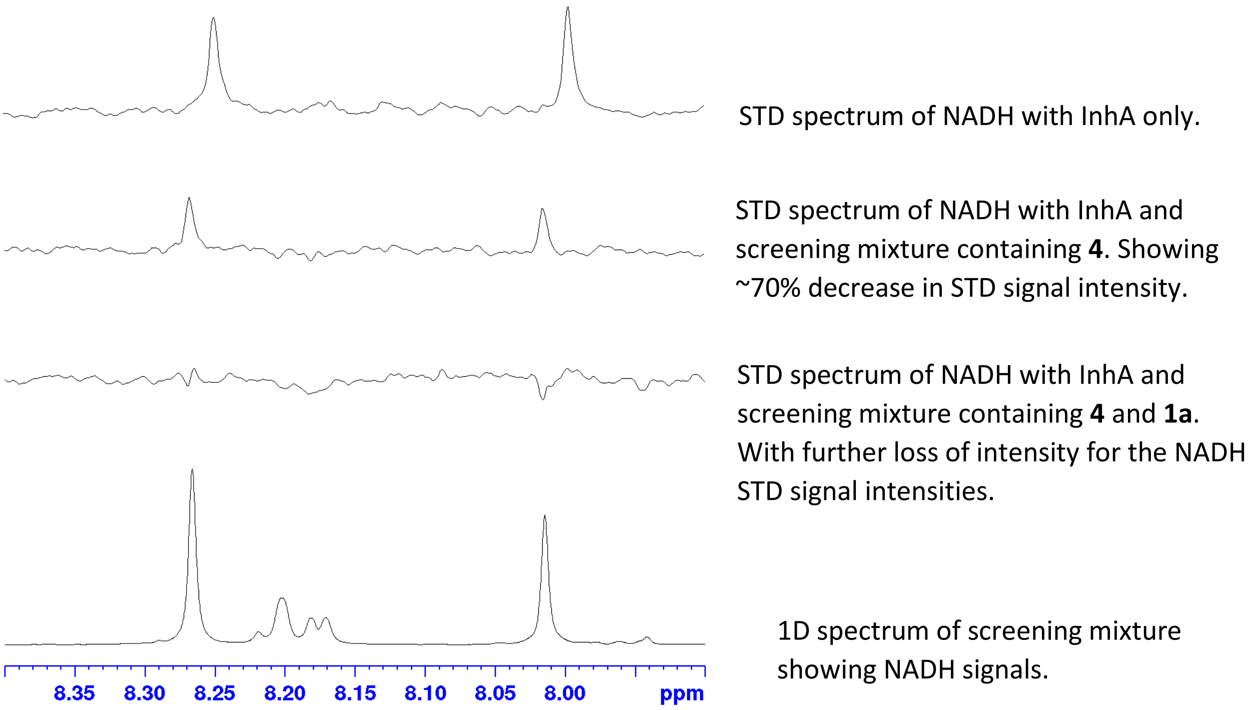
STD spectrum of NADH with InhA only.
STD spectrum of NADH with InhA and screening mixture containing 4. Showing ~70% decrease in STD signal intensity.
STD spectrum of NADH with InhA and screening mixture containing 4 and 1a. With further loss of intensity for the NADH STD signal intensities.
1D spectrum of screening mixture showing NADH signals.
8.35
8.30
8.25
8.20
8.15
8.10
8.05
8.00
ppm

5, 7, 10

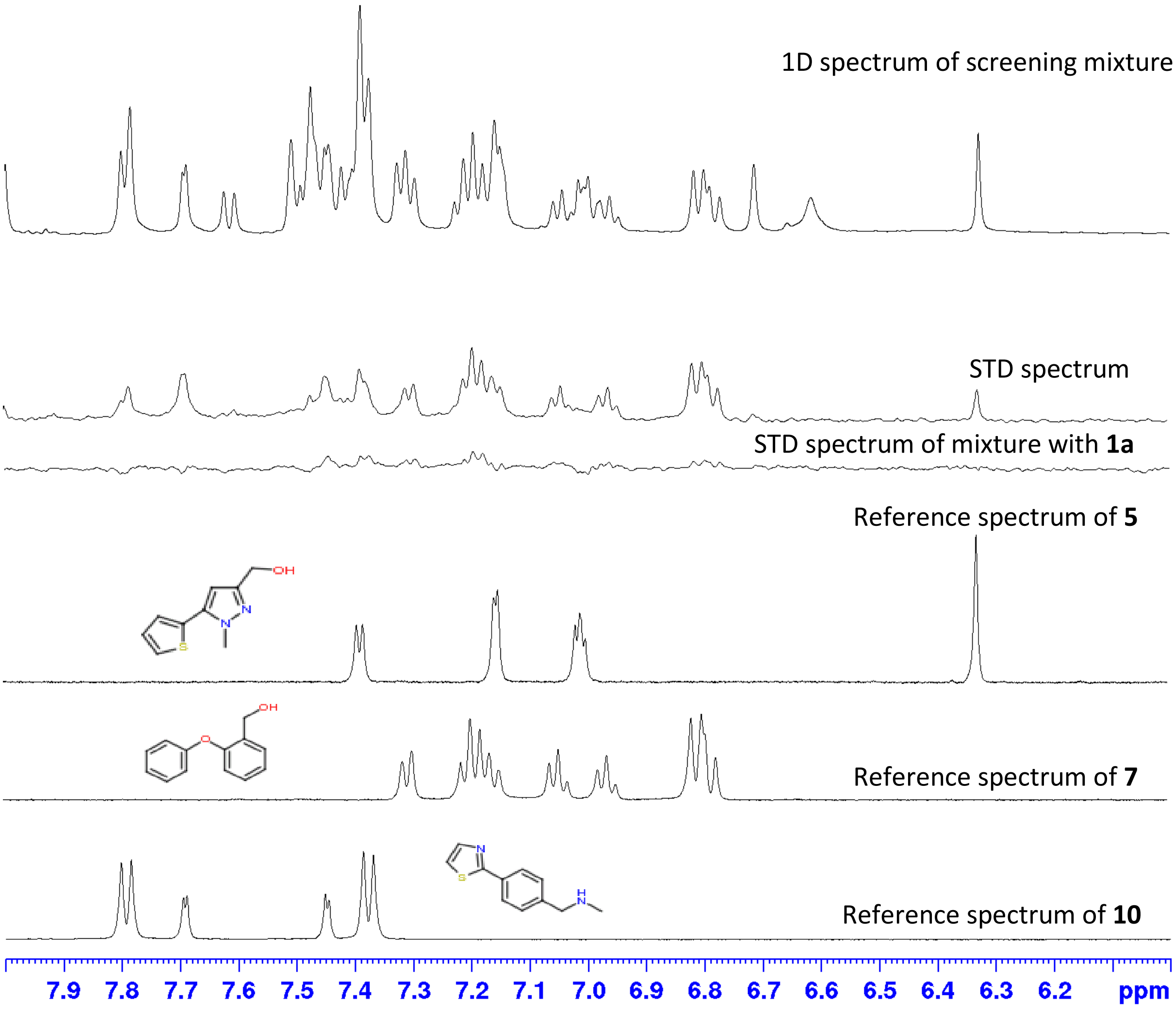
1D spectrum of screening mixture
STD spectrum
STD spectrum of mixture with **1a**
Reference spectrum of **5**
Reference spectrum of **7**
Reference spectrum of **10**
7.9 7.8 7.7 7.6 7.5 7.4 7.3 7.2 7.1 7.0 6.9 6.8 6.7 6.6 6.5 6.4 6.3 6.2 ppm

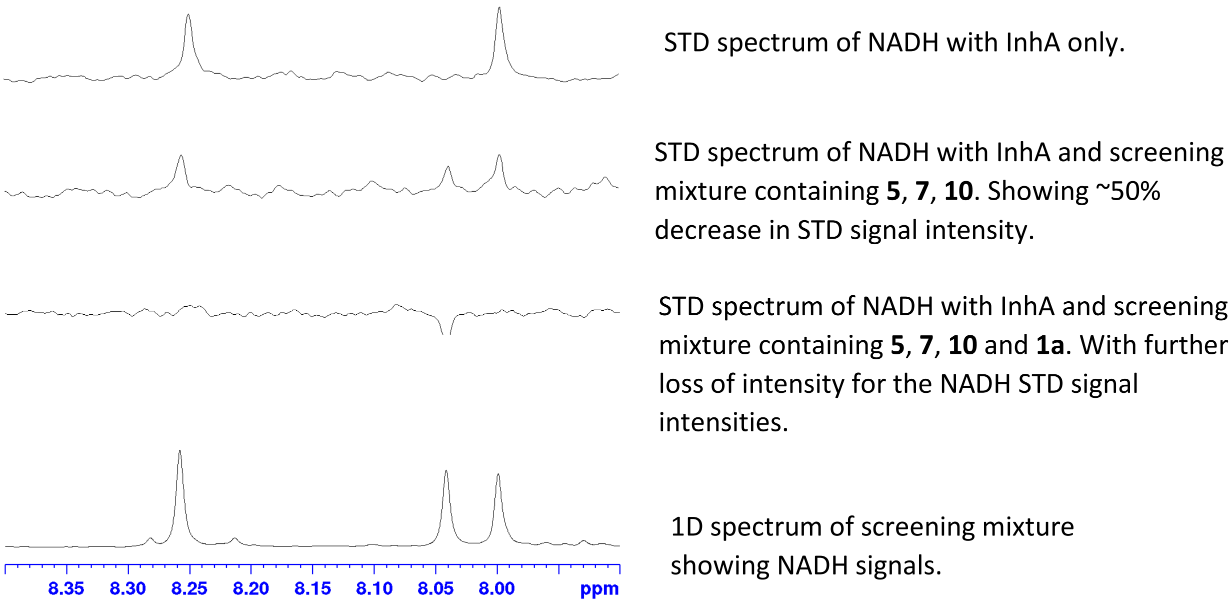
STD spectrum of NADH with InhA only.
STD spectrum of NADH with InhA and screening mixture containing **5**, **7**, **10**. Showing ~50% decrease in STD signal intensity.
STD spectrum of NADH with InhA and screening mixture containing **5**, **7**, **10** and **1a**. With further loss of intensity for the NADH STD signal intensities.
1D spectrum of screening mixture showing NADH signals.
8.35 8.30 8.25 8.20 8.15 8.10 8.05 8.00 ppm

6, 8

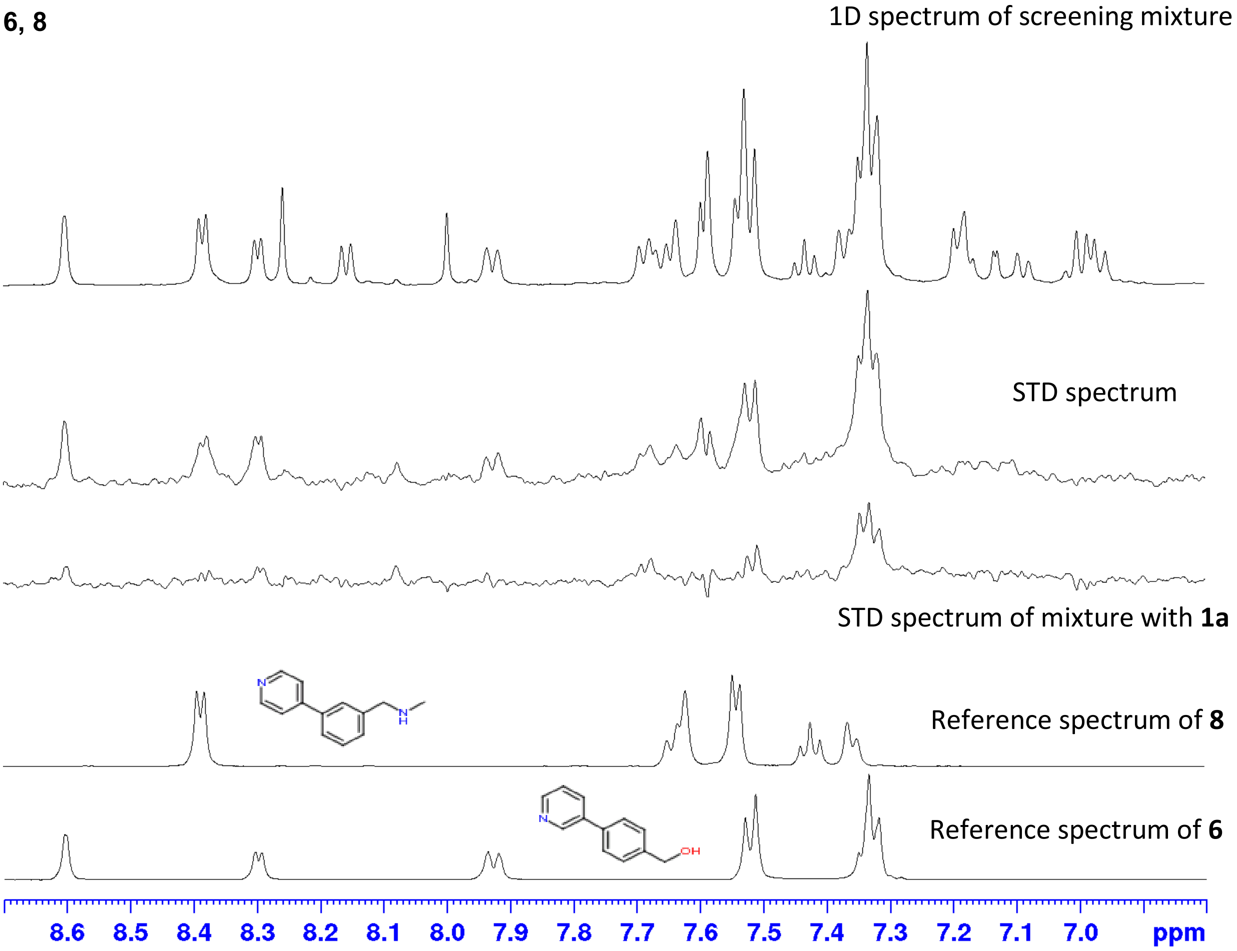
1D spectrum of screening mixture
STD spectrum
STD spectrum of mixture with **1a**
Reference spectrum of **8**
Reference spectrum of **6**
N
N
H
N
OH
8.6 8.5 8.4 8.3 8.2 8.1 8.0 7.9 7.8 7.7 7.6 7.5 7.4 7.3 7.2 7.1 7.0 ppm

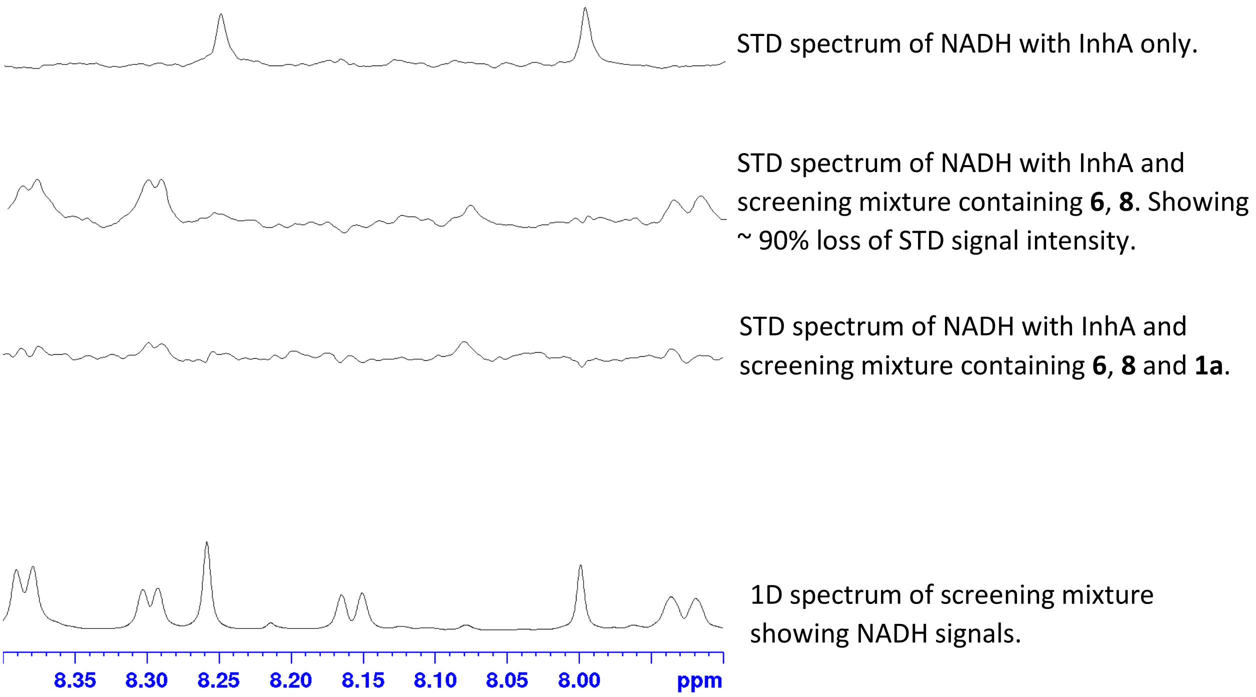
STD spectrum of NADH with InhA only.
STD spectrum of NADH with InhA and screening mixture containing **6**, **8**. Showing ~ 90% loss of STD signal intensity.
STD spectrum of NADH with InhA and screening mixture containing **6**, **8** and **1a**.
1D spectrum of screening mixture showing NADH signals.
8.35 8.30 8.25 8.20 8.15 8.10 8.05 8.00 ppm

9, 13

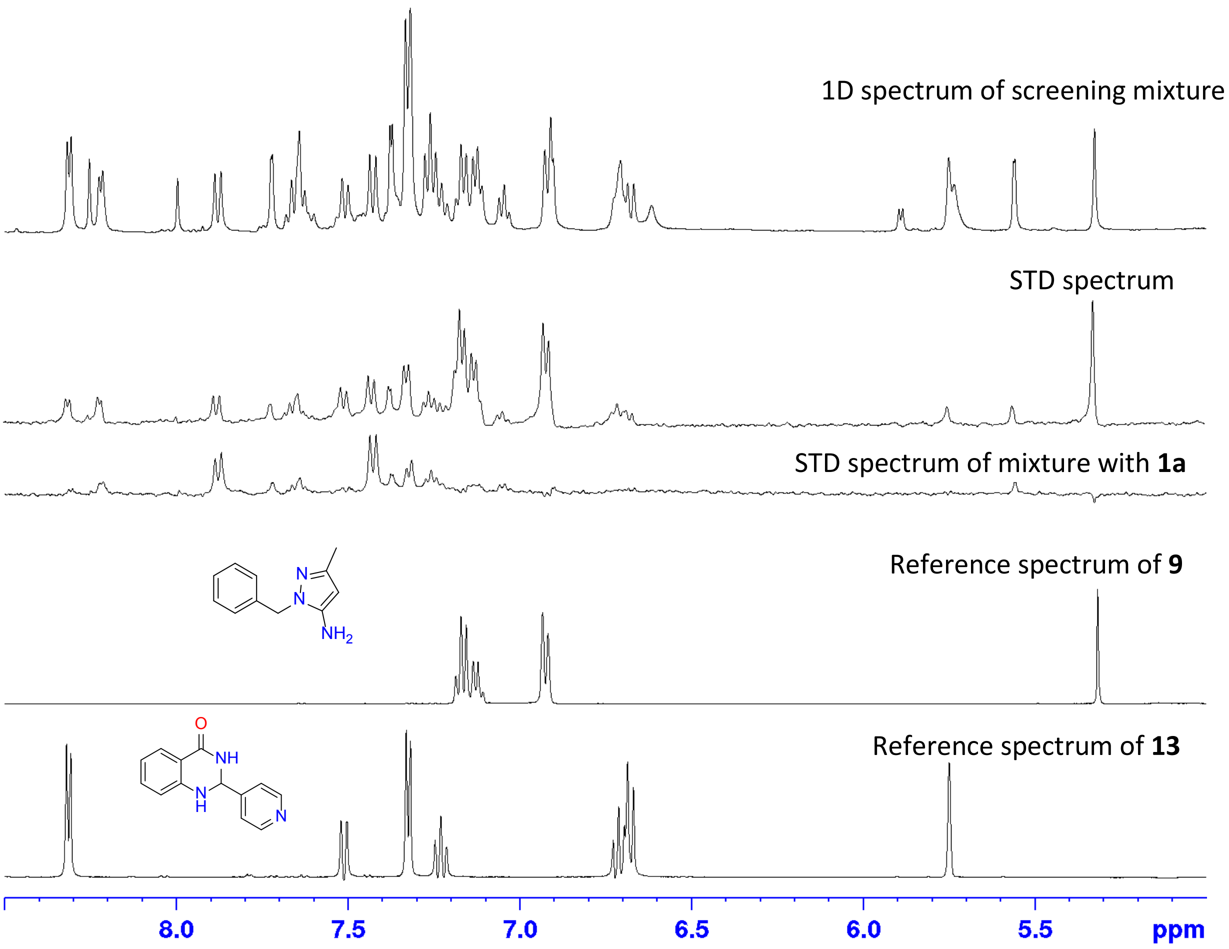

1D spectrum of screening mixture
STD spectrum
STD spectrum of mixture with **1a**
Reference spectrum of **9**
N
N
NH_2
Reference spectrum of **13**
O
NH
N
H
N
8.0
7.5
7.0
6.5
6.0
5.5
ppm

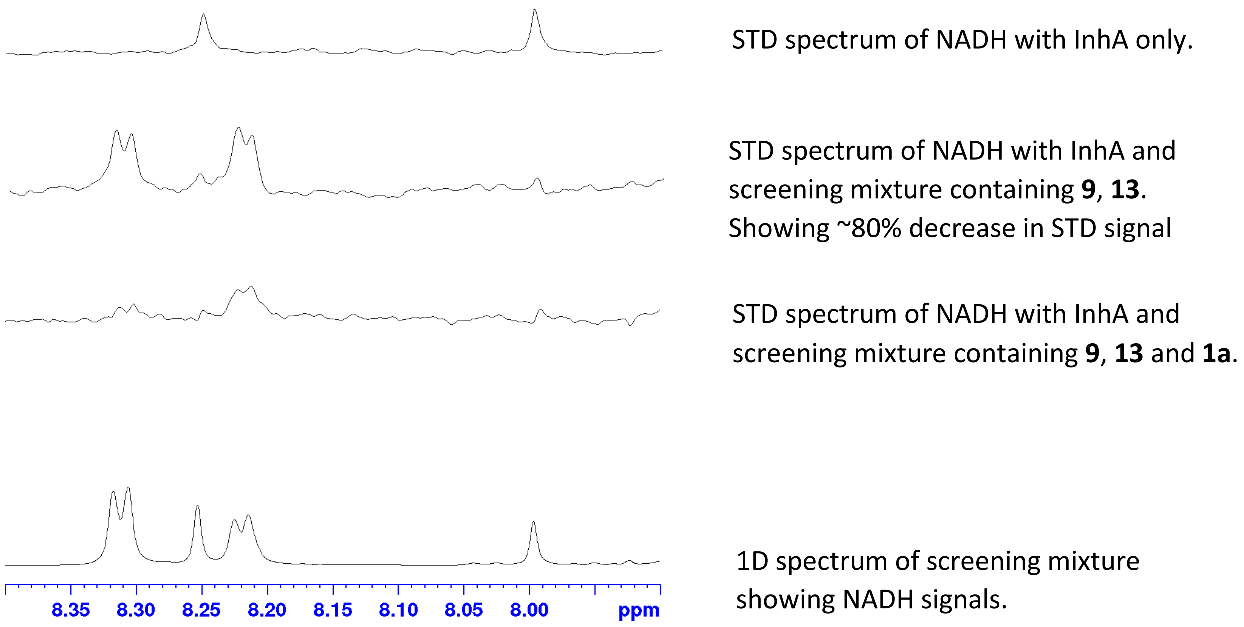

STD spectrum of NADH with InhA only.
STD spectrum of NADH with InhA and screening mixture containing **9**, **13**. Showing ~80% decrease in STD signal
STD spectrum of NADH with InhA and screening mixture containing **9**, **13** and **1a**.
1D spectrum of screening mixture showing NADH signals.
8.35
8.30
8.25
8.20
8.15
8.10
8.05
8.00
ppm

11

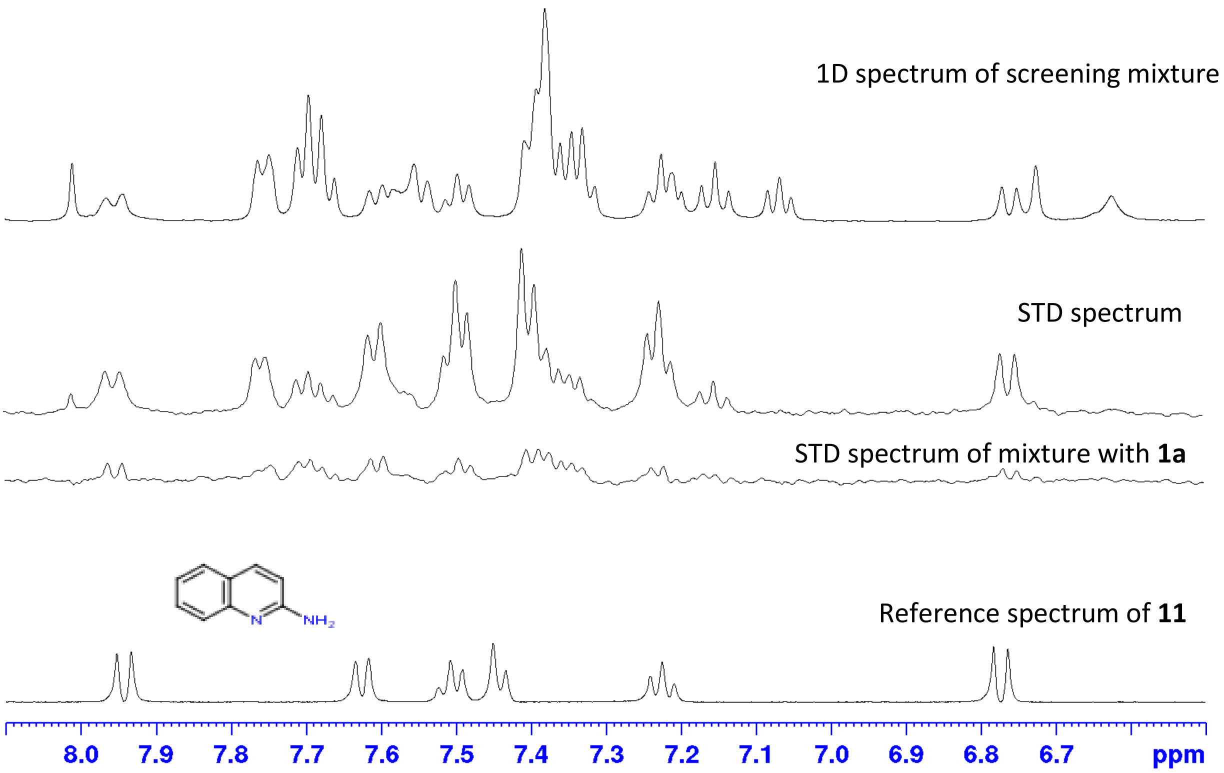

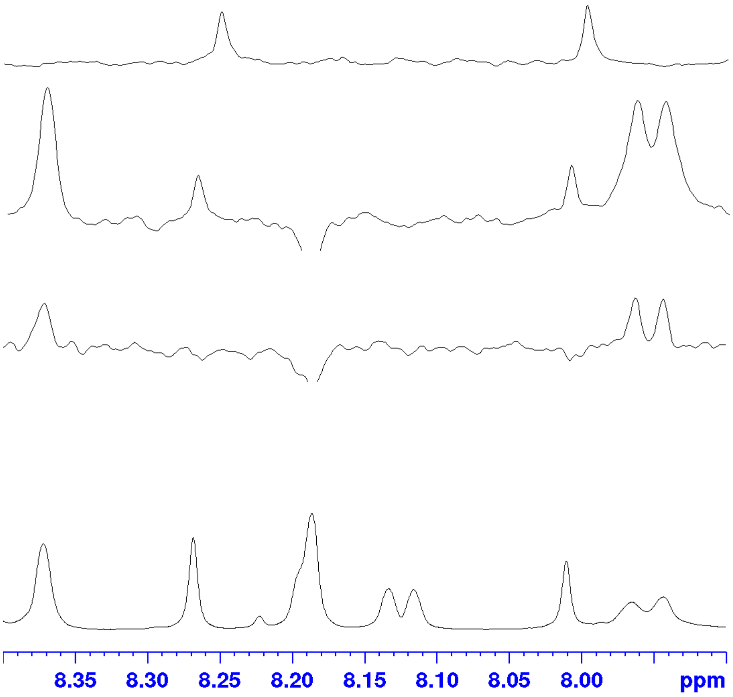

STD spectrum of NADH with InhA only.

STD spectrum of NADH with InhA and screening mixture containing **11**. Showing ~ 40% decrease in STD signal.

STD spectrum of NADH with InhA and screening mixture containing **11** and **1a**. With further loss of intensity for the NADH STD signal intensities.

1D spectrum of screening mixture showing NADH signals.

12

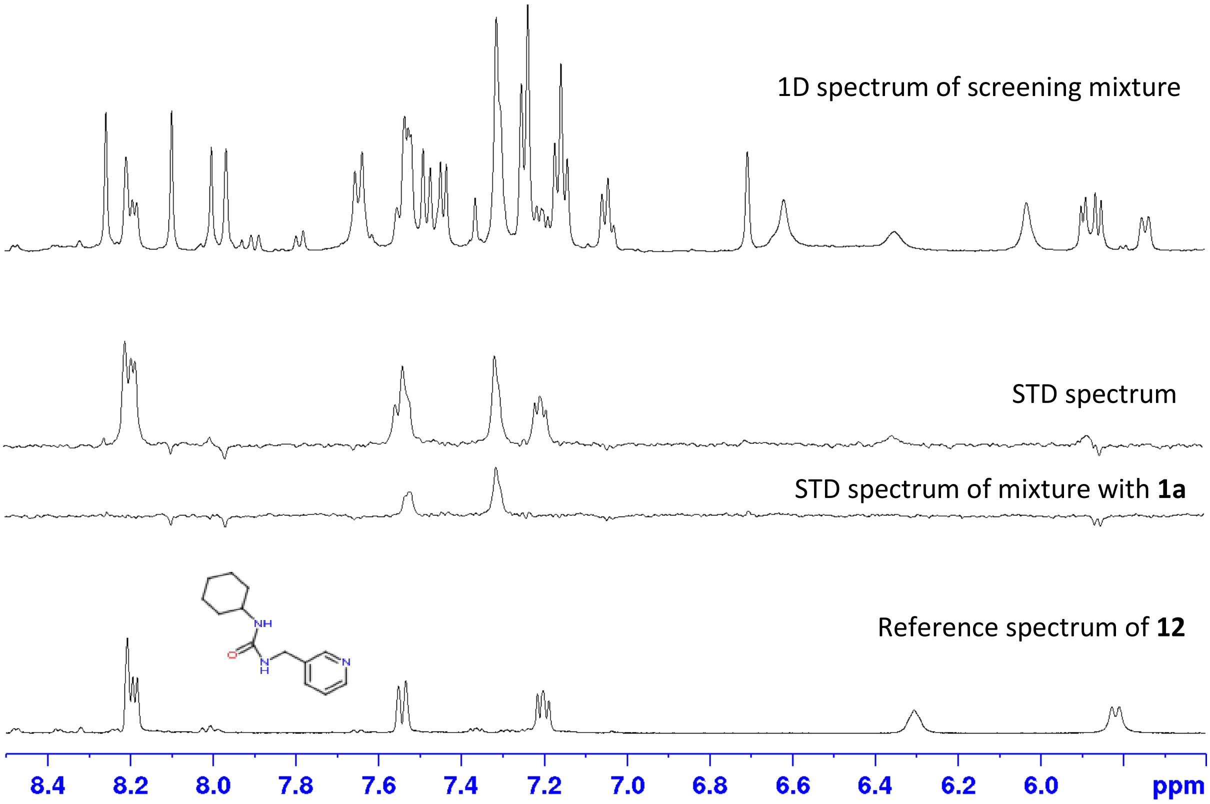

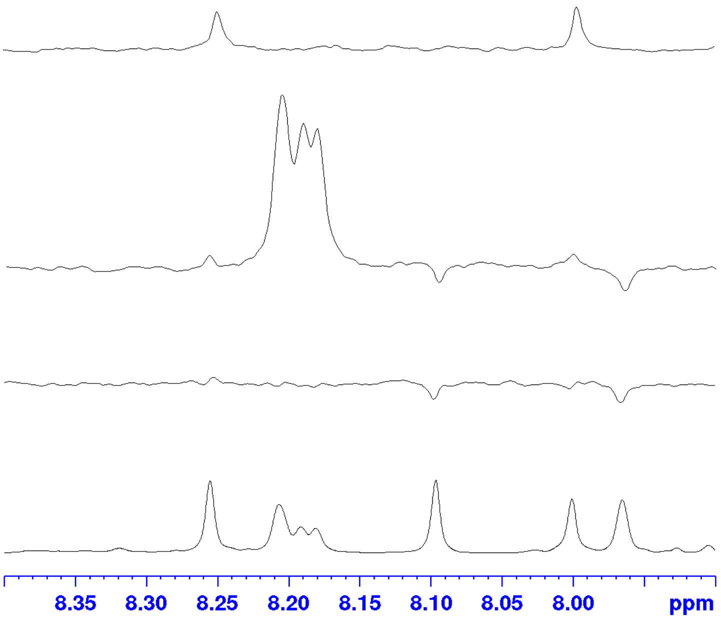

STD spectrum of NADH with InhA only.

STD spectrum of NADH with InhA and screening mixture containing **12**. Showing ~60% decrease in STD signal intensity.

STD spectrum of NADH with InhA and screening mixture containing **12** and **1a**. With further loss of intensity for the NADH STD signal intensities.

1D spectrum of screening mixture showing NADH signals.

14

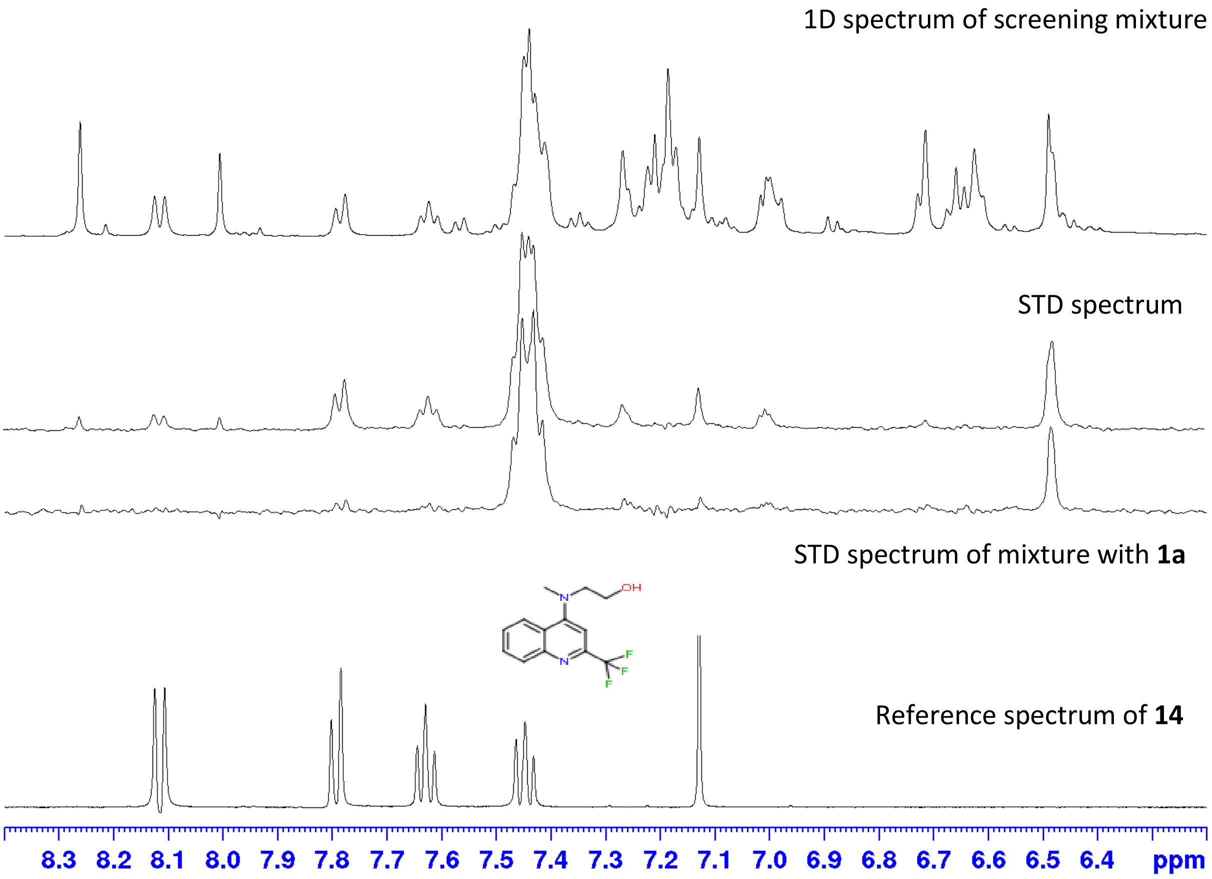
1D spectrum of screening mixture
STD spectrum
STD spectrum of mixture with **1a**
OH
N
N
F
F
F
Reference spectrum of **14**
8.3 8.2 8.1 8.0 7.9 7.8 7.7 7.6 7.5 7.4 7.3 7.2 7.1 7.0 6.9 6.8 6.7 6.6 6.5 6.4 ppm

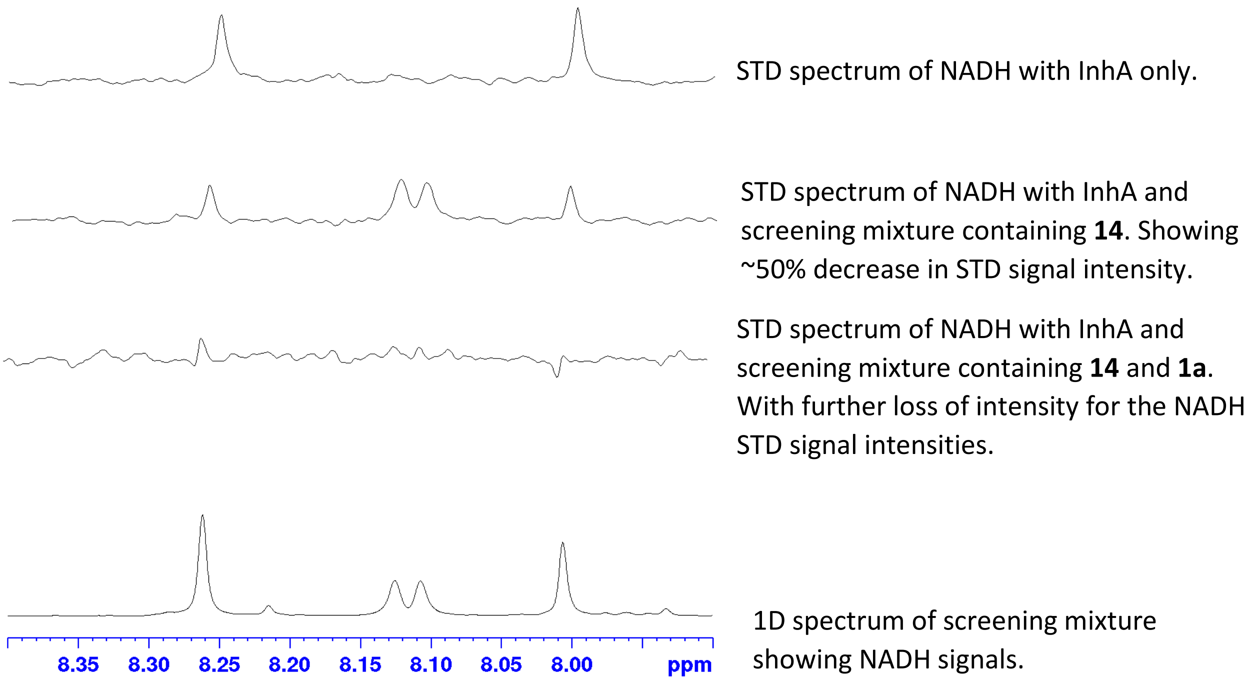
STD spectrum of NADH with InhA only.
STD spectrum of NADH with InhA and screening mixture containing **14**. Showing ~50% decrease in STD signal intensity.
STD spectrum of NADH with InhA and screening mixture containing **14** and **1a**. With further loss of intensity for the NADH STD signal intensities.
1D spectrum of screening mixture showing NADH signals.
8.35 8.30 8.25 8.20 8.15 8.10 8.05 8.00 ppm

15, 24, 27

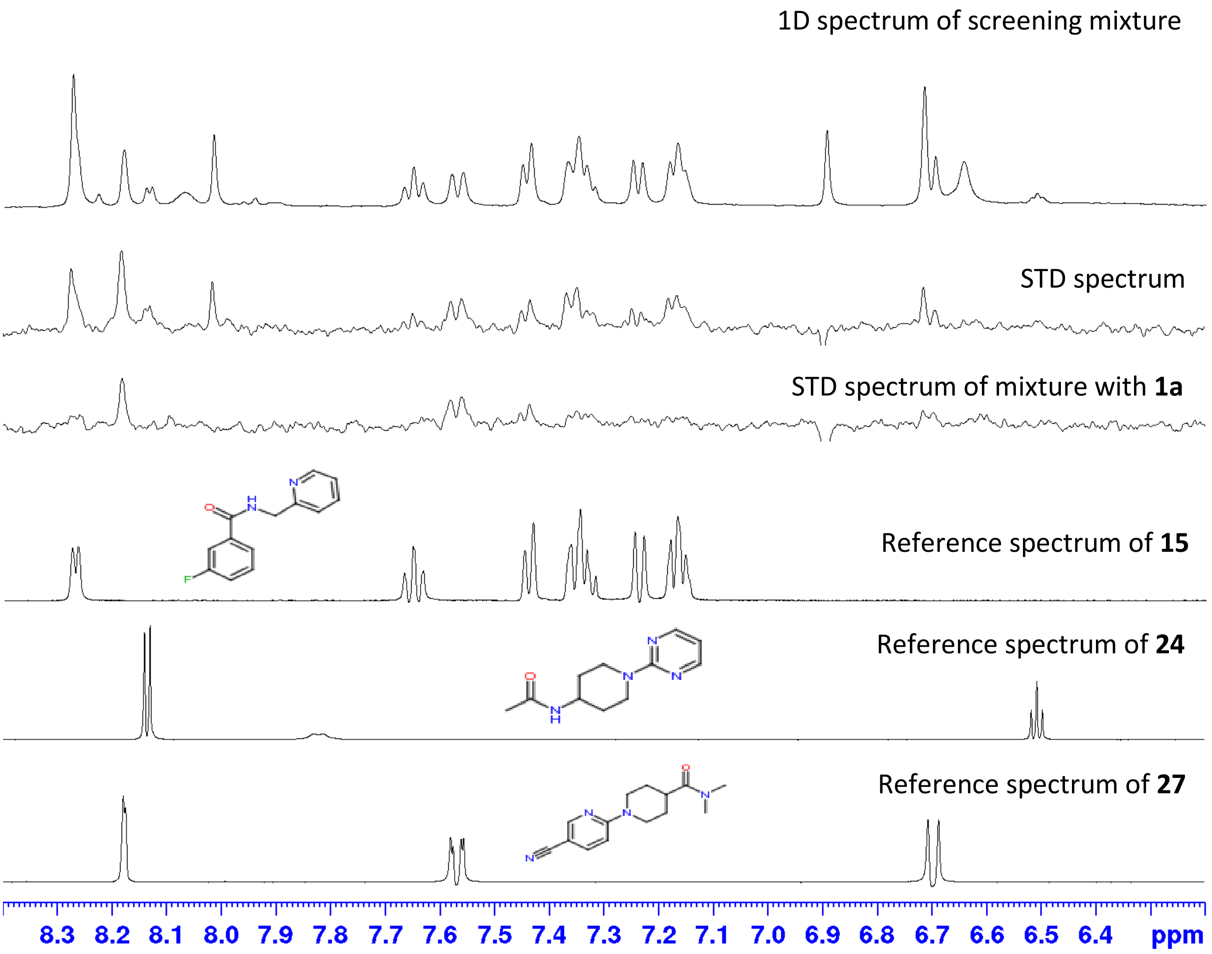

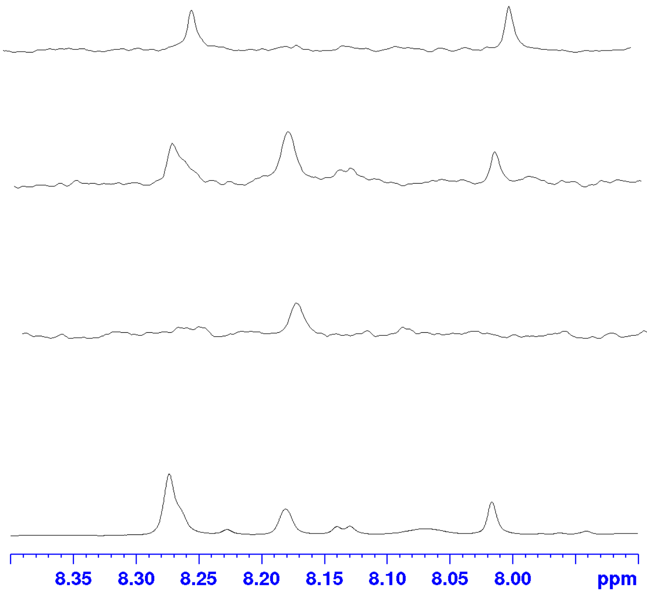

STD spectrum of NADH with InhA only.

STD spectrum of NADH with InhA and screening mixture containing **15**, **24**, **27**. Showing ~ 45% decrease in STD signal intensity.

STD spectrum of NADH with InhA and screening mixture containing **15**, **24**, **27** and **1a**. With further loss of intensity for the NADH STD signal intensities.

1D spectrum of screening mixture showing NADH signals.

16

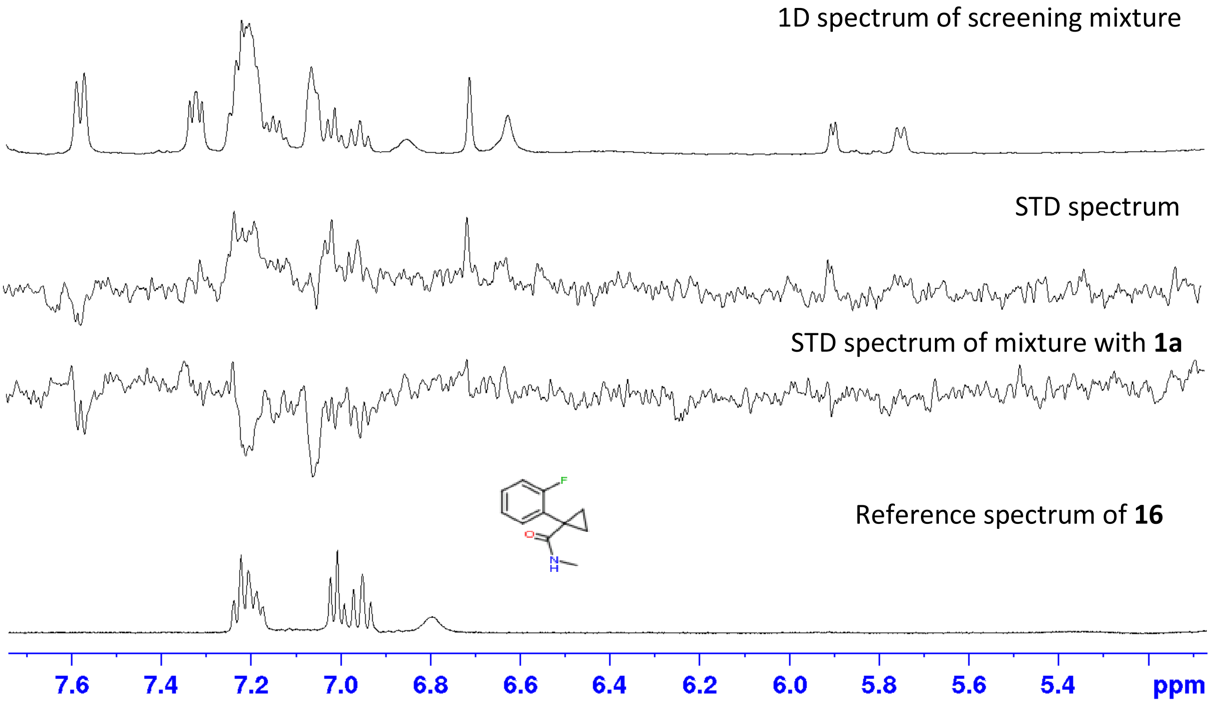

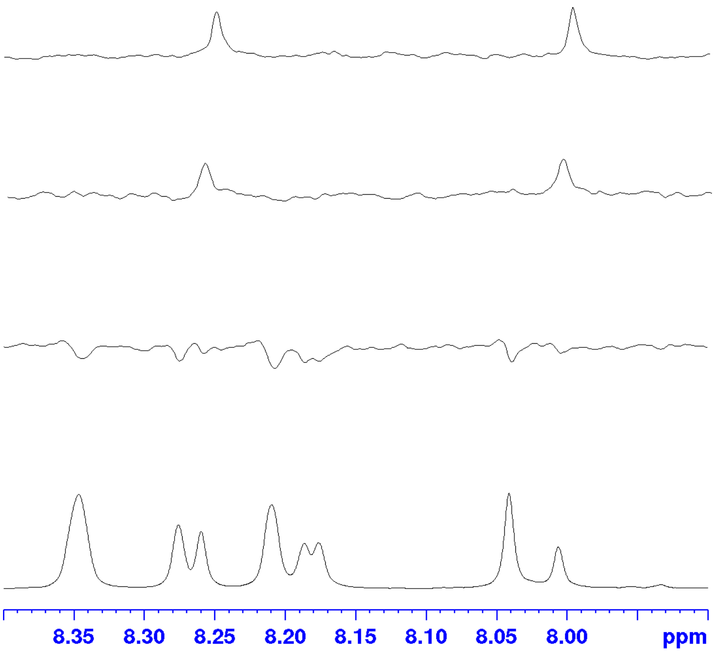

STD spectrum of NADH with InhA only.

STD spectrum of NADH with InhA and screening mixture containing **16**. Showing ~40% decrease in STD signal intensity for NADH.

STD spectrum of NADH with InhA and screening mixture containing **16** and **1a**. With further loss of intensity for the NADH STD signal intensities.

1D spectrum of screening mixture showing NADH signals.

17, 25

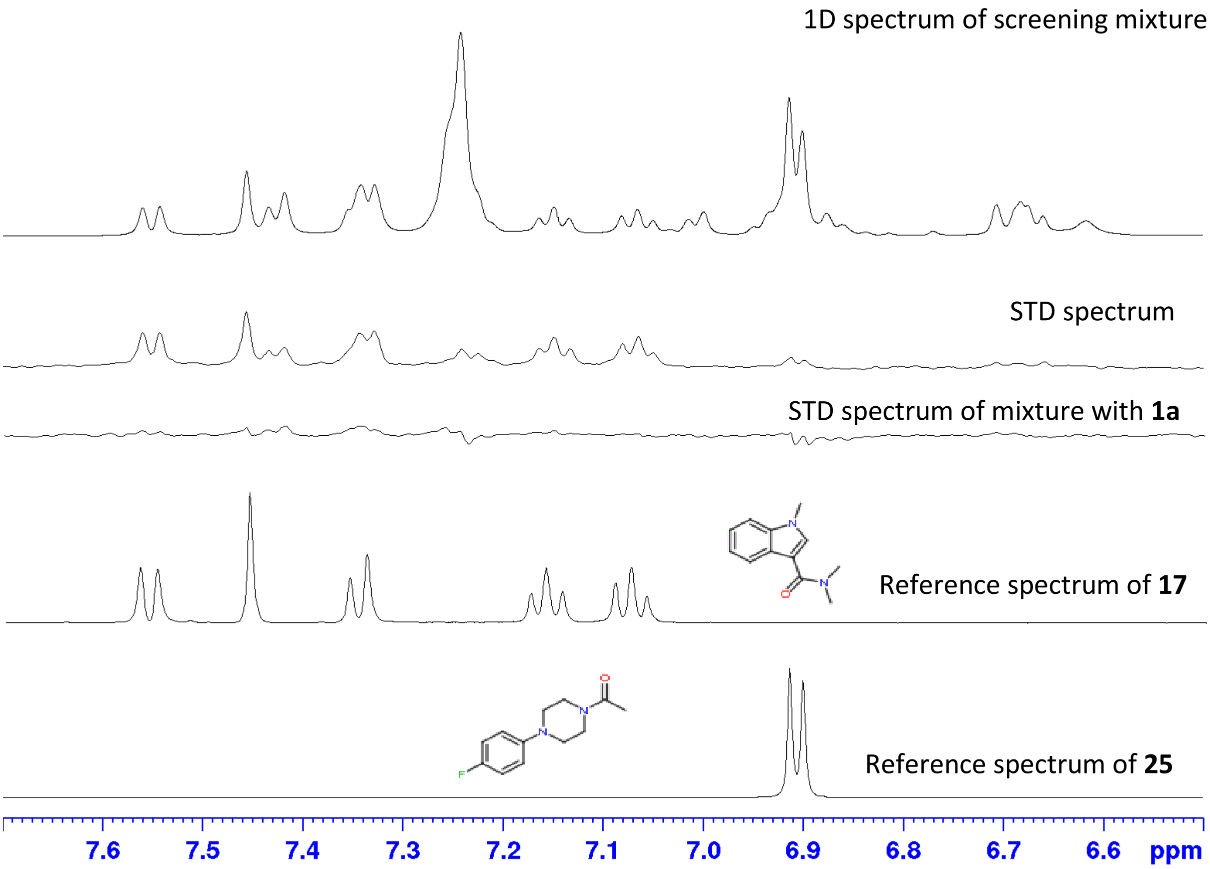
1D spectrum of screening mixture
STD spectrum
STD spectrum of mixture with 1a
Reference spectrum of 17
Reference spectrum of 25
7.6
7.5
7.4
7.3
7.2
7.1
7.0
6.9
6.8
6.7
6.6
ppm

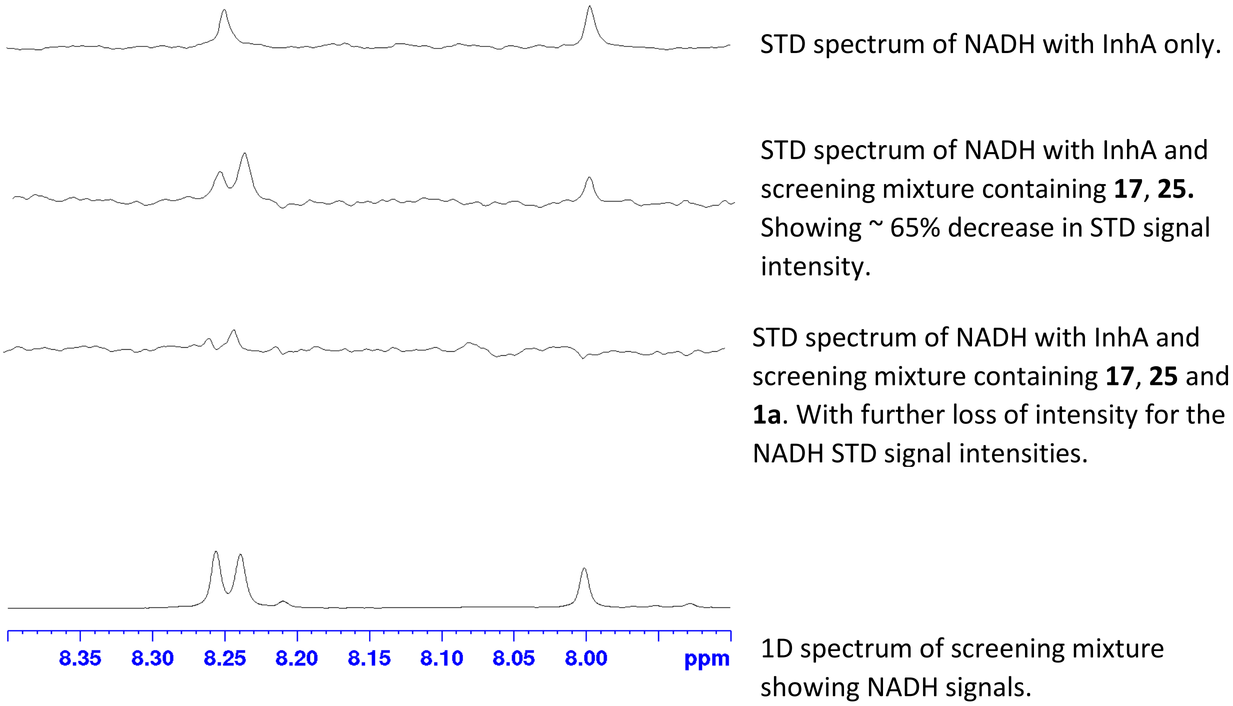
STD spectrum of NADH with InhA only.
STD spectrum of NADH with InhA and screening mixture containing 17, 25. Showing ~ 65% decrease in STD signal intensity.
STD spectrum of NADH with InhA and screening mixture containing 17, 25 and 1a. With further loss of intensity for the NADH STD signal intensities.
1D spectrum of screening mixture showing NADH signals.
8.35
8.30
8.25
8.20
8.15
8.10
8.05
8.00
ppm

18, 26

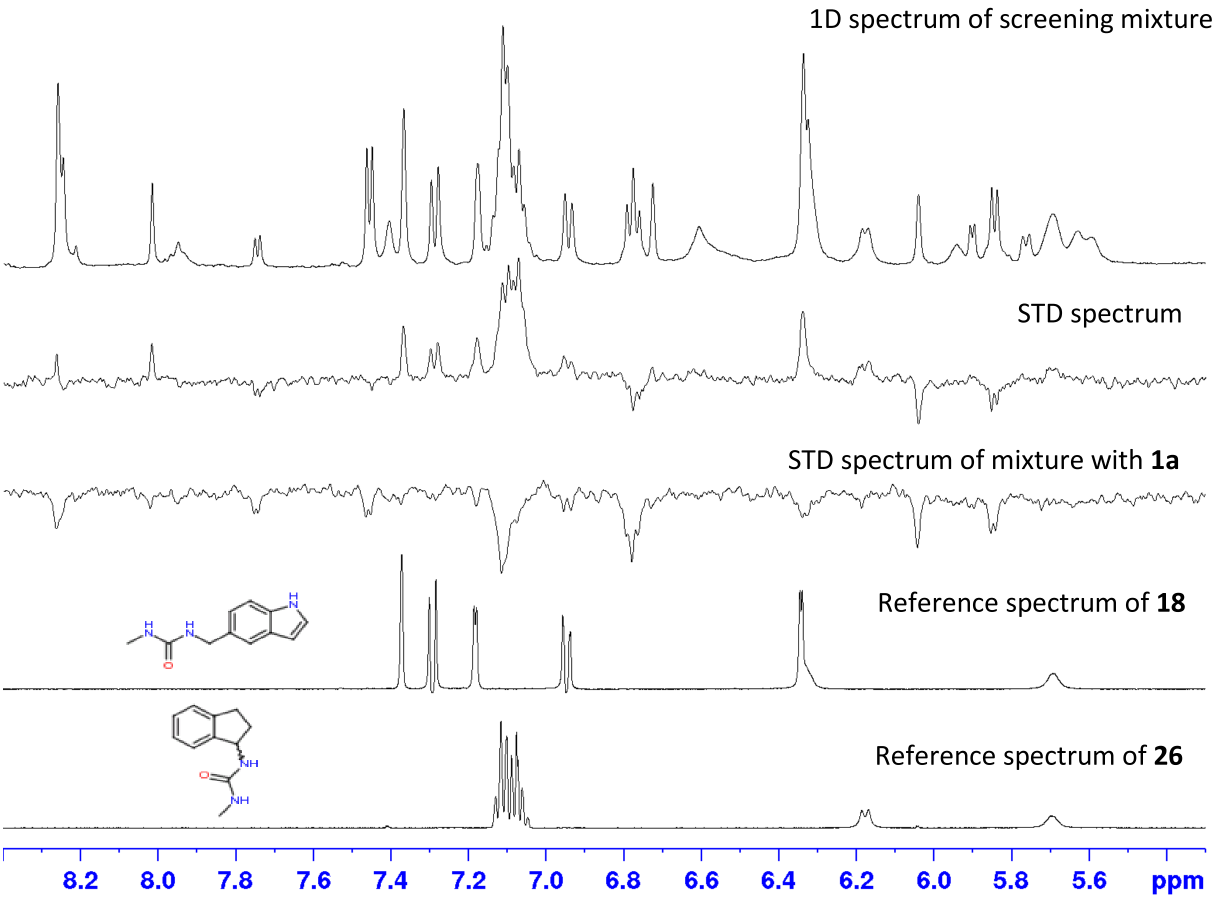

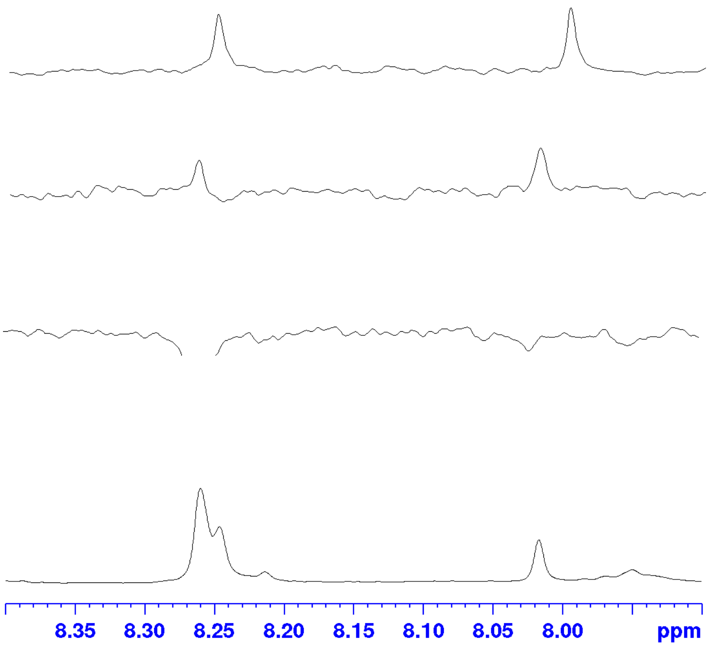

STD spectrum of NADH with InhA only.

STD spectrum of NADH with InhA and screening mixture containing **18**, **26**. Showing ~40% decrease in STD signal intensity.

STD spectrum of NADH with InhA and screening mixture containing **18**, **26** and **1a**. With further loss of intensity for the NADH STD signal intensities.

1D spectrum of screening mixture showing NADH signals.

19, 23

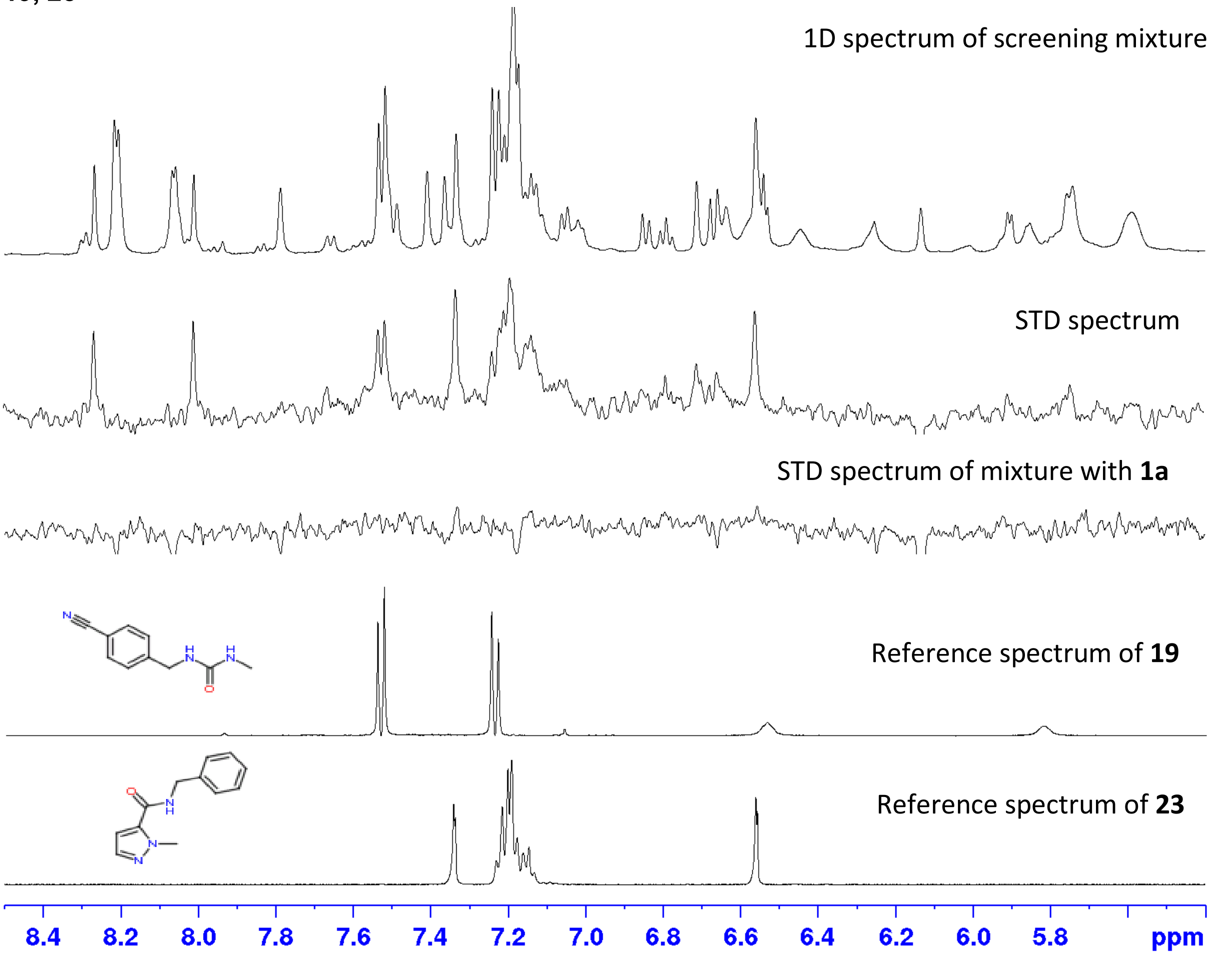

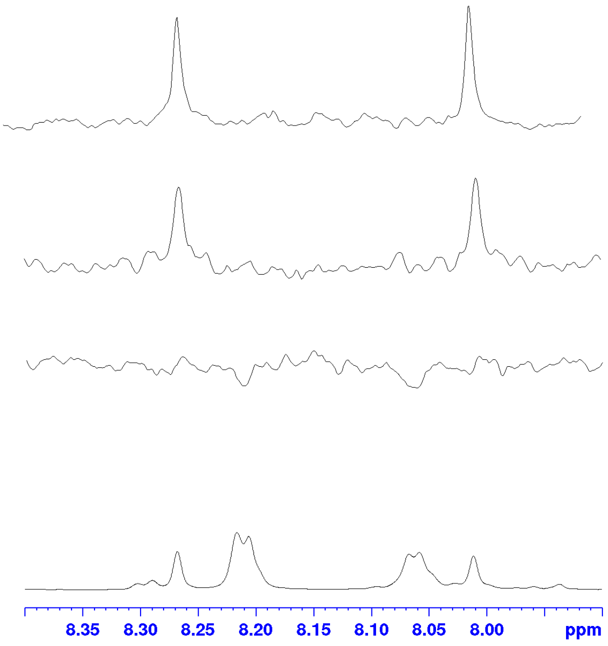

STD spectrum of NADH with InhA only.

STD spectrum of NADH with InhA and screening mixture containing **19**, **23**. Showing ~25% decrease in STD signal intensity.

STD spectrum of NADH with InhA and screening mixture containing **19**, **23** and **1a**. With further loss of intensity for the NADH STD signal intensities.

1D spectrum of screening mixture showing NADH signals.

20

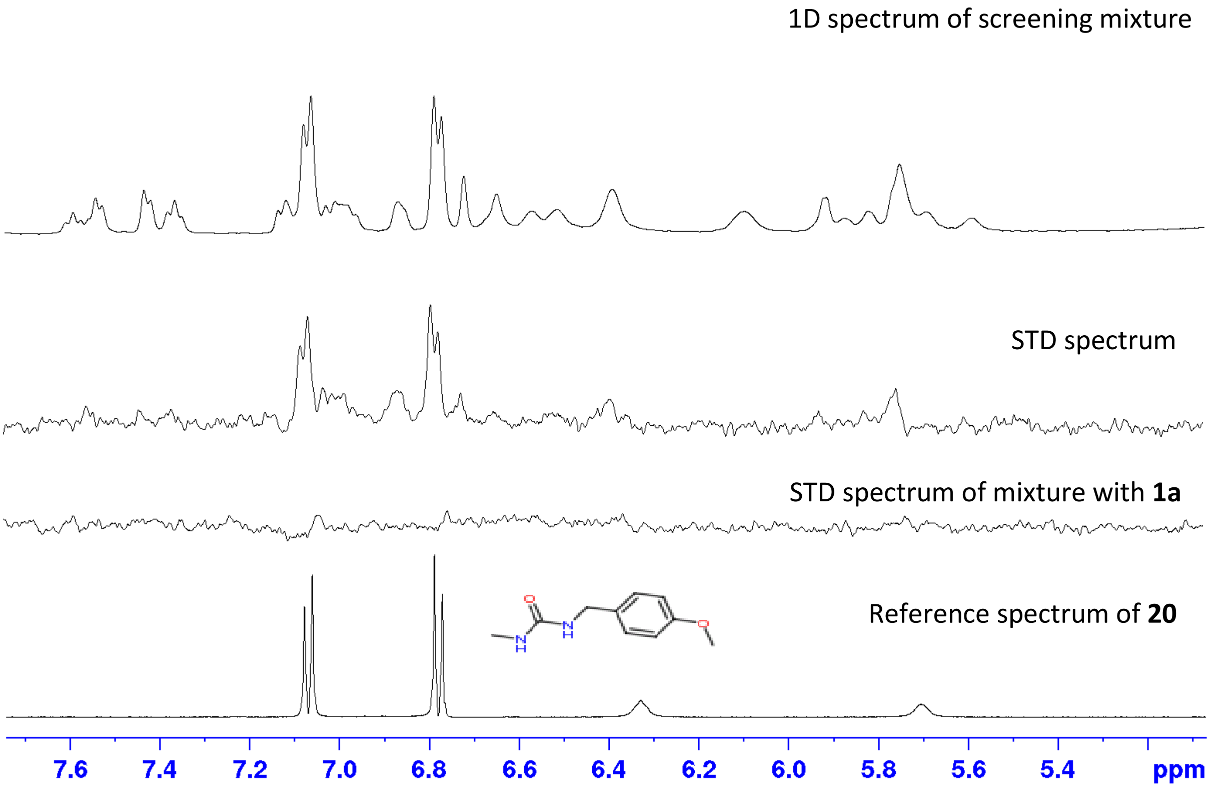
1D spectrum of screening mixture
STD spectrum
STD spectrum of mixture with **1a**
Reference spectrum of **20**
7.6 7.4 7.2 7.0 6.8 6.6 6.4 6.2 6.0 5.8 5.6 5.4 ppm

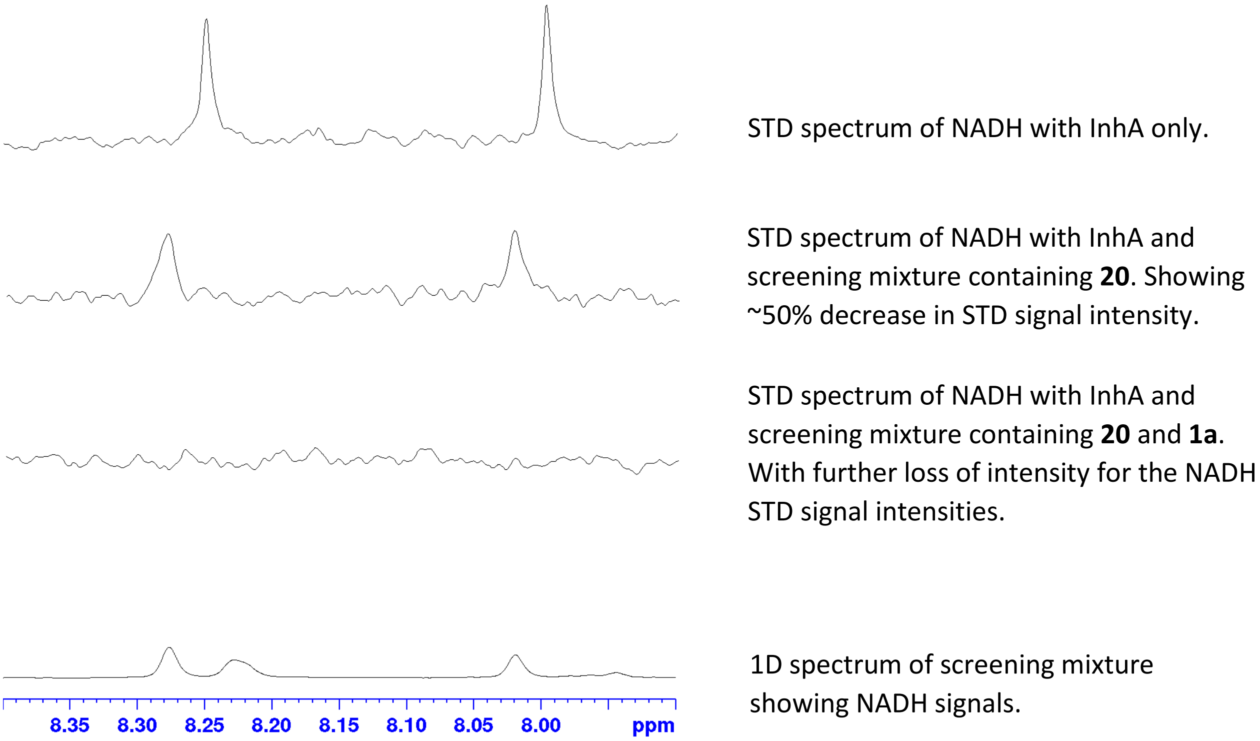
STD spectrum of NADH with InhA only.
STD spectrum of NADH with InhA and screening mixture containing **20**. Showing ~50% decrease in STD signal intensity.
STD spectrum of NADH with InhA and screening mixture containing **20** and **1a**. With further loss of intensity for the NADH STD signal intensities.
1D spectrum of screening mixture showing NADH signals.
8.35 8.30 8.25 8.20 8.15 8.10 8.05 8.00 ppm

21, 22

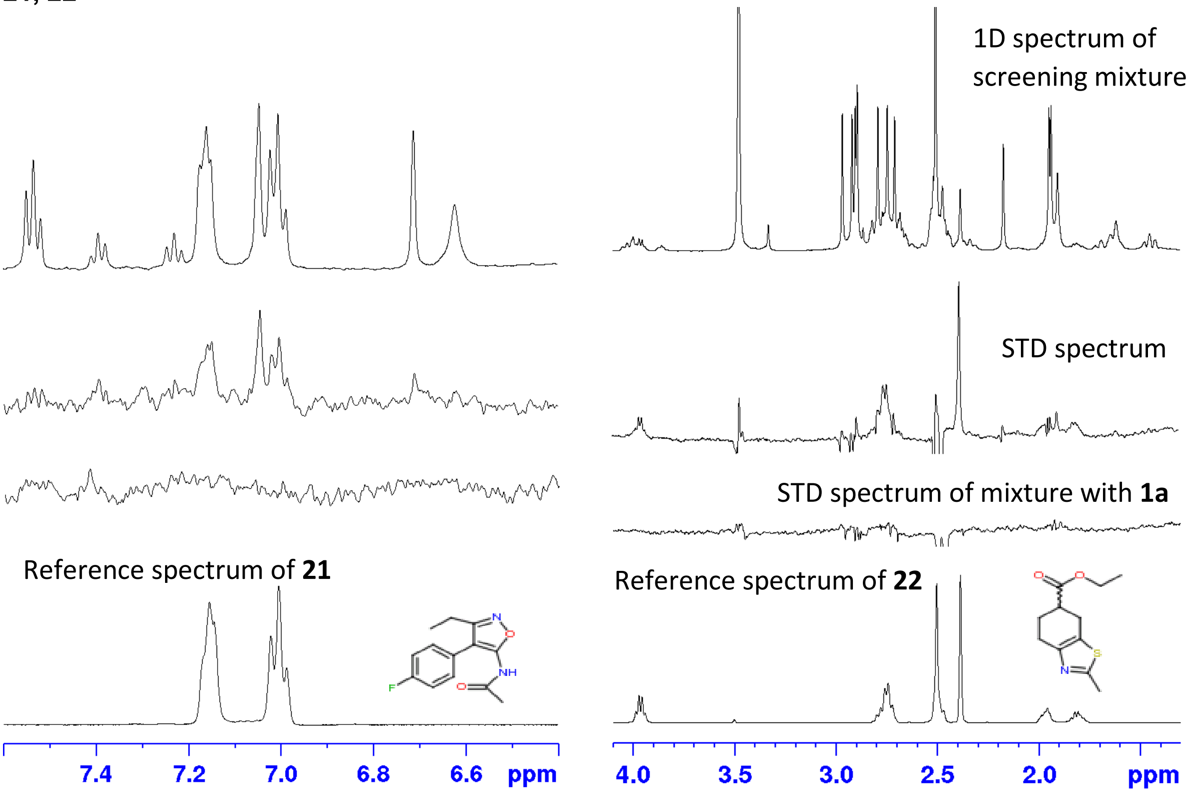

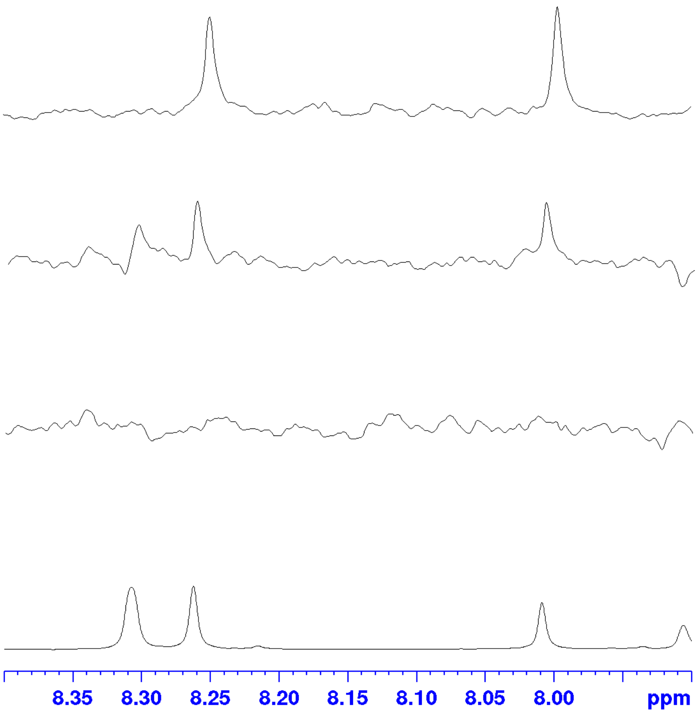

STD spectrum of NADH with InhA only.

STD spectrum of NADH with InhA and screening mixture containing **21**, **22**. Showing ~70% decrease in STD signal intensity.

STD spectrum of NADH with InhA and screening mixture containing **21**, **22** and **1a**. With further loss of intensity for the NADH STD signal intensities.

1D spectrum of screening mixture showing NADH signals.

28, 30, 31

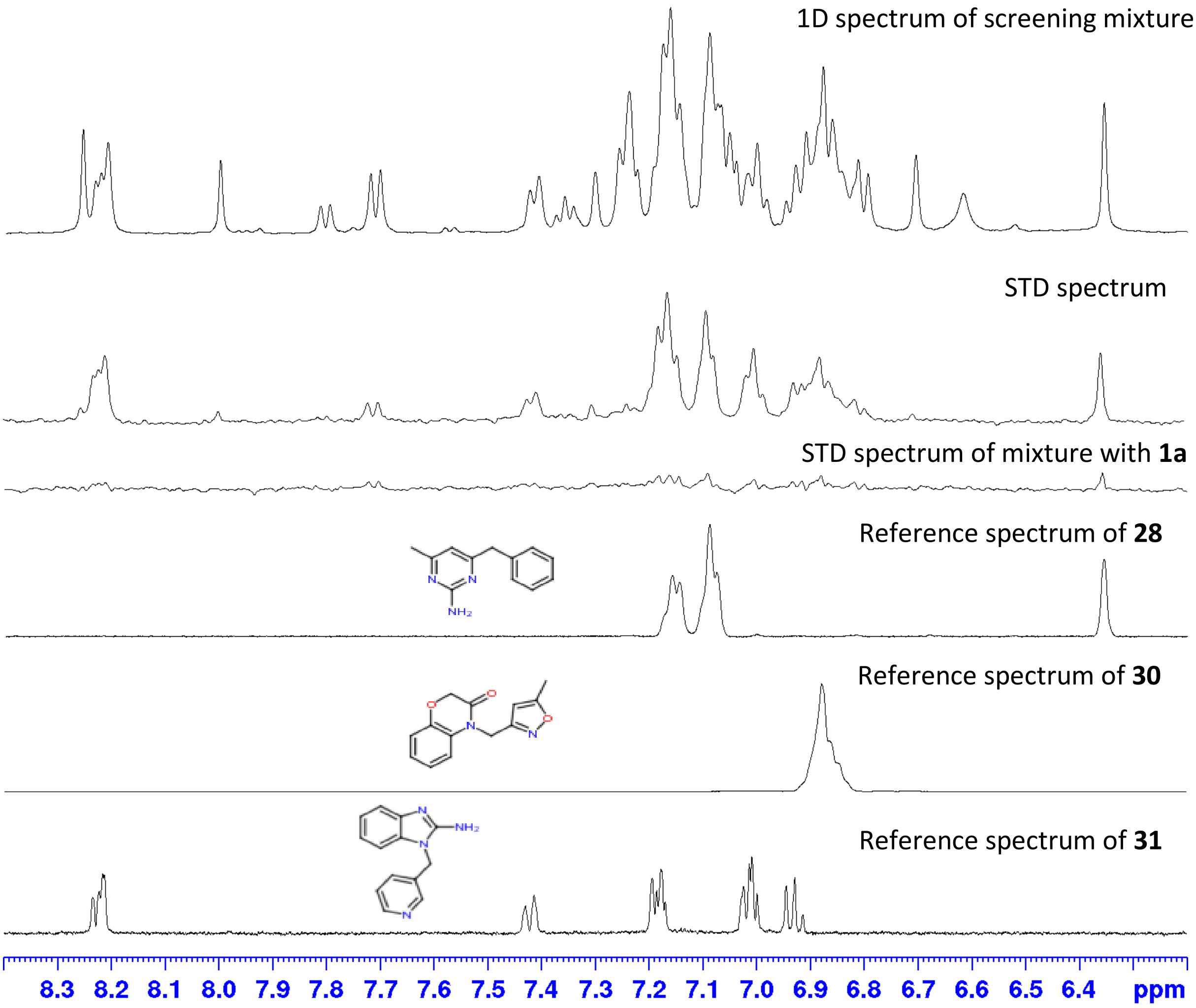

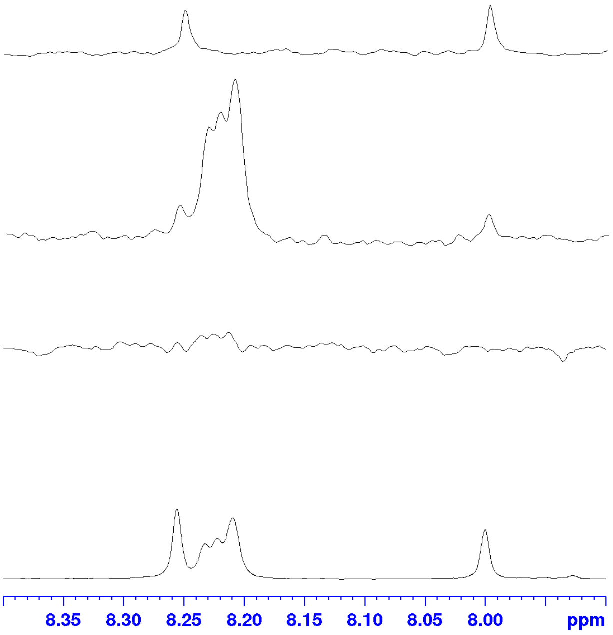

STD spectrum of NADH with InhA only.

STD spectrum of NADH with InhA and screening mixture containing **28**, **30**, **31**. Showing ~50% decrease in STD signal intensity.

STD spectrum of NADH with InhA and screening mixture containing **28**, **30**, **31** and **1a**. With further loss of intensity for the NADH STD signal intensities.

1D spectrum of screening mixture showing NADH signals.

29

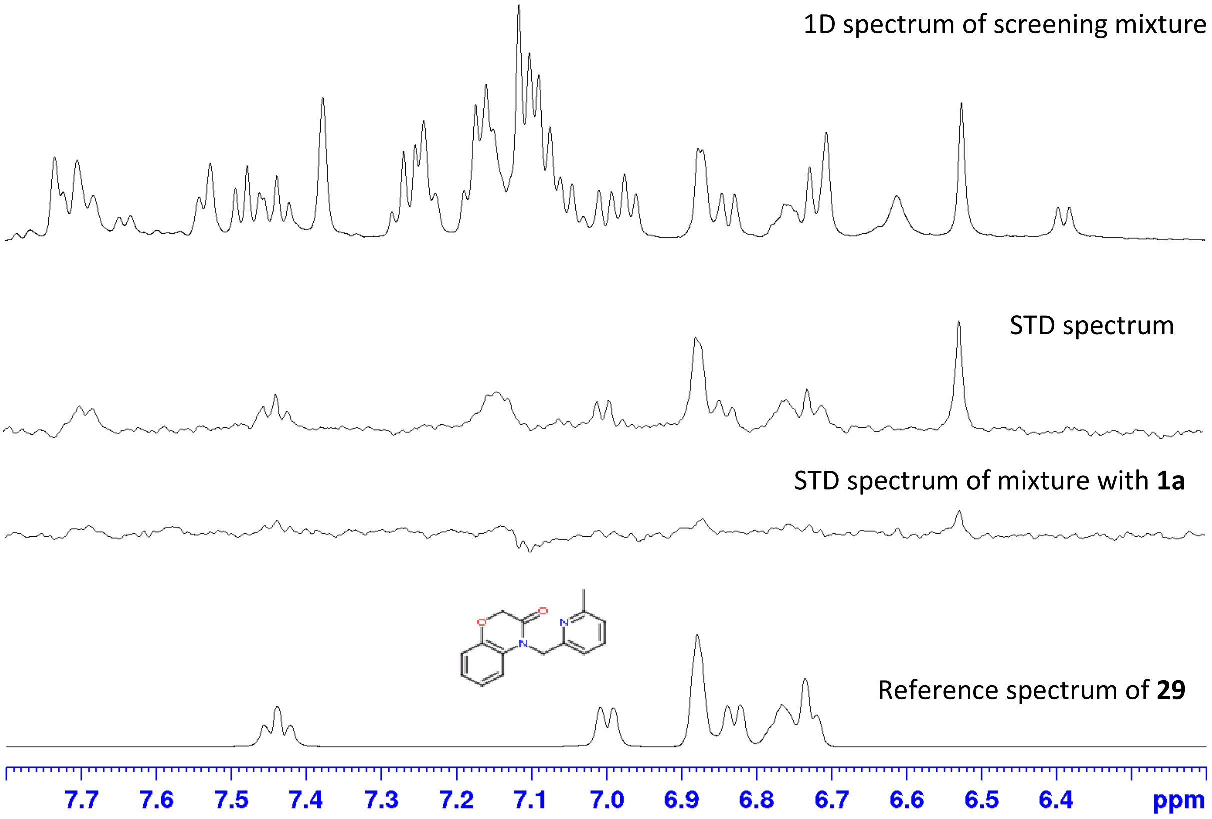

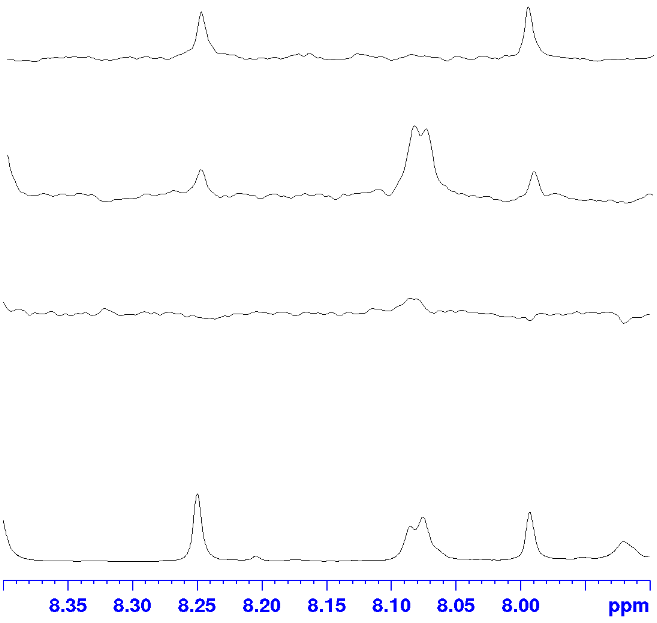

STD spectrum of NADH with InhA only.

STD spectrum of NADH with InhA and screening mixture containing **29**. Showing ~50% decrease in STD signal intensity.

STD spectrum of NADH with InhA and screening mixture containing **29** and **1a**. With further loss of intensity for the NADH STD signal intensities.

1D spectrum of screening mixture showing NADH signals.

32

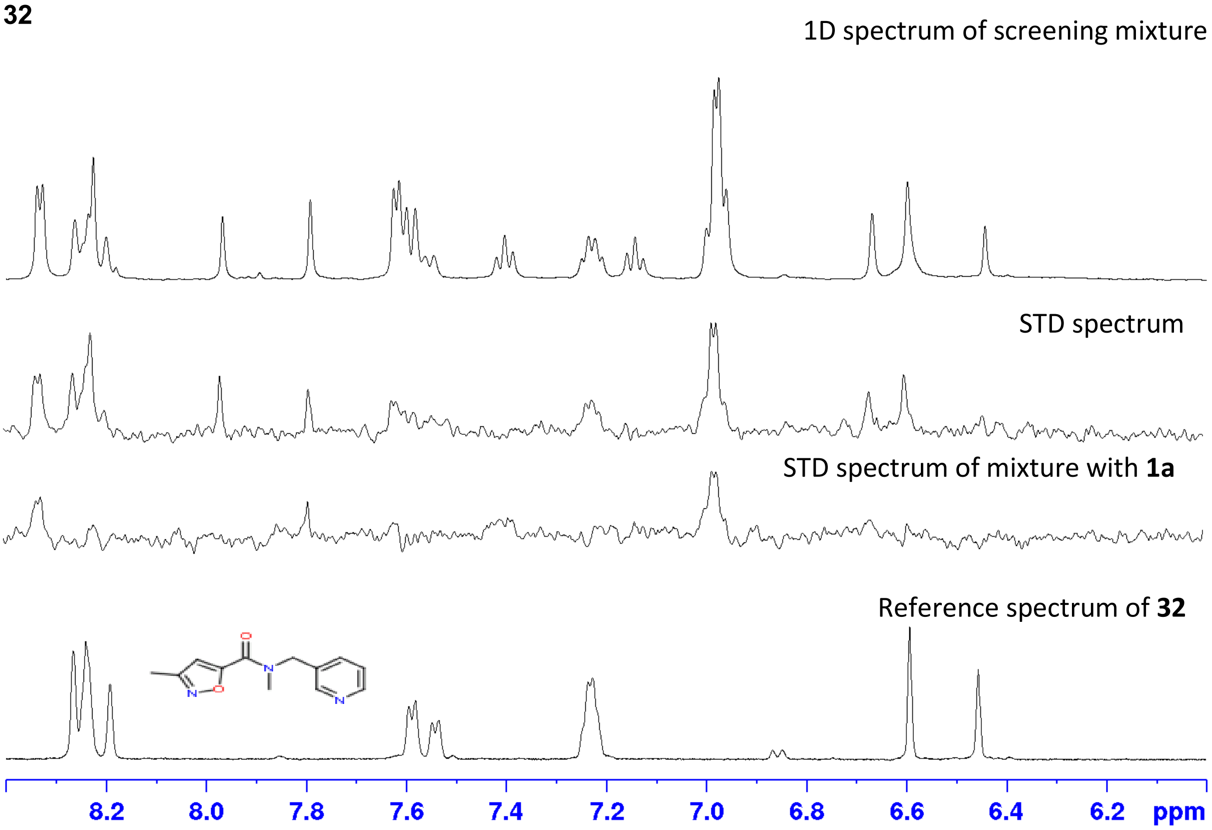
1D spectrum of screening mixture
STD spectrum
STD spectrum of mixture with **1a**
Reference spectrum of **32**
8.2
8.0
7.8
7.6
7.4
7.2
7.0
6.8
6.6
6.4
6.2
ppm

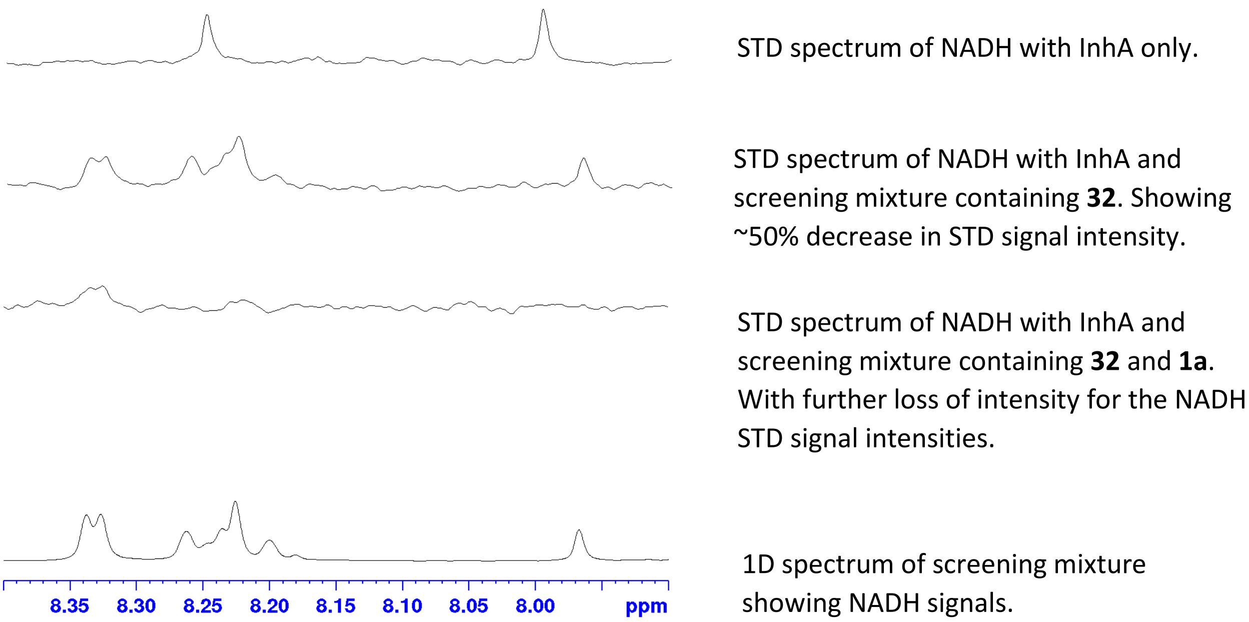
STD spectrum of NADH with InhA only.
STD spectrum of NADH with InhA and screening mixture containing **32**. Showing ~50% decrease in STD signal intensity.
STD spectrum of NADH with InhA and screening mixture containing **32** and **1a**. With further loss of intensity for the NADH STD signal intensities.
1D spectrum of screening mixture showing NADH signals.
8.35
8.30
8.25
8.20
8.15
8.10
8.05
8.00
ppm

33

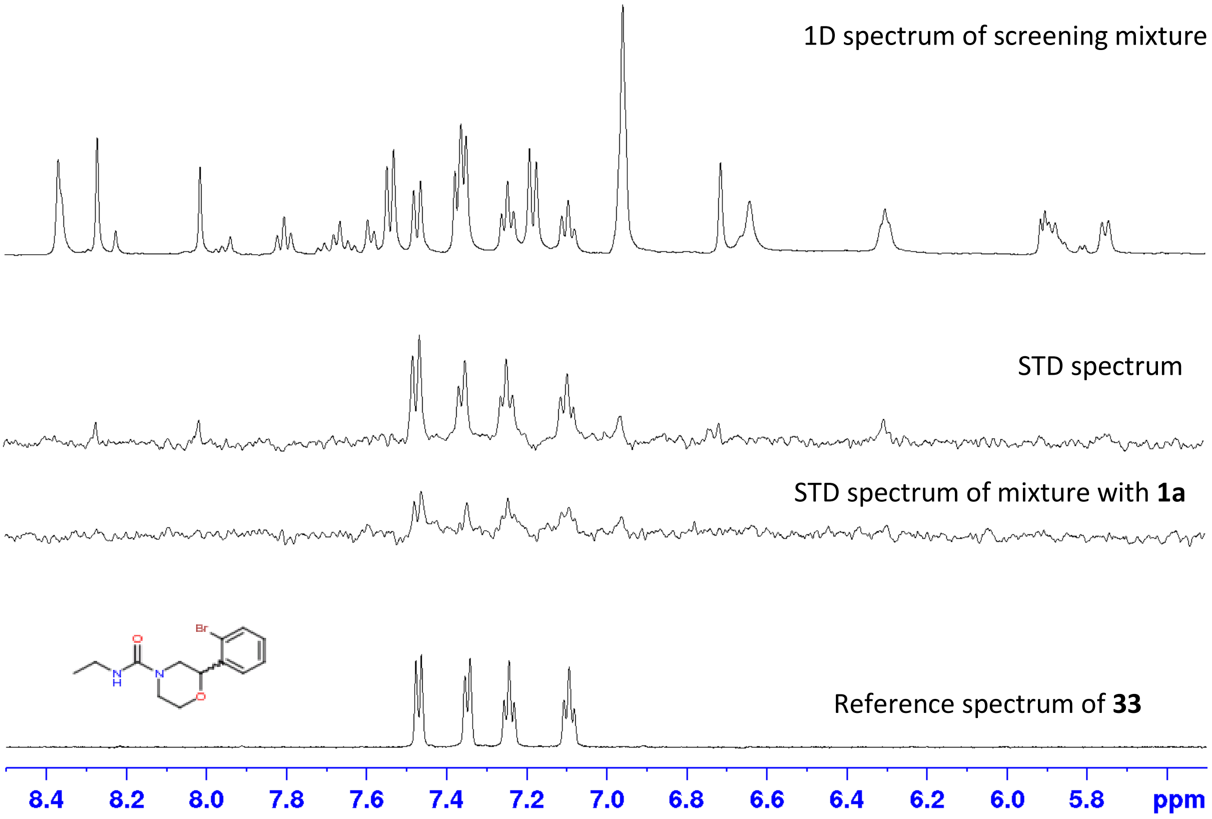
1D spectrum of screening mixture
STD spectrum
STD spectrum of mixture with **1a**
Br
O
N
H
N
O
Reference spectrum of **33**
8.4
8.2
8.0
7.8
7.6
7.4
7.2
7.0
6.8
6.6
6.4
6.2
6.0
5.8
ppm

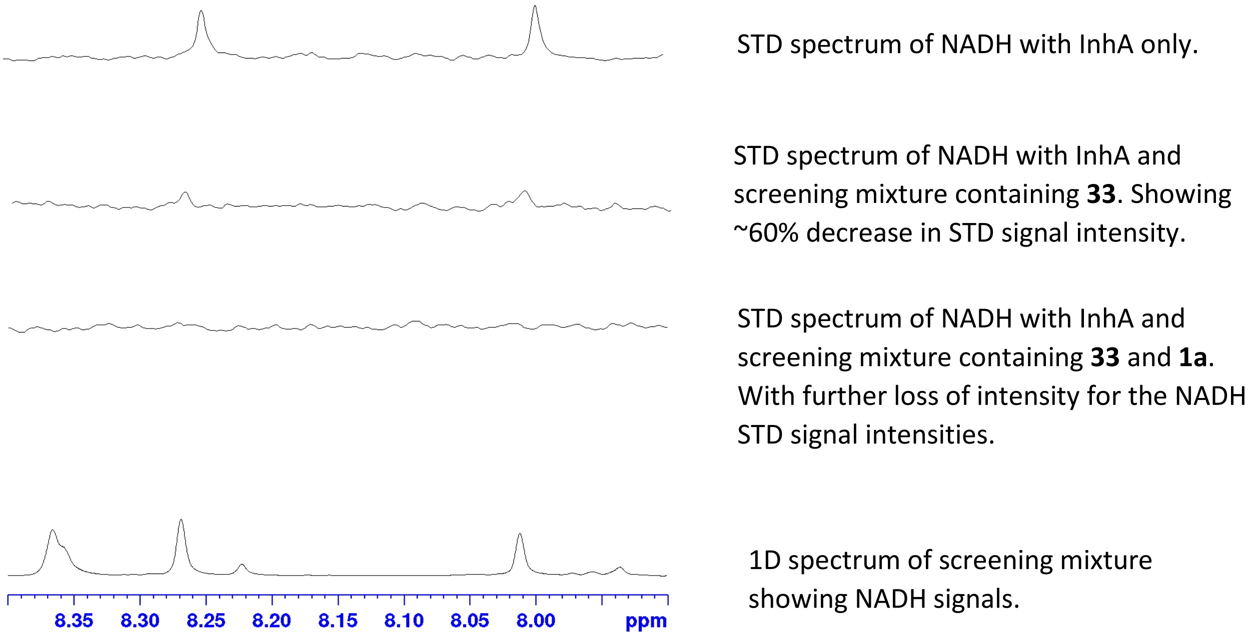
STD spectrum of NADH with InhA only.
STD spectrum of NADH with InhA and screening mixture containing **33**. Showing ~60% decrease in STD signal intensity.
STD spectrum of NADH with InhA and screening mixture containing **33** and **1a**. With further loss of intensity for the NADH STD signal intensities.
1D spectrum of screening mixture showing NADH signals.
8.35
8.30
8.25
8.20
8.15
8.10
8.05
8.00
ppm

34

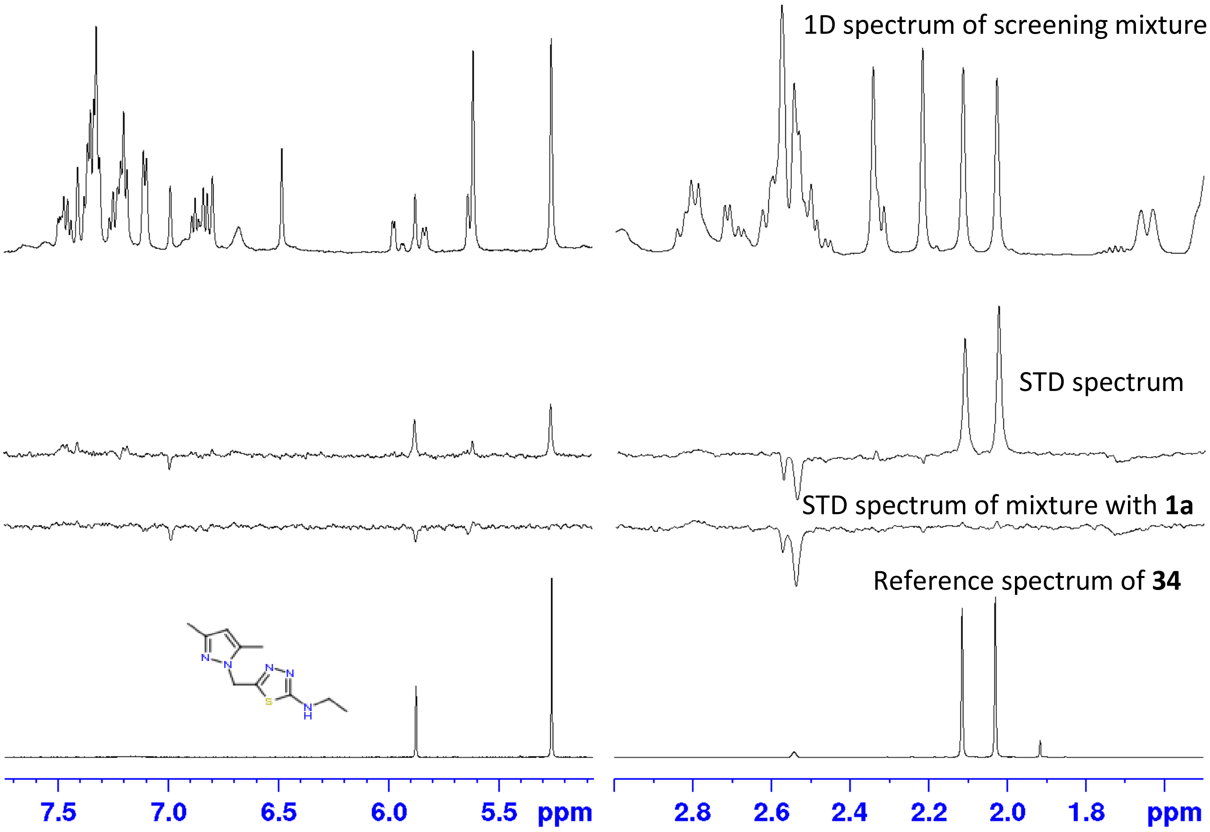

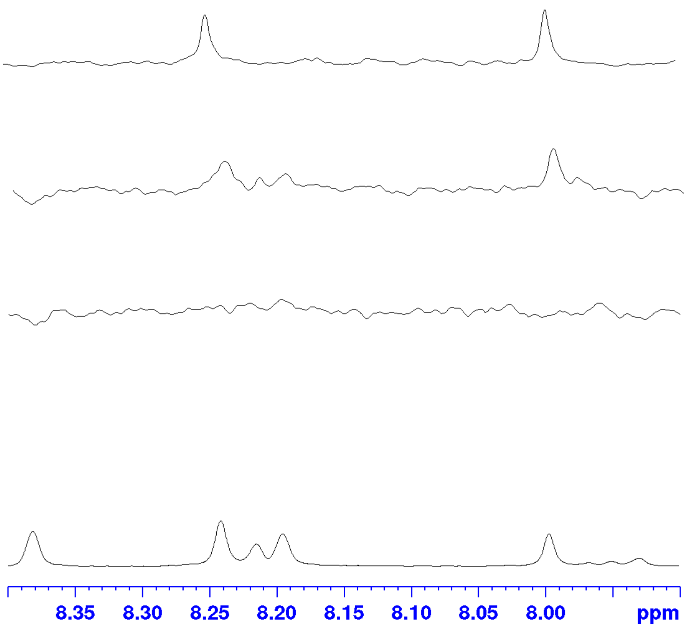

STD spectrum of NADH with InhA only.

STD spectrum of NADH with InhA and screening mixture containing **34**. Showing ~30% decrease in STD signal intensity.

STD spectrum of NADH with InhA and screening mixture containing **34** and **1a**. With further loss of intensity for the NADH STD signal intensities.

1D spectrum of screening mixture showing NADH signals.

35

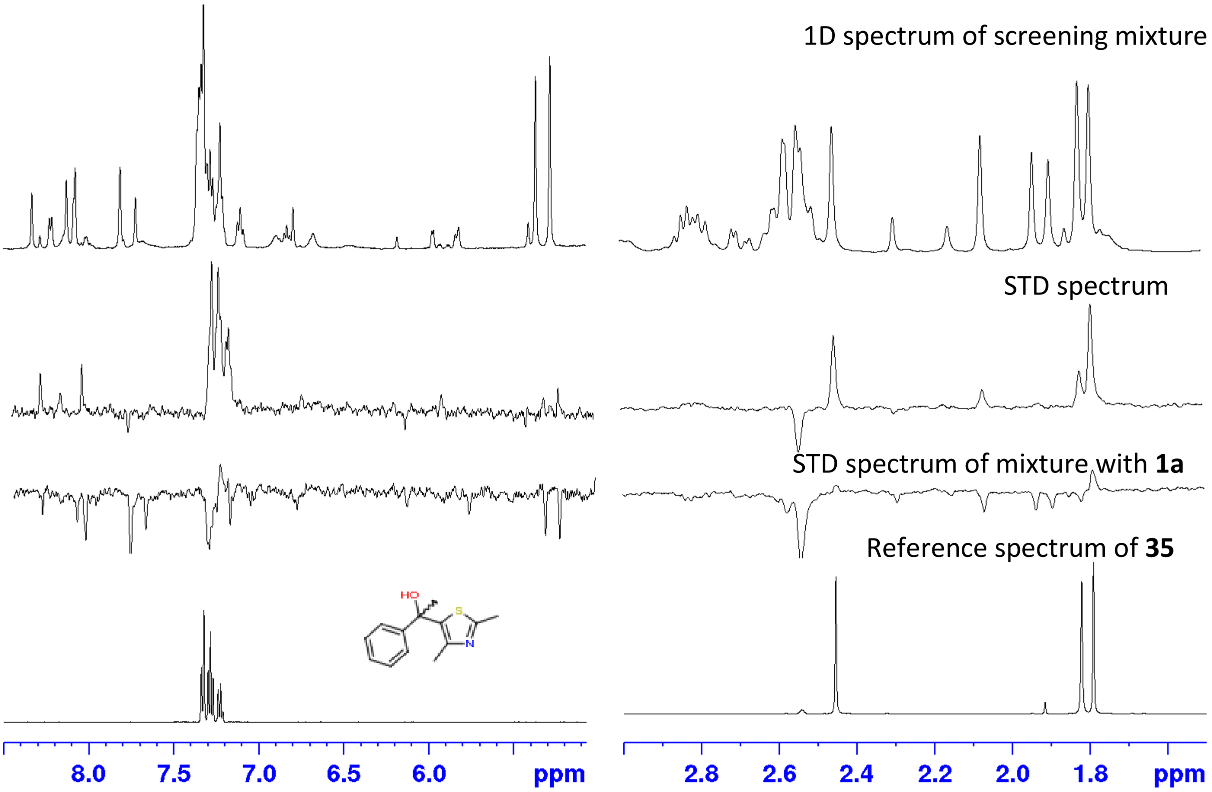
1D spectrum of screening mixture
STD spectrum
STD spectrum of mixture with 1a
Reference spectrum of 35
HO
S
N
8.0
7.5
7.0
6.5
6.0
ppm
2.8
2.6
2.4
2.2
2.0
1.8
ppm

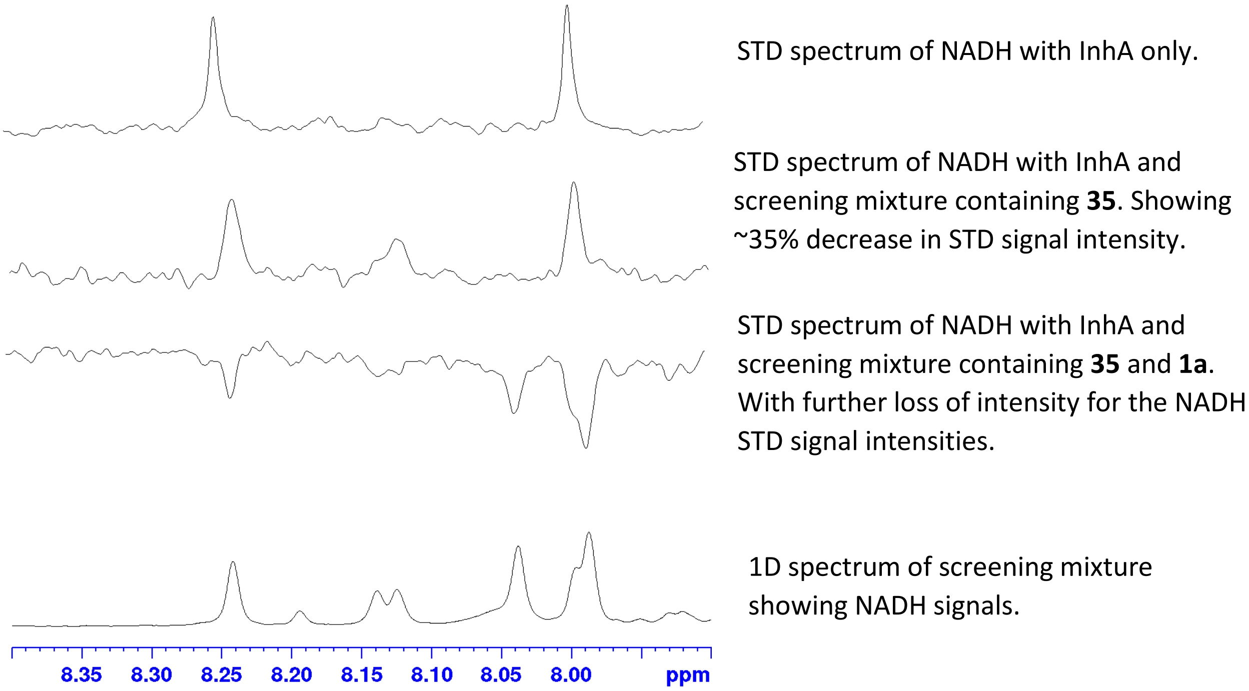
STD spectrum of NADH with InhA only.
STD spectrum of NADH with InhA and screening mixture containing 35. Showing ~35% decrease in STD signal intensity.
STD spectrum of NADH with InhA and screening mixture containing 35 and 1a. With further loss of intensity for the NADH STD signal intensities.
1D spectrum of screening mixture showing NADH signals.
8.35
8.30
8.25
8.20
8.15
8.10
8.05
8.00
ppm

InhA enzymatic assay

The InhA enzymatic activity was measured fluorimetrically by following NADH oxidation at λ_{ex}=525 nm and λ_{em}=598 nm, by means of the resazurin/diaphorase/NADH coupled-enzyme reaction.
Experiments were performed using 30 µm B-NADH and 50 µm 2-*trans*-dodecenoyl-CoA (DDCoA) as substrates, and 2.5 nm InhA[6]. All reactions were run in 30 mm PIPES buffer (pH 6.8) and 0.2 mg mL^{-1} BSA at RT.
149 STD-NMR hits were tested at 500 µM single point concentration in duplicate. Compounds tested in dose-response experiments to determine IC_{50} values were screened at concentrations up to 1mM in duplicate.
Percent remaining enzyme activity (% AR) at different compound concentrations were calculated according to the formula:

$$\% \ AR=100\times\{(sample-ctrl_2)/(ctrl_1-ctrl_2)\}$$

where *sample* is the enzyme activity for each compound concentration, $ctrl_1$ is enzyme activity in the absence of test compound, and $ctrl_2$ is B-NADH oxidation in the absence of enzyme. IC_{50} values were calculated by fitting % AR to a two-parameter equation:

$$\% \ AR=100/\{1+([\text{compd}]/IC_{50})\times s\}$$

where s is a slope factor. IC_{50} values were calculated using the ActivityBase XE nonlinear regression function in the full curve analysis bundle or a similar program.

InhA informed fragment set

In order to identify Mtb InhA relevant compounds the Protein Data Bank (PDB, http://www.rcsb.org), ChEMBL (https://www.ebi.ac.uk/chembl/) and GoStar (https://www.gostardb.com/) databases were searched using a combination of text queries:

- Preferred name: Enoyl-[acyl-carrier-protein] reductase
- Synonyms: Enoyl-[acyl-carrier-protein] reductase [NADH], NADH-dependent enoyl-ACP reductase, inhA
- Organism: Mycobacterium tuberculosis
- Protein Target Classification: enzyme > oxidoreductase
- ChEMBL Target ID: CHEMBL1849
- UniProtID: P9WGR1

A total of 639 unique compounds were identified covering a diverse set of chemical scaffolds (compounds were grouped in 118 clusters based on similarity using FCFP4 fingerprints as molecular descriptors and a threshold of 0.4 Tanimoto index).

The 639 InhA compounds were fragmented in using the “enumerate fragments” component in Pipeline Pilot (default settings - http://accelrys.com/products/collaborative-science/biovia-pipeline-pilot/).

A total of 2257 unique fragments were generated. The set was reduced to 832 compounds by applying the following filters:

- Num_heavyatoms ≥ 10 and Num_heavyatoms ≤ 20
- Num_rings ≥ 1

Two collections of commercially available compounds (eMolecules, https://www.emolecules.com/ and MolPort, https://www.molport.com/) accounting for more than 10 million chemical entities were searched and 312 fragments were identified as available for acquisition.

Visual inspection resulted in removal unsuitable fragments (e.g. all carbon atoms fragments or fragments with little functionalization) as well as multiples of closely related analogues. This process led to a set of 232 fragments. There was further attrition during the compound acquisition process related to factors such as lack of required sample amount (at least 5 mg of solid sample), delivery time and unacceptably high costs of individual fragments. Additional attrition occurred during the quality control check, previously described[8] resulting in a final set of 46 InhA-informed fragment compounds suitable for STD-NMR screening.

Crystallography Methods

Protein Preparation

Untagged InhA (with Thr at position 2 mutated to Ala) was expressed in *E.coli* and purified in two steps by IEC (Resource 15Q column) and Size Exclusion (HiLoad Superdex 75 Prep-grade column) chromatography. Purified protein at approximately 8 mgs/mL in 25 mM Tris pH8, 2 mM DTT, 150 mM NaCl was incubated with 2 mM NAD prior to crystallisation.

Crystal preparation

Apo crystals were grown from 35-42% ethoxyethanol and 0.1M Mes pH 6.5 - 6.8 in 150 nL + 150 nL sitting drops. Crystals with a number of different morphologies grew simultaneously in the drops. Soaking of hexagonal and non-hexagonal shaped crystals was attempted.

Crystals were soaked for 2h in 36% ethoxyethanol, 5% glycerol and 0.1M Mes pH 6.5 diluted 5% with compound DMSO stock solution. DMSO stock solutions were a nominal 1M.

Crystals were frozen straight from the soaking solution.

For co-crystallisation (marked as CoX in the table), the prepared protein was incubated with an excess of compound (20 mM) for 2h and then spun down at 13k rpm for 10 min prior to setting-up standard commercial screens with 100 nL + 100 nL sitting drops. Details of the successful condition for each compound is given in the Table along with details of how the crystal was frozen.

Data Collection

Data was collected in-house on a Rigaku FRE+ generator with A200 detector, at the Diamond Light Source (Oxford), on beamlines i03, i04 and i04-1 and at the European Synchrotron Radiation Facility (Grenoble), on beamline ID23 eh1. Synchrotron beamlines were equipped with Pilatus detectors.

Data processed automatically at the synchrotrons using protocols based on XDS,[9] AIMLESS,[10] and the CCP4 suite of programs[11] and CC1/2 of 0.5[12] used to determine resolution cut-of were used unmodified. In-house data were processed using autoPROC.[13] Data collection statistics are given in the Table.

Structure Determination and Refinement

The structures were determined using either an unpublished unliganded protein model for the hexagonal crystal or a published structure with appropriate cell dimensions. Coot[14] was used for model building and refinement was carried out with autoBUSTER.[15] Libraries were generated using ELBOW[16] and the geometry checked using MOGUL.[17] The final model statistics are given in the Table. The coordinates and structure factors have been deposited in the Protein Data Bank with accession codes noted in the Table.

Table of X-ray Statistics, Part 1

	4	9	12	22	24	34
**Crystallisation**						
Crystallisation condition	Standard soaking protocol	CoX. MBC1 E4; 2.17% MPD, 0.3M $Mg(NO_3)_2$, 0.1 M Tris pH 8 20% PEG2K Cryo - well solution	Standard soaking protocol	Standard soaking protocol	Standard soaking protocol	Standard soaking protocol
**Data collection**						
Synchrotron and beamline	In-house	Diamond I04	In-house	In-house	Diamond I03	In-house
Space Group	P6222	C2	P6222	P6222	P212121	I212121
Cell Dimensions a,b,c (Å) α, β, γ (°)	97.27, 97.27, 139.92 90, 90, 120	73.39, 113.86, 65.75 90, 113.86, 90	97.5, 97.5, 140.32 90, 90, 120	97.48, 97.48, 139.95 90, 90, 120	92.07, 101.08, 186.96 90, 90, 90	91.39, 99.49, 185.67 90, 90, 90
**Data Processing**						
Data scaling program	autoPROC/ Aimless	XDS/ Aimless	autoPROC/ Aimless	autoPROC/ Aimless	autoPROC/ Aimless	autoPROC/ Aimless
Resolution (Å) Overall (high resolution shell)	72.17-1.87 (2.05-1.87)	56.93-2.03 (2.08-2.03)	46.77-2.76 (2.95-2.76)	84.42-1.91 (2.09-1.91)	82.60-2.82 (2.89-2.82)	92.84-2.74 (3.87-2.74)
$R_{merge}{}^{b}$	0.112(0.731)	0.036 (0.812)	0.251(0.884)	0.144(0.859)	0.123(2.080)	0.112(0.171)
CC(1/2)	0.999(0.9)	0.976(0.531)	0.986(0.818)	0.998(0.855)	0.998(0.474)	0.926(0.843)
Mean $I/\sigma I$	17.8(3.0)	7.2(1.5)	9.6(2.6)	15.9(3.1)	11.3(1.0)	12.4(8.1)
Completeness (%)	99.9(100)	95.1(98.2)	99.9(99.9)	99.7(99.2)	100(10)	97.9(99.1)
Redundancy	7.8(7.7)	3.3(3.3)	7.2(7.4)	7.8(7.9)	6.6(6.6)	2.8(2.8)
Total No. observations	255809(59615)	106201(8177)	77128(13987)	243804(56942)	282578(20839)	62244(40326)
Total No. Unique observations	32910(7730)	32020(2444)	10717(1885)	31199(7250)	42892(3145)	22089(14355)
**Refinement**						
Refinement program	BUSTER 2.11.7	BUSTER 2.11.6	BUSTER 2.11.7	BUSTER 2.11.7	BUSTER 2.11.7	BUSTER 2.11.7
Resolution (Å)	36.09-1.87	26.58-2.03	46.77-2.76	24.62-1.91	82.60-2.82	29.48-2.74
R_{work}/R_{free}	0.151/0.182	0.1767/0.2216	0.154/0.230	0.151/0.180	0.160/0.186	0.157/0.201
No. Reflections	32859	32008	10670	30957	42829	21613
Bond lengths (Å)	0.010	0.010	0.010	0.010	0.010	0.010
Bond angles (°)	0.980	1.07	1.16	0.99	1.10	1.12
**No. of Atoms**						
Protein	2027	3896	2005	2027	7843	3959
Ligand	14	28	17	15	48	32
Water	398	319	207	376	77	321
**B factors**						
Protein	22.529	28.352	30.220	21.251	86.808	34.879
Ligand	26.027	34.760	31.188	20.689	103.212	55.741
Water	45.298	36.315	36.293	43.713	72.093	35.763
**PDB code**	5OIC	5OIF	5OIL	5OIM	5OIN	5OIO

Table of X-ray Statistics, Part 2

	37	40	41	46	47
**Crystallisation**					
Crystallisation condition	CoX JCSG+ C9; 10% glycerol, 25% 1,2 propanediol, 0.1M PO4. Cryo – well solution	CoX MBC1 D11; 0.5M NaCl 0.1M NaH_2PO_4 pH 6.8 15% w/v PEG2K. Cryo - well + 20% glycerol	Standard soaking protocol	CoX JCSG+ E10; 10% PEG6K, 0.1M Bicine, pH 9. Cryo – well + 25% ethylene glycol	CoX MBC1 H7; 0.2M $(NH_4)_2SO_4$, 30% PEG4K. Cryo - well + 30% ethylene glycol
**Data collection**					
Synchrotron and beamline	Diamond I04-1	ESRF ID23-1	In-house	Diamond I03	Diamond I04
Space Group	P6222	P21	P6222	P212121	P212121
Cell Dimensions a,b,c (Å) α, β, γ (°)	99.14, 99.14, 141.52<br>90, 90, 120	64.97, 115.04, 69.05<br>90, 97.44, 90	96.66, 96.66, 140.758<br>90, 90, 120	105.54, 103.51, 177.22<br>90, 90, 90	104.83, 103.06, 180.98<br>90, 90, 90
**Data Processing**					
Data scaling program	XDS/ Aimless	autoPROC/ Aimless	autoPROC/ Aimless	XDS/ Aimless	XDS/ Aimless
Resolution (Å) Overall (high resolution shell)	41.34-1.71 (1.75-1.71)	58.84-2.65 (3.24-2.65)	83.71-1.97 (2.2-1.97)	41.53-2.24 (2.30-2.24)	68.5-2.58 (2.65-2.58)
R_{merge}[b]	0.101(2.876)	0.074(0.093)	0.188(0.803)	0.083(1.371)	0.140(2.019)
CC(1/2)	1.0(0.514)	0.862(0.841)	0.995(0.802)	0.999(0.526)	0.997(0.515)
Average $I/\sigma I$	19.6(1.3)	18.9(15.9)	11.5(2.9)	13.3(1.4)	11.3(1.1)
Completeness (%)	99.9(100)	97.3(96.8)	100(100)	99.2(99.7)	99.9(99.6)
Redundancy	19.2(17.1)	2.5(2.5)	7.2(7.2)	6.7(6.8)	6.7(7.0)
No. Reflections	862877(55546)	71383(31723)	204573(57095)	624148(46436)	419636(32051)
No. Unique Reflections	45004(3248)	28541(12880)	28378(7898)	93202(6864)	62385(4549)
**Refinement**					
Refinement program	BUSTER 2.11.7	BUSTER 2.11.6	BUSTER 2.11.7	BUSTER 2.11.7	BUSTER 2.11.7
Resolution (Å)	25.04-1.71	57.52-2.65	24.13-1.97	41.53-2.24	68.50-2.58
R_{work}/R_{free}	0.160/0.173	0.160/0.227	0.153/0.189	0.171/0.189	0.168/0.200
No. Reflections	44906	28392	28044	93102	62259
Bond lengths (Å)	0.010	0.010	0.010	0.010	0.010
Bond angles (°)	0.99	1.12	1.05	1.08	1.16
**No. of Atoms**					
Protein	2056	7543	2036	7976	7976
Ligand	23	60	21	104	112
Water	304	621	353	464	450
**B factors**					
Protein	33.283	17.779	20.724	69.861	63.931
Ligand	41.460	14.178	17.454	70.754	67.315
Water	55.859	20.111	41.967	64.822	61.139
**PDB code**	5OIP	5OIQ	5OIR	5OIS	5OIT

Supplementary Figures

Fit to density and comparison of two binding sites.

Left hand panel – Fo-Fc omit map around exemplar ligand using final model for protein. Map contoured at +/-3rms, green positive and red negative contours

Right hand panel – Comparison of the modelled ligand in the two independent binding sites in the crystal asymmetric unit.

**4**	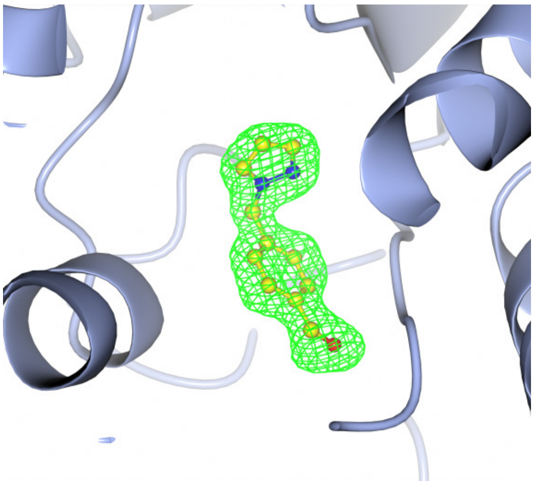	
**9**	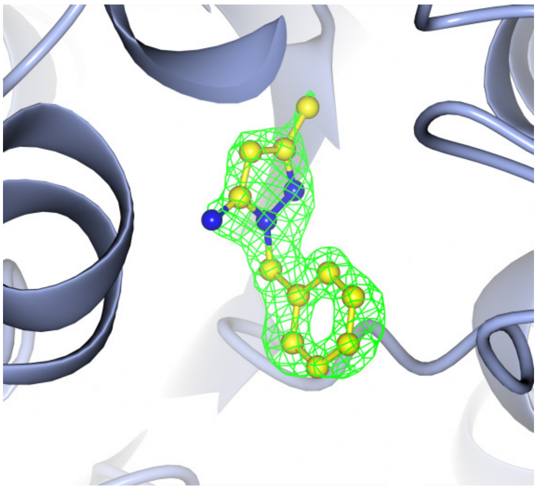	2 protomers in asymmetric unit 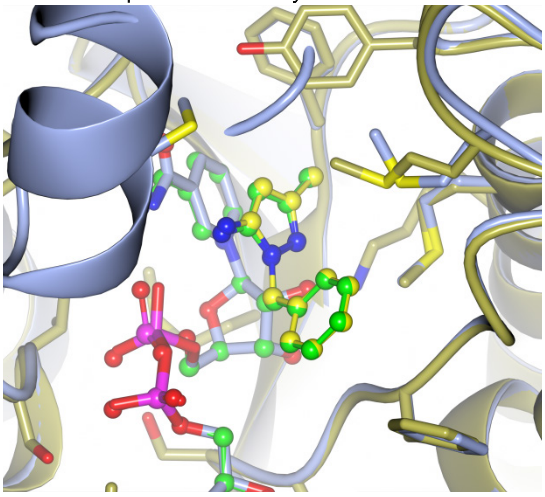

<table>
<tr><td>12</td><td>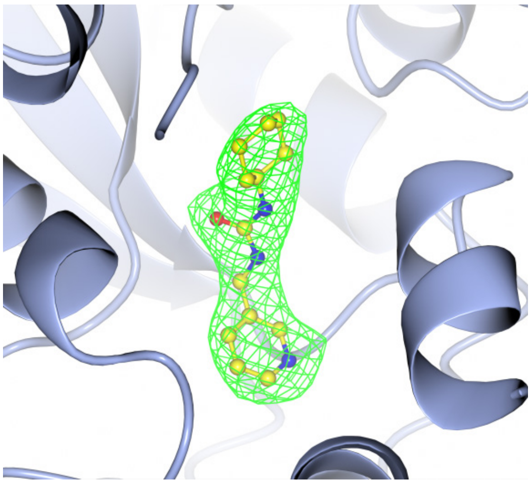</td><td></td></tr>
<tr><td>22</td><td>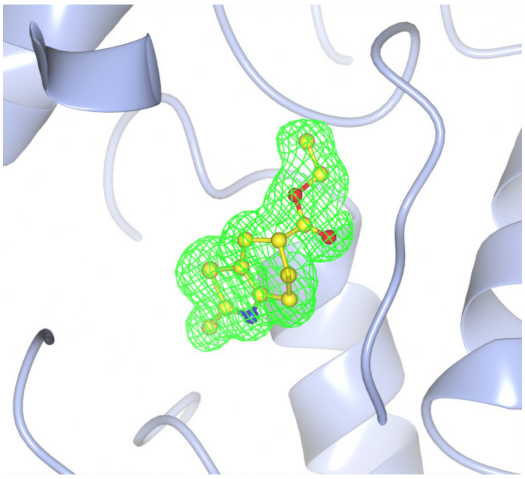</td><td></td></tr>
<tr><td>24</td><td>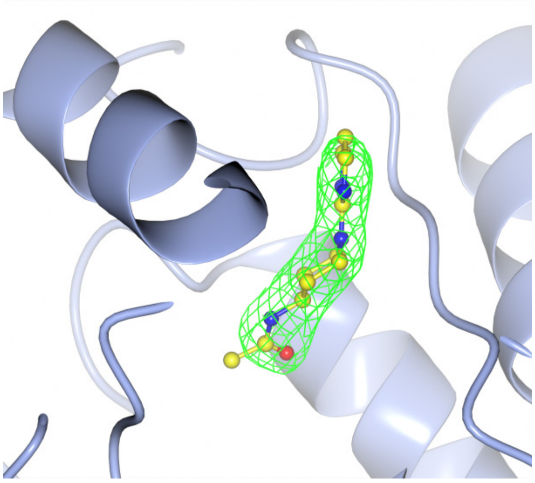</td><td>Tetramer in asymmetric unit. Ligand bound in only 3 binding sites.
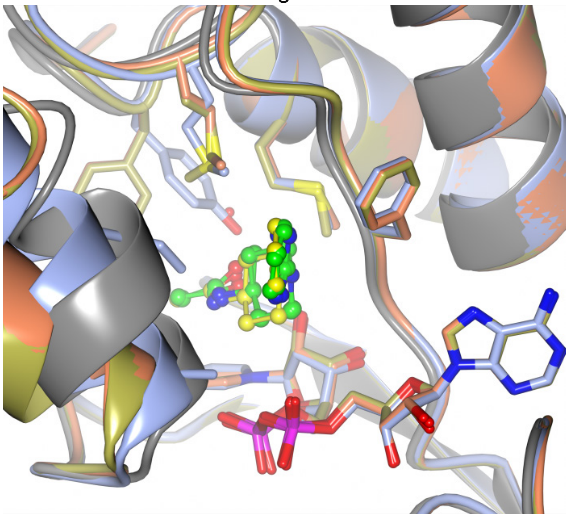</td></tr>
</table>

**34**	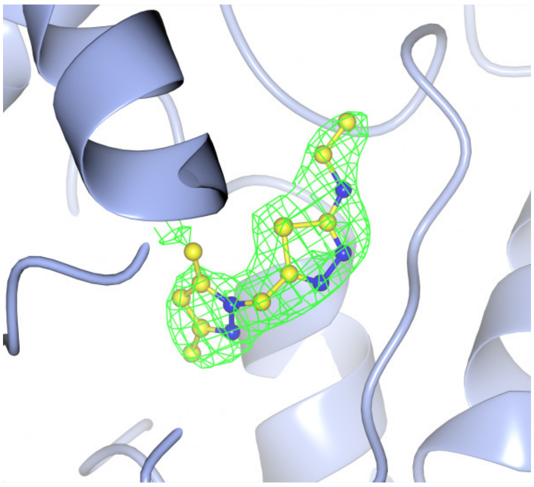	2 protomers in asymmetric unit<br>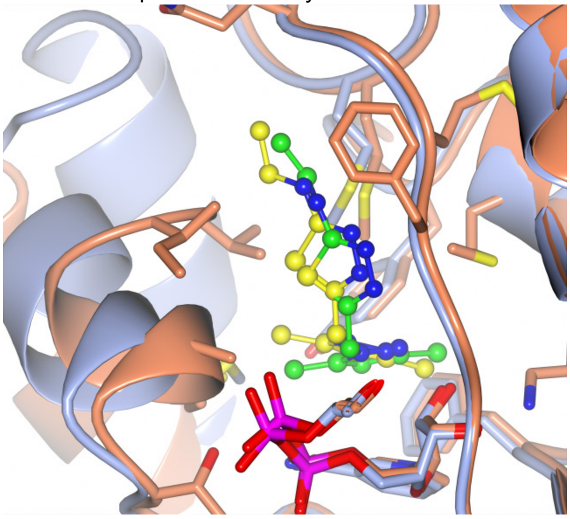
**37**	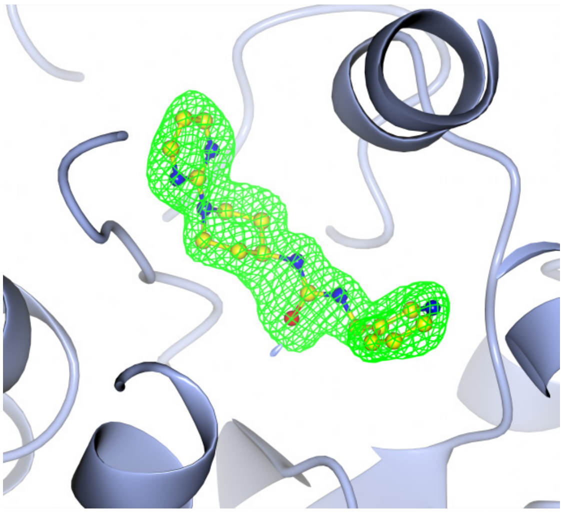	
**40**	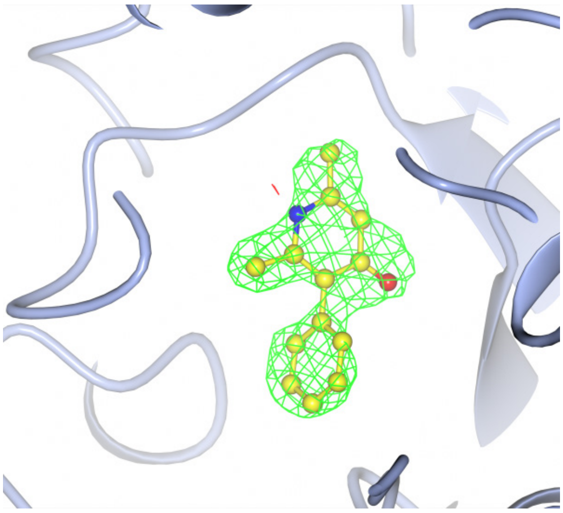	Tetramer in asymmetric unit<br>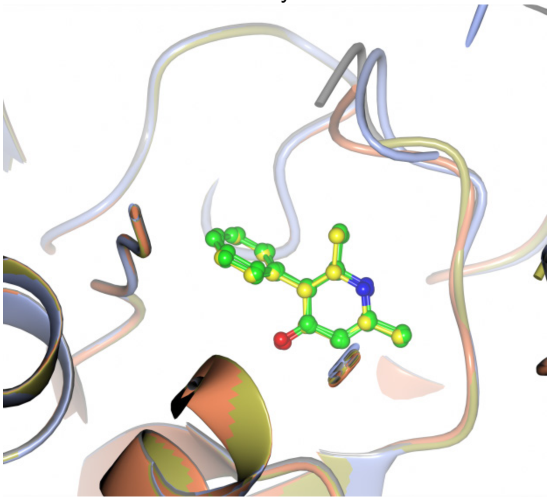

**41**	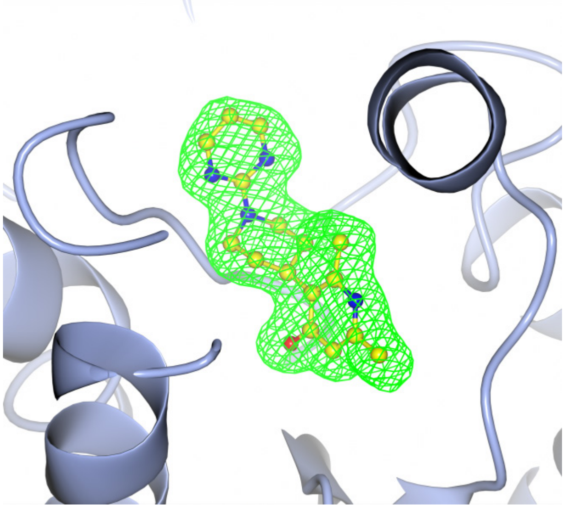	
**46**	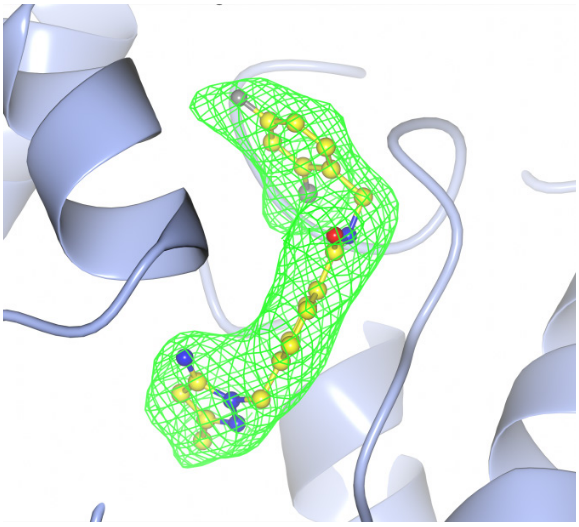	Tetramer in asymmetric unit 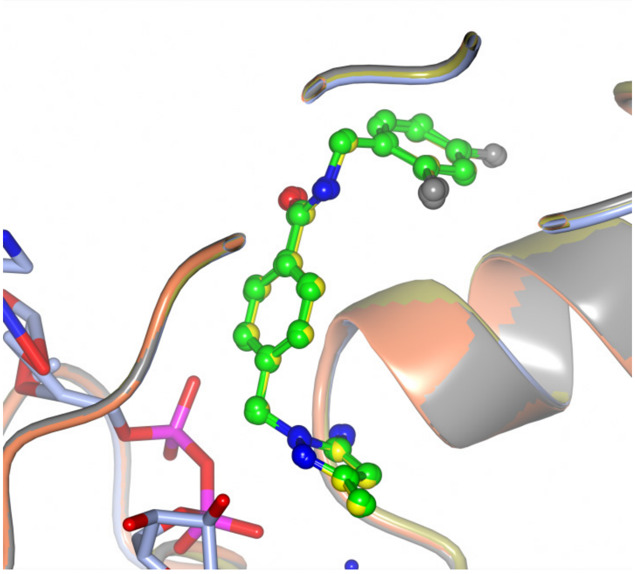
**47**	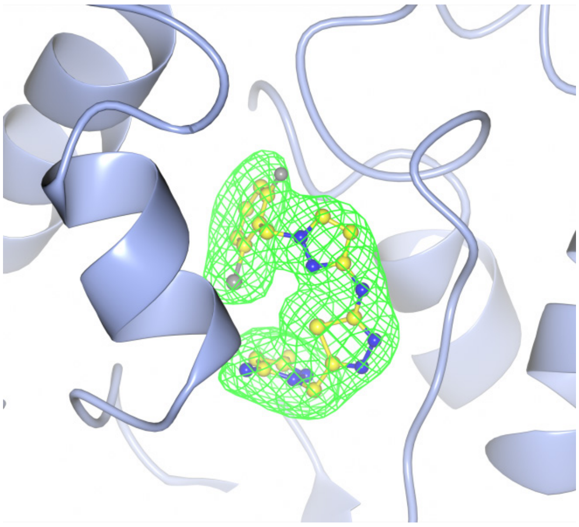	Tetramer in asymmetric unit 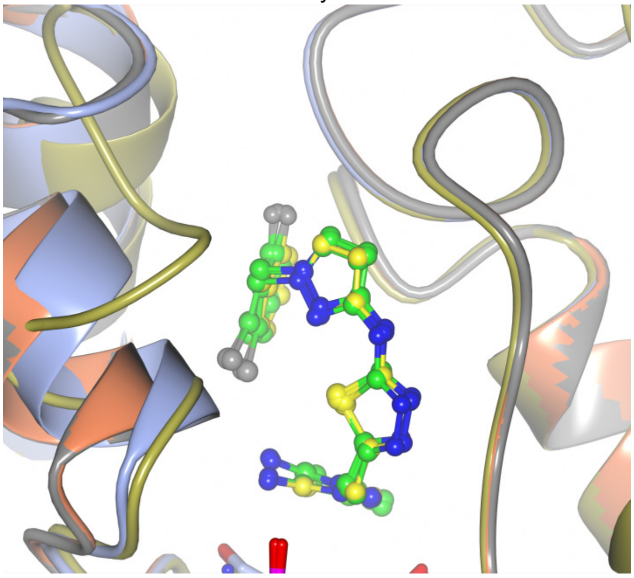

SPR assay

Method:

All experiments were performed on Biacore 4000 with sample chamber temperature at 20°C and analysis temperature at 25°C.

1. Immobilisation of InhA protein

CM5 SPR sensor chips (GE healthcare) were primed with an immobilisation buffer of 10 mM HEPES and 150 mM NaCl, pH 7.4 prior to activation with a 420s injection at 10 μL/min of 0.4 M N-ethyl-N'-(dimethylaminopropyl)carbodiimide) (EDC) and 0.1 M N-Hydroxysuccinimide (NHS). 100 μg/mL InhA in 10 mM sodium acetate pH 5.5 was then injected at 10 μL/mL across the activated flow cell. Unreacted NHS-esters were quenched by an injection of 100 mM Tris-HCl, pH 8.5 for 420 s. Typically an immobilisation level of 5kRU was achieved.

2. Affinity analysis

Binding data was obtained by serial injection of 3-fold increasing concentrations of compounds up to 1 mM over the sensor chip surface. An assay running buffer of 20 mM HEPES, 150 mM NaCl, 0.05% Tween 20 and 1-2% DMSO at pH 7.4 with or without 30 μM NADH was used. The NADH concentration was chosen to saturate the NADH binding site (Kd of ~ 400 nM). All sensograms were double reference subtracted and solvent corrected. The equilbrium binding levels were analysed by a TIBCO Spotfire template where pKd values were determined by fitting to a standard 1:1 interaction model. Each compound was run in duplicate (technical replicate) and repeated in two separate runs (experimental replicate).

Supplementary data:

Representative sensograms (top) and dose response curves (bottom) are shown for each compound.

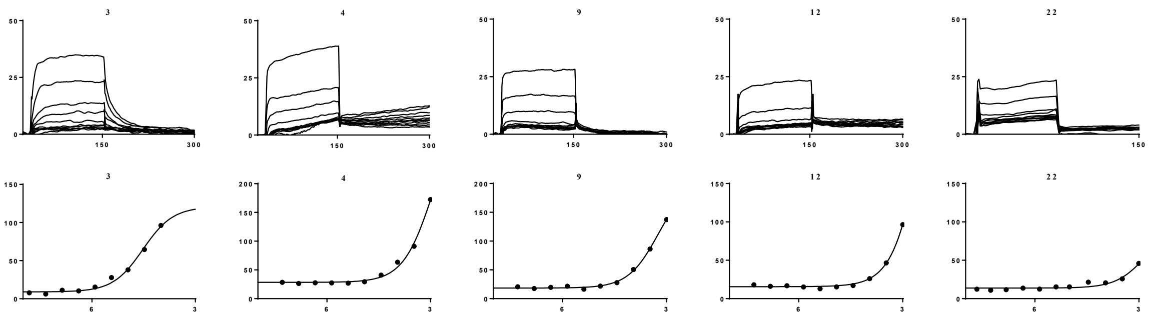

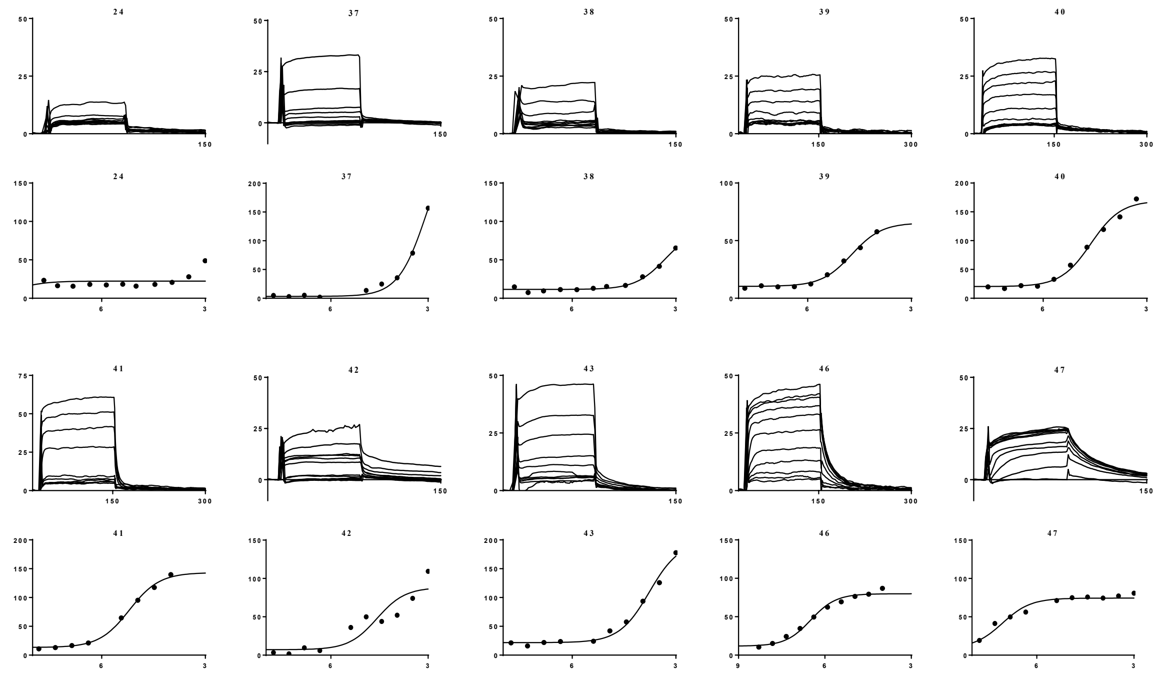

Sensograms: *Dose response curves:*

Y axis: Binding responses (RU) *Y axis: Binding (% of control binding)*

X axis: Time (s) *X axis: -log[compound]*

For samples, the binding (% of control compound binding) is the binding response adjusted based on the compound molecular weight and scaled based on the interpolated positive control value. pKd values were calculated by fitting binding (% of control compound binding) to a dose-response model, with the slope parameter constrained to 1:

$$Y = ([ASYMMIN] + (([ASYMMAX] - [ASYMMIN]) / (1 + (((10 ^ {\wedge} ([pKd] * -1)) / (10 ^ {\wedge} ([x] * -1))) ^ {\wedge} [SLOPE]))))/100$$

Where Y is the “Binding (% of control compound binding), x equals -log of compound concentration, ASYMMIN is the fitted bottom signal and ASYMMAX is the fitted top signal.

The pKd values with 95% confidence interval > 1 were not reported. Rmax is calculated by substracting ASYMMIN from ASYMMAX. Fittings with a Rmax (% of control) > 250% or < 40% were not reported with a pKd value.

References

[1] C. Spyropoulos, C. G. Kokotos, *J Org Chem* **2014**, *79*, 4477.

[2] aP. S. Ng, U. H. Manjunatha, S. P. Rao, L. R. Camacho, N. L. Ma, M. Herve, C. G. Noble, A. Goh, S. Peukert, T. T. Diagana, P. W. Smith, R. R. Kondreddi, *Eur J Med Chem* **2015**, *106*, 144; bR. R. Kondreddi, U. H. Manjunatha, L. M. Ngai, S. Peukert, S. P. S. Rao, **2014**, WO2014093606.

[3] A. Guardia, G. Gulten, R. Fernandez, J. Gomez, F. Wang, M. Convery, D. Blanco, M. Martinez, E. Perez-Herran, M. Alonso, F. Ortega, J. Rullas, D. Calvo, L. Mata, R. Young, J. C. Sacchettini, A. Mendoza-Losana, M. Remuinan, L. Ballell Pages, J. Castro-Pichel, *ChemMedChem* **2016**, *11*, 687.

[4] L. Ballell Pages, J. Castro Pichel, R. Fernandez Menendez, E. P. Fernandez Velando, S. Gonzalez del Valle, M. L. Leon Diaz, A. Mendoza Losana, M. J. Wolfendale, **2010**, WO2010118852.

[5] T. L. Hwang, A. J. Shaka, *J Magn Reson Ser A* **1995**, *112*, 275.

[6] M. Martinez-Hoyos, E. Perez-Herran, G. Gulten, L. Encinas, D. Alvarez-Gomez, E. Alvarez, S. Ferrer-Bazaga, A. Garcia-Perez, F. Ortega, I. Angulo-Barturen, J. Rullas-Trincado, D. Blanco Ruano, P. Torres, P. Castaneda, S. Huss, R. Fernandez Menendez, S. Gonzalez Del Valle, L. Ballell, D. Barros, S. Modha, N. Dhar, F. Signorino-Gelo, J. D. McKinney, J. F. Garcia-Bustos, J. L. Lavandera, J. C. Sacchettini, M. S. Jimenez, N. Martin-Casabona, J. Castro-Pichel, A. Mendoza-Losana, *EBioMedicine* **2016**, *8*, 291.

[7] M. Mayer, B. Meyer, *Angew Chem Int Edit* **1999**, *38*, 1784; *Angew Chem* **1999**, *111*, 1902.

[8] P. C. Ray, M. Kiczun, M. Huggett, A. Lim, F. Prati, I. H. Gilbert, P. G. Wyatt, *Drug Discov Today* **2017**, *22*, 43.

[9] W. Kabsch, *Acta Crystallogr Sect D: Biol Crystallogr* **2010**, *66*, 125.

[10] P. R. Evans, G. N. Murshudov, *Acta Crystallogr Sect D: Biol Crystallogr* **2013**, *69*, 1204.

[11] M. D. Winn, C. C. Ballard, K. D. Cowtan, E. J. Dodson, P. Emsley, P. R. Evans, R. M. Keegan, E. B. Krissinel, A. G. Leslie, A. McCoy, S. J. McNicholas, G. N. Murshudov, N. S. Pannu, E. A. Potterton, H. R. Powell, R. J. Read, A. Vagin, K. S. Wilson, *Acta Crystallogr Sect D: Biol Crystallogr* **2011**, *67*, 235.

[12] P. A. Karplus, K. Diederichs, *Curr Opin Struct Biol* **2015**, *34*, 60.

[13] C. Vonrhein, C. Flensburg, P. Keller, A. Sharff, O. Smart, W. Paciorek, T. Womack, G. Bricogne, *Acta Crystallogr Sect D: Biol Crystallogr* **2011**, *67*, 293.

[14] P. Emsley, B. Lohkamp, W. G. Scott, K. Cowtan, *Acta Crystallogr Sect D: Biol Crystallogr* **2010**, *66*, 486.

[15] G. Bricogne, E. Blanc, M. Brandl, C. Flensburg, P. Keller, W. Paciorek, P. Roversi, A. Sharff, O. S. Smart, C. Vonrhein, T. O. Womack, BUSTER version 2.11.6&7 ed., Global Phasing Ltd, Cambridge, UK, **2014**.

[16] N. W. Moriarty, R. W. Grosse-Kunstleve, P. D. Adams, *Acta Crystallogr Sect D: Biol Crystallogr* **2009**, *65*, 1074.

[17] I. J. Bruno, J. C. Cole, M. Kessler, J. Luo, W. D. Motherwell, L. H. Purkis, B. R. Smith, R. Taylor, R. I. Cooper, S. E. Harris, A. G. Orpen, *J Chem Inf Comput Sci* **2004**, *44*, 2133.
